# The Mediating Effects of Work Characteristics on the Relationship between Transformational Leadership and Employee Well-Being: A Meta-Analytic Investigation

**DOI:** 10.3390/ijerph19053133

**Published:** 2022-03-07

**Authors:** Friederike Teetzen, Paul-Christian Bürkner, Sabine Gregersen, Sylvie Vincent-Höper

**Affiliations:** 1 Department of Work & Organizational Psychology, University of Hamburg, 20146 Hamburg, Germany; sylvie.vincent-hoeper@uni-hamburg.de; 2 Stuttgart Center for Simulation Science, 70569 Stuttgart, Germany; paul-christian.buerkner@simtech.uni-stuttgart.de; 3 Institution for Statutory Accident Insurance and Prevention in the Health and Welfare Services, 22089 Hamburg, Germany; sabine.gregersen@bgw-online.de

**Keywords:** transformational leadership, work characteristics, job demands, job resources, meta-analysis, employee well-being

## Abstract

Evidence points to an indirect relationship between transformational leadership (TFL) and employee well-being, and numerous work characteristics have been identified as mediators. However, the relative mediating effect of different types of job resources and job demands on the TFL–well-being relationship remains unclear, rendering it impossible to determine which ones are the most influential. This study aims to provide a comprehensive analysis of the relative mediation potential of different work characteristics in the TFL–well-being relationship in multiple three-level meta-analytical structural equation models of 243 samples. Based on the JD–R Model, this study extends this theoretical framework by suggesting TFL as a predisposing variable that influences both job resources and job demands, leading to changes in indicators of both positive and negative employee well-being. The results show that, while all the examined job resources and demands mediated the TFL–well-being relationship, organizational resources were identified as the strongest mediators. Furthermore, job demands had a strong mediating effect on the relationship between TFL and negative well-being, while job resources more strongly mediated TFL and positive well-being. We present a differentiated picture of how transformational leaders can influence their employees’ well-being at the workplace, providing valuable knowledge for future research and practice.

## 1. Introduction

Knowledge regarding the maintenance of well-being in the workforce is essential for organizations in times of skill shortage, high burnout rates and aging staff. Leadership is suggested to have a notable impact on these processes [1,2]. In this regard, the literature has long associated transformational leadership (TFL) with greater levels of positive employee well-being and lower levels of negative employee well-being [1,3]. However, the argument that the TFL framework “does little to explain what exactly it is that underpins perceptions of a leader’s transformational power” [4] (p. 17) has escalated [5]. Thus, more recent research indicates an indirect influence of TFL on employees’ well-being by shaping their work characteristics [6,7].

Job demands and job resources as work characteristics are highly susceptible to influences by leaders—on the one hand, by actively amending the work environment [8] and, on the other hand, by influencing how employees view their work environment [4,9]. Accordingly, research has identified various work characteristics to mediate the TFL–well-being relationship [1,10]. However, no attempt has been made to integrate all these mediators to gain knowledge regarding which one is the most relevant. This knowledge, however, would guide researchers and practitioners in understanding how TFL leaders can best influence their employees’ well-being. In accordance with that, several recent reviews have called for an examination of the interrelationship of different mediators in the TFL–well-being relationship [1,10]. While a comprehensive simultaneous examination of job resources and job demands in their mediation potential of the TFL–employee well-being relationship is still lacking, the same is true for a differential analysis of their simultaneous influence on both positive and negative indicators of well-being. This calls for further investigation [9,10]. Additionally, research has mostly examined job resources as mediators, leaving the role of job demands widely unexplored [5,10].

In this study, we aim to answer these research calls by examining the relative mediating potential of various types of job resources (personal, relational, task-related and organizational) and job demands (challenge and hindrance demands) in the TFL–well-being relationship in a meta-analytical, multilevel investigation. We used the Job Demands–Resources Model (JD–R Model, [11]) as the theoretical framework for our model; however, we extended it. We (1) added the leadership variable as a predictor of work characteristics [9,12], (2) integrated different types of job demands and job resources and (3) differentiated among four indicators of employee well-being (affective–motivational, pleased–relaxed, depressed–exhaustive and irritated–distressed).

By integrating several job demands and job resources and various indicators of well-being in one model, we advance the disclosure of the mechanisms by which TFL unfolds and provide insights into the relative importance of various work characteristics for the relationship between TFL and different facets of well-being. Our study thereby contributes beyond recent reviews and meta-analyses examining the TFL–well-being relationship [1,10,13,14] and provides valuable information for future research and practice. It becomes clearer which resources and demands to focus on and implement and which ones to dispose of when aiming to impact employee well-being in the context of leadership. In doing so, we give organizations the opportunity to develop effective interventions in terms of shaping work characteristics for employees through the leader and maintaining or improving their well-being.

### 1.1. Transformational Leadership and Employee Well-Being

TFL [15] is the most widely researched leadership concept [16] and emphasizes change and personal development [17]. TFL is suggested to consist of four dimensions, namely idealized influence, inspirational motivation, intellectual stimulation, and individual consideration [15].

In recent years, the concept of TFL has received considerable conceptual and measurement critique, including a lack of theoretical grounding, insufficient specification of causal processes and highly interrelated subscales [18,19,20]. However, although refinement of the conceptual framework of TFL seems undoubtedly necessary, a recent meta-analysis by Hoch et al. [16] showed that only one other leadership concept (servant leadership) exhibited incremental variance over TFL for a few behavioral and attitudinal employee outcomes among various other leadership concepts (e.g., authentic and ethical leadership). Thus, TFL can still be regarded as a very influential and valuable leadership concept with high significance for employee outcomes.

Although TFL seems to have a predominantly positive effect on employees’ well-being at the workplace [2], a few studies also find evidence for an exhaustive component of this leadership style (e.g., [21,22,23]). While most TFL behaviors pertain to the followers’ needs and support the individual, there seem to be some elements of TFL that can appear overly challenging for employees (i.e., fostering growth, encouraging new ways of thinking, and welcoming the unknown) [21,24]. This may lead employees to burden themselves with an excessive number of job demands or may reduce their job resources, in turn hampering their well-being [21,22]. To shed light on the mechanisms by which TFL leaders convey their impact, we focus on this leadership concept in our analysis.

Well-being is a multifaceted construct with many existing conceptualizations of different facets [25,26]. We applied the taxonomy suggested by Salanova et al. [27], which is based on the circumplex model of well-being [28,29] and differentiates between four indicators of well-being: enthusiastic, relaxed, fatigued and tense. In our study, we adopted this taxonomy but renamed the different indicators to be more descriptive about their underlying subcomponents: we integrated affective–motivational well-being (formerly ‘enthusiastic’, e.g., work engagement, enthusiasm) and pleased–relaxed well-being (formerly ‘relaxed’, e.g., content, calm, relaxed) as two indicators of positive well-being and depressed–exhausted well-being (formerly ‘fatigued’, e.g., depression, burnout, depersonalization) and irritated–distressed well-being (formerly ‘tense’, e.g., stressed, irritated) as two indicators of negative well-being. 

Theoretically, TFL should contribute to employee well-being by enhancing personal growth and self-esteem through motivational processes inspired by TFL behaviors, such as emphasizing collective identity, referencing followers’ worth and efficacy, expressing confidence in the team and providing ideological visions [30,31,32]. Additionally, TFL leaders provide support and individualized coaching through their actions, thereby decreasing emotional distress and preventing negative mental health states [24,33,34]. Since theory and a compelling amount of evidence examining unidimensional TFL indicate a positive relationship between TFL and employee well-being, we expect the following:

**Hypothesis** **1** **(H1).** *TFL is positively related to affective–motivational well-being (1a) and pleased–relaxed well-being (1b) and negatively related to depressed–exhaustive well-being (1c) and irritated–distressed well-being (1d)*.

This hypothesis focuses on the direct influence of TFL on employee well-being. However, there are multiple ways by which leaders influence the work environment of employees (e.g., by assigning certain tasks), which, in turn, influence employee well-being. In our research, we thus applied a modified version of the JD–R Model as our theoretical framework: while the original JD–R Model subsumes leadership with other work-related resources under the category “job resources” (e.g., [35,36]), we believe that leaders have a particularly strong influence on the work characteristics of their employees and contribute to a favorably perceived work environment [12,37,38]. To investigate the indirect influence of leadership on employee well-being, we amplified the JD–R Model and suggest leadership as a prerequisite to the JD–R Model, thus examining TFL as an upstream variable in the model (see also [9,12]).

### 1.2. The Job Resources Model

According to the JD–R Model, job resources refer to those “aspects of the job that may do any of the following: (a) be functional in achieving work goals, (b) reduce job demands and the associated physiological and psychological costs, (c) stimulate personal growth and development” [11] (p. 501). As stated by the IGLO framework of Nielsen et al. [39], job resources can be differentiated into different categories: Individual, Group, Leadership and Organizational resources. We oriented ourselves to this framework and integrated individual (e.g., psychological capital, empowerment), group (e.g., social support of colleagues, community) and organizational resources (e.g., fairness perceptions of the organization, organizational support) in our analyses, renaming individual and group resources as personal and relational resources. We decided to drop the ‘leadership resources’ category suggested in the framework because these resources were conceptually and empirically very closely related to TFL (r = 0.57) and were expected to be confounded with TFL. In contrast to the IGLO framework, we did, however, add an additional category of task-related resources because we wanted to differentiate between job resources relevant to the individual workplace and task in a narrower sense (e.g., autonomy, job control, meaningful work) and job resources pertaining to the organization and the work environment in a broader sense (e.g., fairness, organizational culture, organizational support).

The TFL style entails a multitude of leadership behaviors that promote various types of employee job resources [40]. For example, by intellectually stimulating their employees, assigning tasks based on the employee’s strengths and capabilities and inspiring “out-of-the-box thinking”, TFL leaders encourage employees to create new paths, thereby promoting personal resources, such as occupational self-efficacy and empowerment [5,41]. By deciding on the degree of autonomy, predictability and control employees have over the fulfillment of their work tasks, TFL leaders greatly influence their employees’ task-related resources [1,42]. Moreover, they provide an impression of optimism and workplace safety for their employees through clear and transparent (one-on-one) communication and consideration, thereby enhancing collective identity, social support, and a common goal (relational resources) [43,44,45]. Beyond that, they foster perceptions of fairness and support regarding the organization (organizational resources) [46,47,48,49]. To summarize, employees should benefit from a TFL leader by acquiring work-related resources, and, thus, a positive relationship between TFL and job resources is expected.

Furthermore, we expect job resources to be positively related to indicators of employees’ positive well-being and negatively related to indicators of negative well-being. The motivational process of the JD–R Model describes a process through which job resources increase work engagement and foster personal growth (e.g., [50,51,52]). Other facets of positive well-being can be similarly expected to be positively influenced by job resources, as recently confirmed in a meta-analysis by Nielsen et al. [39]. In the same way job resources are positively associated with positive well-being, their absence can result in disengagement and burnout [50,53]. Thus, we expect a negative relationship between job resources and indicators of negative well-being.

**Figure 1:**
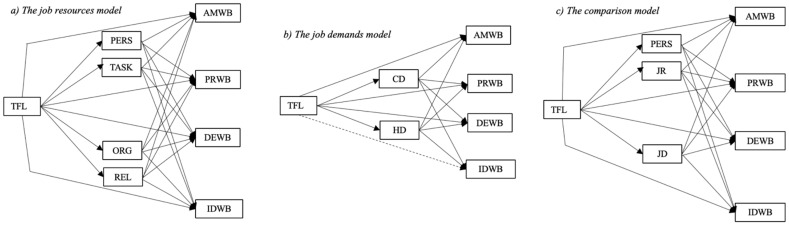
(**a**–**c**) The suggested models. Note: TFL = transformational leadership, PERS = personal resources, TASK = task-related resources, ORG = organizational resources, REL = relational resources, AMWB = affective–motivational well-being, PRWB = pleased–relaxed well-being, DEWB = depressed–exhausted well-being, IDWB = irritated–distressed well-being, JD = job demands, JR = job resources, CD = challenge demands, HD = hindrance demands.

According to the Conservation of Resources Theory (COR; [54]), individuals with the highest resource reservoir can best protect their well-being against harm and acquire new resources [55]. Because we assume that TFL leaders provide numerous resources for their employees, followers led by such leaders should have a substantial resource reservoir, engendering positive well-being and protecting them against negative well-being. Therefore, it is reasonable to assume that TFL leaders have a positive effect on indicators of positive well-being (e.g., [49,56,57]) and a protective effect against negative ones (e.g., [58,59,60]) by enhancing employees’ job resources (Figure 1a).

**Hypothesis** **2** **(H2).** *The relationship between TFL and employee well-being is mediated by personal (2a), task-related (2b), relational (2c) and organizational resources (2d) (the Job Resources Model).*

### 1.3. The Job Demands Model

In addition to enhancing job resources, TFL behaviors have been found to reduce job demands, which are important psychosocial risk factors for employees (e.g., [61,62]). However, the picture seems to be slightly more nuanced here, and research has identified different relational patterns with different job demands. According to the challenge–hindrance–stressor framework [63], job demands can be differentiated into two categories: challenge demands and hindrance demands. Challenge demands can be regarded as demands that, although stressful, are “rewarding work experiences well worth the discomfort” [63] (p. 66). Hindrance demands “involve excessive or undesirable constraints that […] hinder an individual’s ability to achieve valued goals” [63] (p. 67). TFL leaders prevent or reduce hindrance demands, such as role-related or relational conflicts, by articulating clear and concise (shared) goals and visions, transparent communication, and efforts for collaboration [64,65,66]. However, their high performance expectations and articulation of ambitious visions can also lead employees to increase their efforts, resulting in more challenge demands, such as time pressure or work overload [23,67]. Thus, we would expect TFL to be positively related to challenge demands and negatively related to hindrance demands.

Job demands also show a nuanced relationship with well-being. Intuitively, all job demands require energy and deplete a person and thus are positively linked to various indicators of negative well-being (e.g., anxiety, depression, and burnout) [68]. This relationship has also been identified in the health-impairment process of the JD–R Model [69] and has been demonstrated empirically many times (e.g., [50,70,71]).

The relationship between job demands and indicators of positive well-being is less clear. On the one hand, to our knowledge, work engagement is the only indicator of positive well-being tested in this context (for an overview, see [72]). On the other hand, the findings regarding this indicator are inconsistent. Part of the reason for this inconsistency might be that authors have failed to differentiate the job demands measured in challenge and hindrance demands [73]. Hindrance demands have been shown to reduce well-being and hamper motivational states [72,74], leading to a greater focus on weaknesses and negative aspects of work [75] and thereby attenuating feelings of positive well-being. We, therefore, expect a negative relationship between hindrance demands and indicators of positive well-being. Challenge demands, in contrast to hindrance demands, are said to enhance motivation and feelings of positive well-being by satisfying psychological needs [72,76,77]. Thus, we expect a positive relationship between challenge demands and indicators of positive well-being. On the whole, we assume TFL leaders shape their employees’ work characteristics by reducing (hindering) job demands and providing challenges as described above, subsequently reducing stress and other indicators of negative well-being and fostering positive well-being (see Figure 1b) [5,9,78].

**Hypothesis** **3** **(H3).** *The relationship between TFL and employee well-being is mediated by challenge (3a) and hindrance demands (3b) (the Job Demands Model).*

### 1.4. The Relative Impact of Job Resources and Job Demands

While individual job resources and job demands have been tested as mediators in the TFL–well-being relationship, studies examining several work characteristics at the same time are still rare [1]. Only a handful of studies have investigated job resources and job demands in the TFL–well-being relationship [5,9,79,80]. Unfortunately, however, those studies only examined mediation effects regarding the relationship between TFL and indicators of negative well-being (i.e., stress, anxiety, irritation), leaving the differential effects of job resources and job demands on the relationship between TFL and positive well-being completely unexplored. Furthermore, to our knowledge, no study has compared the mediation potential of several types of resources and demands, and, thus, their relative importance in the TFL–well-being relationship cannot be decided. We intended to fill these research gaps in two consecutive steps: first, we examined the relationship between TFL and all the indicators of well-being regarding the relative mediating impact of job resources and job demands as well as personal resources (e.g., occupational self-efficacy, empowerment, psychological capital), which we contrasted in a separate category (see Figure 1c). We refrained from subsuming personal resources under job resources in this model because, even though they are job-related, these resources have a strong individual component, which we wanted to contrast to other types of resources that are more in the scope of leadership.

**Figure 2:**
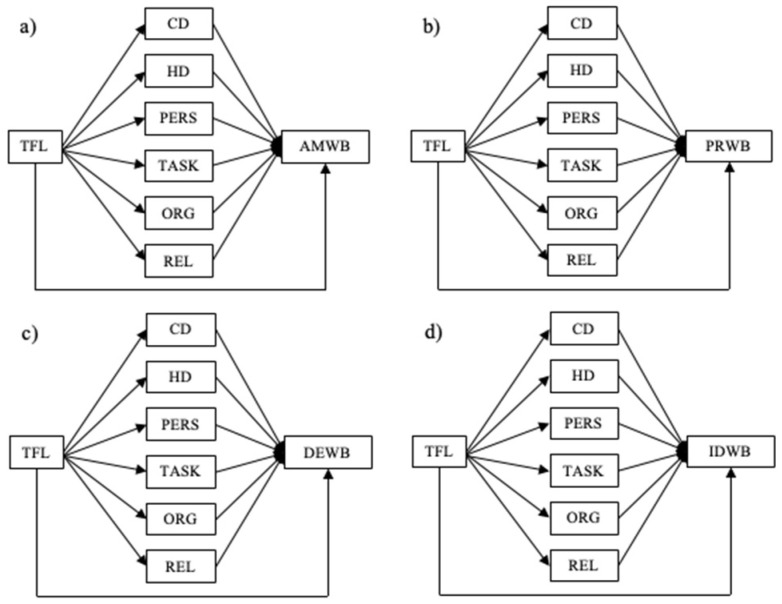
(**a**–**d**) Suggested mediating mechanisms of all types of job resources and demands in the relationship between TFL and one indicator of well-being at a time. Note: TFL = transformational leadership, CD = challenge demands, HD = hindrance demands, PERS = personal resources, TASK = task-related resources, ORG = organizational resources, REL = relational resources, AMWB = affective–motivational well-being, PRWB = pleased–relaxed well-being, DEWB = depressed–exhausted well-being, IDWB = irritated–distressed well-being.

In a second step, we compared all the examined types of resources and demands in terms of their potential to mediate between TFL and affective–motivational well-being, pleased–relaxed well-being, depressed–exhaustive well-being and irritated–distressed well-being, respectively (Figure 2a–d). This led us to the following research questions:

**Research** **Question** **1:** *What is the relative mediating effect of personal resources, job resources and job demands on the TFL–well-being relationship? (the Comparison Model)*

**Research** **Question** **2:** *What is the relative mediating effect of each type of resource and demand on the relationship between TFL and different indicators of well-being?*

## 2. Materials and Methods

### 2.1. Literature Search and Eligibility Criteria

To find all relevant studies in the abovementioned field, an extensive search of the literature through May 2020 was conducted. Various electronic databases, search engines and the internet were searched for various terms related to transformational leadership and well-being, burnout, work engagement, mental health, and psychological complaints (e.g., PsychINFO, MEDLINE, PsychArticles, The Cochrane Library, Academic Search Premier, Google Scholar). After these electronic searches, we also searched reference lists of relevant articles and contacted various authors of important works on TFL to ask for additional and/or unpublished literature. A detailed search history can be found in the Supplementary Materials (Table S1).

**Figure 3:**
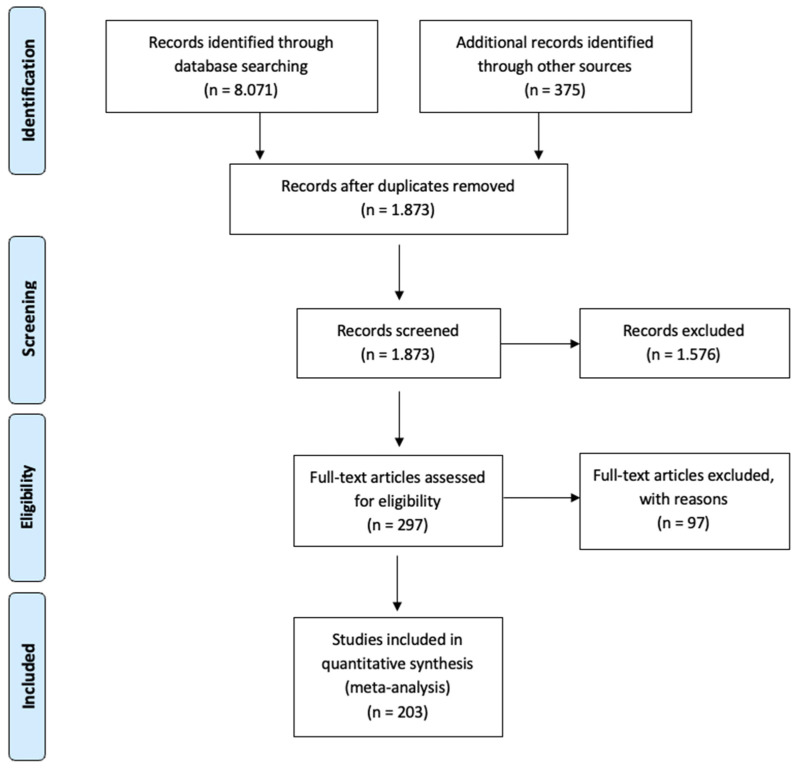
Flow chart of literature inclusion.

The identified works were screened based on the following eligibility criteria: studies had to (1) be an empirical field study, (2) measure TFL and an indicator of positive or negative well-being, (3) include participants who worked at least 20 h per week, (4) measure TFL from the perspective of followers and (5) include all necessary information for calculations. Applying these inclusion criteria, 203 studies with 243 samples were included in this meta-analytic investigation (*N* = 195.064). Figure 3 displays the search process. A Prisma checklist can be found in Table S2.

### 2.2. Coding Procedure and Included Variables

Two independently working psychological researchers extracted the relevant data from the studies with an interrater agreement of 99.97%. All disagreements or uncertainties were discussed and resolved by consensus. When only subscale scores of variables were reported, we calculated mean scores across these subscales. A coding manual can be viewed in Table S3.

All variables that were examined in this study were cross-sectional and rated from the followers’ perspective. Next to sample characteristics (e.g., industry, continent, gender) we coded leadership measurements, well-being measurements and the measurements of work characteristics. Study quality was assessed by the impact factor of the journal the primary study was published in.

#### 2.2.1. Transformational Leadership

Measurements had to reflect the core dimensions of TFL [15]. According to van Knippenberg and Sitkin [19], the subscales of existing measurements of TFL overlap substantially, so we decided on one overall score for TFL. Further analysis of the subscales was also not possible because only 24 of 243 samples (10%) reported individual dimensions. TFL was mostly measured by the Multifactor-Leadership Questionnaire (MLQ) [81] (62%) but also by the Global Transformational Leadership Scale (GTL) [82] (13%), the Transformational Leadership Inventory (TLI) [83] (9%), the Transformational Leadership Scale (TLS) [65] (7%) and other measurements of TFL (10%). We tested in moderator analyses whether it is reasonable to treat all instruments equally. Since the instruments did not vary substantially in their relationships with the other variables, we decided not to differentiate between them (see also Section 3.4). 

#### 2.2.2. Indicators of Well-Being

As described above, we differentiated the construct of well-being into four indicators of well-being. To supply the reader with even more detail, we measured subcomponents of the indicators wherever possible and sensible.

Since the leader–employee relationship is a rather volatile one [84], we regard it as rather unlikely that leaders have influence on very general and trait-like well-being (i.e., confidence with life). Thus, in accordance with our research question, we examined domain-specific (i.e., job-related) state well-being [85], excluding studies that used very trait-like measurements (i.e., “in general”, “regarding my life”). Note that we also refrained from integrating job satisfaction as an indicator of well-being because correlations between job satisfaction and leadership are often artificially high (e.g., ρ = 0.58, [86]) due to confounding of the two concepts in items of job satisfaction scales (e.g., “How satisfied are you with your immediate boss?”, Job Satisfaction Scale, [87]). Because this circumstance would have artificially increased the correlations of leadership and employee well-being in our meta-analysis, we chose to exclude job satisfaction.

##### Indicators of Positive Well-Being

*Affective–motivational well-being.* Positive affective states describe feelings of being “enthusiastic, active and alert” [88] (p. 1063). Thus, constructs such as positive affect, energy, enthusiasm, flow, alertness, thriving and activity were elements of this indicator. A widely used element of this indicator was work engagement. Work engagement can be defined as a “positive affective–motivational state of fulfillment that is characterized by vigor, dedication and absorption” [89]. Examples of instruments used for this indicator are the UWES [89], the PANAS [88] and the JAWS [90]. Positive affect and work engagement constitute subcomponents of this indicator. The alpha values were α = 0.71–0.98 for the whole indicator and α = 0.71–0.98 and α = 0.82–0.97 for the subcomponents work engagement and positive affect, respectively.

*Pleased–relaxed well-being.* This indicator describes a content and relaxed state in which one feels rested and confident about one’s own skills [27]. Examples of this indicator are (subjective) well-being measured by the WHO-5 [91] or the GHQ [92] (α = 0.67–0.98).

##### Indicators of Negative Well-Being

*Depressed–exhausted well-being.* Part of this indicator is depressed states as well as the concept of burnout. Burnout is defined as a syndrome of “energy depletion and dysfunctional attitudes toward the workplace” [93] (p. 4). It is typically represented by three dimensions: emotional exhaustion, depersonalization and reduced personal accomplishment. Whenever possible, we entered all three subscales separately to obtain as much information as possible. Instrument examples are the MBI [94], the GNBI [95] and the burnout subscale of the COPSOQ [96]. Subcomponents of this indicator were emotional exhaustion (α = 0.70–0.98), depersonalization (α = 0.70–0.98), reduced personal accomplishment (α = 0.70–0.98), depression (α = 0.87–0.96) and burnout (when measured as one dimension) (α = 0.80–0.97). The alpha for the whole indicator was α = 0.70–0.98.

*Irritated–distressed well-being.* This indicator describes anxious, tense and/or angry states mainly triggered by the job [27]. Subcomponents were job stress; negative affect, which describes “aversive mood states like anger, contempt, disgust, guilt, fear and nervousness” [88] (p. 1063) and irritation, which refers to “a state of mental impairment resulting from a perceived goal discrepancy” [97] (p. 198). Instrument examples used for this indicator are the PANAS [88], the DASS [98] and the PSS [99]. The following alpha values were obtained: the whole indicator (α = 0.80–0.97), irritation (α = 0.82–0.97), job stress (α = 0.80–0.97) and negative affect (α = 0.87–0.97).

#### 2.2.3. Work Characteristics

As mediators, we integrated various types of job resources and job demands in the analyses to cover the characteristics of a workplace most completely. We integrated work characteristics only when they were reported in conjunction with TFL and indicators of well-being in one study. We did not examine additional studies focusing on work characteristics in other contexts to avoid introducing more heterogeneity into the analyses.

*Job resources.* Job resources are work features that stimulate personal growth and achievement [39]. In accordance with the IGLO framework [39], we differentiated personal, relational, and organizational resources and added the category task-related resources. Personal resources subsumed all measurements that pertained to individual development at the workplace and included resources such as self-efficacy, empowerment, innovative behavior, psychological capital, intrinsic motivation, professional ambition, and competence. Instrument examples are the Occupational Self-Efficacy Scale [100], which was used quite often in this category. Task-related resources concern the conditions of the work tasks and comprise, for example, job control, autonomy, predictability, clarity, and meaningfulness, which were often represented by various subscales of the COPSOQ [96]. Relational resources described social relationships and comprised resources such as social support, community, cooperation, cohesion, social interaction, and teamwork and were represented, for example, by a social support scale by de Jonge et al. [101]. Organizational resources pertained to the perceptions of organizational core values and comprised variables such as fairness, values, justice, organizational support, structural empowerment and climate, represented by, for example, the fairness and value subscales of the Areas Of Worklife Survey [102]. Alpha values were as follows: personal (α = 0.69–0.98), task-related (α = 0.59–0.95), relational (α = 0.69–0.93) and organizational resources (α = 0.66–0.96).

*Job demands.* Job demands refer to aspects of work that require effort of some type and are, therefore, linked to psychological or physical costs or limitations [69]. We differentiated between challenge demands and hindrance demands [77]. Challenge demands comprised, for example, workload, work intensity and time pressure, with an instrument example of a subscale of the ISTA [103]. Hindrance demands were understood as, for example, role conflicts, role ambiguity, bullying and emotional demands as measured by the Role Conflict and Ambiguity Scale [104]. Alphas were as follows: challenge demands (α = 0.56–0.90) and hindrance demands (α = 0.47–0.95).

A detailed description on each integrated study can be viewed in Table S4.

### 2.3. Meta-Analytic Approach

Statistical analyses were computed with the statistical freeware R [105] and the packages metafor [106], metaSEM [107], lavaan [108] and msemtools [109]. The R Markdowns can be viewed at https://osf.io/c59q2/ (accessed on 26 January 2022).

#### 2.3.1. Meta-Analyses

All studies reported correlation coefficients or standardized beta-coefficients as effect sizes. In the first step, estimates were corrected for measurement error by a double-attenuation correction of the estimates by reliability scores given in the individual studies [110]. Where no reliability score was provided, we applied an alpha value of α = 0.90, which can be regarded as conservative since it is unlikely to change the original values. After the analyses, estimates were converted back to correlation coefficients.

To test our proposed research models, we first conducted individual meta-analyses for all relevant individual bivariate relationships and several subcomponents of well-being by calculating three-level random-effects meta-analyses with the meta3 function of the metaSEM approach of Cheung [111]. This approach models the sampling variation of the effect sizes at Level 1, the variation within studies at Level 2 and the variation between studies at Level 3 [111]. We thereby accounted for the dependencies of several effect sizes per well-being outcome obtained from the same sample and, thus, the multilevel structure of our data [112,113]. To weigh the effect sizes, we employed the inverse of the within-study sampling variance [114]. We used correlations as effect sizes in the meta-analyses instead of Fisher’s z values for consistency with the meta-analytical structural equation modeling that followed in a next step, which required the use of the correlational metric and the associated variances and covariances [115,116].

We checked for outliers via boxplots and excluded 106 of 2501 (4%) correlations that were identified as outside the whiskers of the boxplot in a second dataset. Since the results of the calculations with this second dataset do not differ significantly from the original ones with all effect sizes, we report the results with the complete dataset in this article. The analyses without outliers can be viewed in R Markdown (accessed on 26 January 2022).

#### 2.3.2. Meta-Analytic Structural Equation Modeling

After performing individual meta-analyses, we applied meta-analytic structural equation modeling (MASEM) to estimate the theoretically suggested mediating mechanisms [115]. The advantage of the TSSEM (two-stage structural equation modeling) approach of Cheung, compared with univariate approaches, is that it pools the individual sample correlation matrices and then computes a pooled correlation matrix with a corresponding sampling variance–covariance matrix. Thus, it integrates more information and increases the validity of the proposed relationship estimates [115]. Moreover, it does not depend on finding a common sample size for the combined correlations (e.g., harmonic mean) and results in a reduced confirmation bias compared with structural equation modeling of primary data [115].

Given the multilevel structure of our data, we did not apply the first step of the TSSEM approach suggested by Cheung [115] but instead applied a multilevel approach by Wilson et al. [116]. This prevents the underestimation of the variances of dependent effect sizes [116]. Wilson’s approach first fits a three-level random-effects model, which, apart from small numerical differences, entails the meta-analytic correlations calculated in the three-level meta-analyses described above. The pooled correlation matrix is then created based on these correlations. In a subsequent step, this pooled correlation matrix is handed over to the second step of the TSSEM approach to estimate the SEM using weighed least squares (WLS) estimation.

#### 2.3.3. Moderator Analyses

Three-level meta-analyses provide the opportunity to inspect several heterogeneity statistics. Due to the differentiation of three levels, besides a Q-statistic, the amounts of variation in Level 2 (*τ*_(2)_^2^) and in Level 3 (*τ*_(3)_^2^) are given next to respective proportions of total variation within (*I*^2^_(2)_) and between studies (*I*^2^_(3)_). According to Cleophas and Zwinderman [117], an *I*^2^_(3)_ above 0.50 indicates high heterogeneity. Thus, for between-study heterogeneity above 50%, we inspected several moderators exploratorily at the study level: study quality, publication status, year of publication, continent of the sample, industry of the sample and the measurement of TFL. The analyses were conducted with the R package msemtools [109].

#### 2.3.4. Publication Bias

Several measures were taken to test for publication bias. The relevant analyses can be viewed in File S1. The visual inspection of funnel plots [118] and the application of Egger’s test [119] indicated some missing studies for the relationships of TFL with positive affect and emotional exhaustion, whereby Egger’s tests were only significant at the 90% threshold. We subsequently applied trim and fill analyses [120] to simplified versions of our models (two- instead of three-level meta-analyses) due to a missing equivalent method for three-level data. They revealed two missing studies for positive affect. However, the null hypothesis of no missing studies could not be rejected. The trim and fill for emotional exhaustion did not suggest any additional studies. Thus, the influence of publication bias can be regarded as neglectable. All other funnel plots and Egger’s tests can be viewed in R Markdown.

## 3. Results

### 3.1. Meta-Analytic Correlations between the Variables

The analyses included 2501 correlations between TFL, work characteristics and different indicators of well-being across 53 individual three-level meta-analyses. The interested reader finds all three-level meta-analyses on TFL and the indicators of well-being, including various subcomponents of well-being, and the ones of the mediators and TFL and well-being in Appendix A.

To sum up the results, TFL was significantly and substantially associated with all indicators of positive and negative well-being; thus, we focus on those relationships that are relevant for our hypotheses. The strongest positive relationship was found for TFL and affective–motivational well-being (*r* = 0.39), while it was only slightly lower for TFL and pleased–relaxed well-being (*r* = 0.34). This confirms H1a and H1b. TFL was negatively associated with depressed–exhaustive well-being (*r* = −0.28), irritated–distressed well-being (*r* = −0.20) and psychosomatic complaints (*r* = −0.21), confirming H1c, H1d and H1e. Forest plots regarding these meta-analyses can be viewed at [1] (accessed on 26 January 2022).

Regarding the meta-analyses on the mediators and TFL and well-being, except for one exception, the relationships were all in the expected direction as described in the theoretical background. The relationships of challenge demands with TFL and the indicators of positive well-being, which were expected to be positive, were found to be negative. All the explored relationships were significant and of small to moderate size. TFL was negatively associated with job demands (challenge: *r* = −0.22; hindrance: *r* = −0.29), while it was positively associated with all job resources (organizational: *r* = 0.51, relational: *r* = 0.40, task-related: *r* = 0.38, personal: *r* = 0.27).

### 3.2. Structural Equation Models

To test our hypotheses and research questions, we analyzed several meta-analytical SEMs. The full correlation matrices used for the SEM can be found in the Supplementary Materials (Table S5).

**Figure 4:**
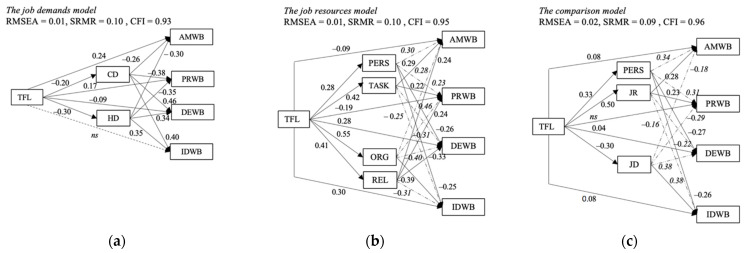
(**a**–**c**) Parameter estimates for the mediation effect of job resources, job demands and the Comparison Model on the TFL–well-being relationship. Note: all effect sizes are significant at α = 0.05; for better readability of the estimate values, dotted paths correspond to values in italics; abbreviations of the variable names = see Figure 1a–c.

The Job Resources Model tested the mediation effect of personal resources (H2a), task-related resources (H2b), organizational resources (H2c) and relational resources (H2d) on the TFL–well-being relationship. Figure 4a shows the parameter estimates of this model, while Table 1 shows the indirect effects. The fit indices of the model indicated good fit (RMSEA = 0.01, SRMR = 0.10, CFI = 0.95). TFL was positively related to all job resources (personal resources: *β* = 0.30, task-related resources: *β* = 0.42, organizational resources: *β* = 0.56, relational resources: *β* = 0.43), while these were, in turn, positively related to the indicators of positive well-being (*β* = 0.22–0.47) and negatively related to the indicators of negative well-being (*β* = −0.25–−0.40). The indirect effects were all significant, with the weakest indirect effects for personal resources (*β_i_* = 0.07–0.09) and the strongest ones for organizational resources (*β_i_* = 0.17–0.26) (see Table 1). Thus, H2a–d could be confirmed.

The Job Demands Model tested the mediation effect of challenge demands (H3a) and hindrance demands (H3b) on four indicators of well-being. Figure 4b shows the parameter estimates of this model, while Table 1 displays the indirect effects. The model had an acceptable fit (RMSEA = 0.02, SRMR = 0.10, CFI = 0.93). TFL was negatively related to challenge demands (*β* = −0.23) and hindrance demands (*β* = −0.30), while these were, in turn, related to the indicators of well-being. Challenge demands were more strongly related to the negative indicators (depressed–exhaustive well-being: *β* = 0.47, irritated–distressed well-being: *β* = 0.39) than to the positive indicators (affective–motivational well-being: *β* = −0.26, pleased–relaxed well-being: *β* = −0.33), while this pattern was less pronounced for hindrance demands (affective–motivational well-being: *β* = −0.28, pleased–relaxed well-being: *β* = −0.33, depressed–exhaustive well-being: *β* = 0.35, irritated–distressed well-being: *β* = 0.37). The indirect effects were all significant (*β* = 0.06–0.11), which indicates that job demands mediated the relationships between TFL and all the indicators of employee well-being. Thus, H3a and H3b were confirmed. Moreover, the direct effects of TFL on the indicators of negative well-being were small (*β* = −0.08, (−0.12, −0.04) for depressed–exhaustive well-being) to very small in size (*β* = −0.01, (−0.06, 0.03) for irritated–distressed well-being) and, in the case of irritated–distressed well-being, even nonsignificant, which indicates a very large impact of job demands on indicators of negative well-being.

### 3.3. The Relative Strength of Mediating Effects of Work Characteristics

To judge the relative influence of the different job demands and resources on individual indicators of well-being (RQs 1 and 2), we first tested a model comparing the mediating potential of the broader categories of personal resources, job resources and job demands in the TFL–well-being relationship (the Comparison Model, see Figure 1c). Subsequently, it would have been the goal to test all the work characteristics and indicators of well-being in one model. However, we could not manage to get this model to converge, which is why we cannot trust its results and do not present it in the manuscript. Instead, to analyze the relative mediating effect of all the work characteristics in the TFL–well-being relationship, we calculated four models entailing all the work characteristics and one indicator of well-being each (see Figure 2a–d). First, we report the results of the Comparison Model and complement those by additional relevant results of the models entailing all work characteristics and one indicator of well-being each.

As displayed in Figure 4c, the Comparison Model indicated a good fit (RMSEA = 0.02, SRMR = 0.09, CFI = 0.95). While TFL was positively related to personal and job resources (personal: *β* = 0.35, job resources: *β* = 0.51), it was negatively related to job demands (*β* = −0.31). All the resources were, in turn, positively related to indicators of positive well-being (personal resources: *β* = 0.36, job resources: *β* = 0.30 to affective–motivational well-being and personal resources: *β* = 0.32, job resources: *β* = 0.24 to pleased–relaxed well-being) and negatively related to indicators of negative well-being (personal resources: *β* = −0.27, job resources: *β* = −0.22 to depressed–exhaustive well-being and personal resources: *β* = −0.26, job resources: *β* = −0.17 to irritated–distressed well-being). Job demands were negatively related to indicators of positive well-being (*β* = −0.16 to affective–motivational well-being, *β* = −0.26 to pleased–relaxed well-being) and positively related to indicators of negative well-being (*β* = 0.39 to depressed–exhaustive well-being, *β* = 0.40 to irritated–distressed well-being).

The indirect effects of the Comparison Model are displayed in Table 1. All the indirect effects of the model were significant and of small to moderate size, while the direct effects of TFL on the indicators of well-being were small (*β* = 0.06 for affective–motivational well-being, *β* = 0.07 for depressed–exhaustive well-being, *β* = 0.11 for irritated–distressed well-being), and in the case of pleased–relaxed well-being, even very small and nonsignificant (*β* = 0.03, [−0.04, 0.11]). This indicates mediation of the TFL–well-being relationship through personal resources, job resources and job demands for all the indicators of well-being.

When inspecting the relative impact of personal resources, job resources and job demands on the TFL–well-being relationship, one sees that personal resources and JR had a stronger effect on the relationship between TFL and the indicators of positive well-being than JD (*β* = 0.12, *β* = 0.15 for affective–motivational well-being compared to *β* = 0.05; *β* = 0.12, *β* = 0.11 for pleased–relaxed well-being compared to *β* = 0.08, respectively). This pattern was reversed for the relationship between TFL and the indicators of negative well-being (*β* = −0.09, *β* = −0.11, *β* = −0.12 for depressed–exhaustive well-being; *β* = −0.09, *β* = −0.08, *β* = −0.13 for irritated–distressed well-being, respectively).

**Figure 5:**
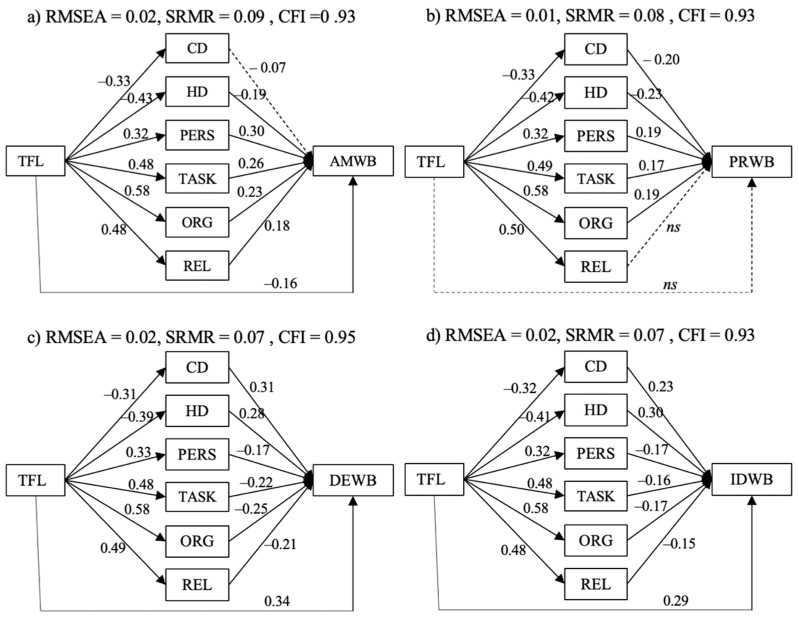
(**a**–**d**) Parameter estimates for the models including all types of work characteristics and one well-being indicator. Note: all effect sizes are significant at α = 0.05; ns = nonsignificant; abbreviations of the variable names = see Figure 2a–d.

RQ2 asked for the relative influence of all the types of work characteristics on individual indicators of well-being. Figure 5a–d shows the direct effects of these analyses, while Table 2 displays the indirect effects. All the models displayed in Figure 5a–d had an acceptable fit.

All the models showed significant direct and indirect effects, except for two paths. First, the relationship of challenge demands with affective–motivational well-being was nonsignificant (*β* = −0.05, (−0.12, 0.02)). The mediation effect of challenge demands on the relationship between TFL and affective–motivational well-being was, thus, also weak and nonsignificant (*β_i_* = −0.02, (−0.01, 0.04)). Second, the relational resources/pleased–relaxed well-being relationship was marginally nonsignificant (*β* = 0.13, (−0.00, 0.25)), which also resulted in a nonsignificant indirect effect of REL on the TFL/pleased–relaxed well-being relationship (*β_i_* = 0.06, (−0.00, 0.12)).

Over all four models, job resources were most strongly related to affective–motivational well-being (*β* = 0.32–0.20 for affective–motivational well-being, *β* = 0.25–0.13 for all other indicators of well-being), while job demands were most strongly related to indicators of negative well-being (*β* = 0.33–0.22 for the negative indicators, *β* = −0.23–−0.05 for the positive indicators). Organizational resources had the strongest mediating effect on the relationships between TFL and all the indicators of well-being. The weakest mediating effects could be found for job demands on the relationship between TFL and affective–motivational well-being, for challenge demands on the relationship between TFL and pleased–relaxed well-being and for personal resources on the relationships between TFL and all indicators of well-being, except affective–motivational well-being. Since the direct effect of TFL on pleased–relaxed well-being was nonsignificant (*β* = −0.12, (−29, 0.05)), the relationship between TFL and pleased–relaxed well-being was fully mediated by the work characteristics challenge demands, hindrance demands, personal resources, task-related resources, and organizational resources but not, however, by relational resources. 

### 3.4. Moderator Analyses

When the between-study heterogeneity in the relationship of TFL and employee well-being exceeded 50% (see Appendix A), we conducted moderator analyses with various categorical study-level moderators (study quality, publication status, publication year, continent of sample, industry of sample and kind of TFL measure). However, the moderator analyses provided rather inconsistent results (e.g., significance of moderators depending on the level of aggregation of the well-being indicators) and explained only very limited proportions of heterogeneity so that we were not entirely confident in interpreting them and refrained from reporting them here. The interested reader can find a supplement reporting on the most relevant results and on a judgement of the study quality of the primary studies in the Supplemental Material (File S2). Additionally, the complete analyses can be viewed in the R Markdowns.

## 4. Discussion

Multiple mediators have been identified in the TFL–well-being relationship in previous research. However, these research findings have piled up to stand next to each other and do not give an indication as to which mediators are the most relevant ones in this relationship. This study intended to fill this research gap by synthesizing the existing evidence and examining the relative mediation impact of various types of job demands and resources with regard to TFL and its relationship with several indicators of well-being.

Overall, our study found all examined work characteristics (challenge and hindrance demands and personal, task-related, relational and organizational resources) to mediate the relationship between TFL and employee well-being, which hints at the possibility of job demands and job resources being helpful tools for leaders to influence their employees’ well-being at the workplace. While all work characteristics appeared relevant, organizational resources were identified as the strongest mediators in this study. Personal resources, on the other hand, had the weakest mediating effects, at least compared with those of the other job resources. These findings contribute important knowledge to the existing research in giving researchers and practitioners a first guideline on what to examine and how to proceed to enhance employee well-being through TFL [10,19]. Thus, the so far rather arbitrary choice of mediators can be replaced by an informed one. 

Confirming our first hypothesis, TFL was positively (and more strongly) related to the indicators of positive well-being (affective–motivational well-being and pleased–relaxed well-being) and negatively related to the negative ones (depressed–exhaustive well-being and irritated–distressed well-being). This pattern was evident for all the subcomponents of well-being. The strongest meta-analytic relationship was found for TFL and work engagement, which was also the most widely examined subcomponent of well-being in our analyses (23% of all correlations). These findings acknowledge the inspiring and motivating elements of TFL, which are important for developing high positive employee well-being and, particularly, work engagement [121,122].

Our second and third hypotheses asked for the mediating potential of job resources (H2) and job demands (H3) in the TFL–well-being relationship. As already stated, all the explored job resources and demands were relevant mediators, confirming the hypotheses. In more detail, job demands were the stronger mediators in the relationship between TFL and indicators of negative well-being, while job resources seemed to be more strongly mediating the TFL–positive well-being relationship (especially affective–motivational well-being). These findings confirm the observation by Inceoglu et al. [10] that positive and negative well-being are mediated in differing strengths and by different mediators and need to be differentiated when exploring the effect of TFL on well-being. Additionally, they underscore the motivational and health impairment processes suggested by the JD–R Model [69]: while job resources, such as autonomy or opportunities for development, enhance positive well-being states for employees, job demands deplete energy, leading to more negative well-being states [73].

The Job Demands Model in our study showed a strong mediation impact of job demands on the indicators of negative well-being, even fully mediating irritated–distressed well-being. It also showed that job demands play a role in the relationship between TFL and indicators of positive well-being, a path that has been under-researched so far [73]. Following the proposal of Breevaart and Bakker [73], to bring more light into this relationship by differentiating between challenge and hindrance demands, we found a slightly stronger negative mediation effect of hindrance demands (than challenge demands) on the TFL–positive well-being relationship. Interestingly, our expectation that certain (overtaxing) TFL behaviors promote challenge demands was not met. Instead, TFL reduced challenge demands. Thus, according to our findings, TFL leaders do not seem to overtax employees through their behaviors; on the contrary, they seem to be able to reduce challenging demands, such as excess workload, similar to hindrance demands. Our study could, therefore, not support the overtaxing elements of TFL. Instead, it seems that employees view their TFL leader as transformational so long as the leader shows all the positive attributes associated with a TFL leader. When overtaxing leadership behaviors are shown, those leaders might no longer be viewed as transformational.

Surprisingly, challenge demands also showed a negative relationship with indicators of positive well-being. This finding was also contrary to our expectations, underscoring the ambivalent nature of challenge demands [123] and calling for attention to the rest of the work environment in the face of challenge demands. For example, several research works found (personal) job resources to be necessary for employees to retain positive well-being in the face of challenge demands [22,73,74,123]. Moreover, appraisal processes should be taken into consideration. A challenge demand for one person might be a hindrance demand for another person [124]. In summary, TFL leaders seem to be able to reduce all kinds of job demands and thereby enhance the positive well-being of their employees. However, the ambivalent nature of challenge demands raises the question of other environmental factors (e.g., the number of simultaneous resources) or appraisal processes to be considered in this interplay. In any case, the link between job demands and indicators of positive well-being, especially differentiating challenge and hindrance demands, is one that should be integrated and further developed in future research considerations.

Comparing the relative impact of all the work characteristics (R1 and R2), TFL was most strongly associated with organizational resources, which were also identified as the strongest mediating work characteristics across all the analyses. This fits the very definition of TFL, which entails the elements of change and articulates an attractive (organizational) vision, thereby creating hope and optimism conducive to organizational growth [7,121]. Through their TFL behaviors, TFL leaders provide structural empowerment and a climate for innovation, motivating their followers to act beyond self-interest and embrace higher organizational purposes [125,126]. This enhances employees’ beliefs in themselves and satisfies their basic psychological needs, in turn creating enhanced employee well-being, especially affective–motivational well-being components such as work engagement [7,127]. Additionally, TFL leaders represent and transmit organizational core values and ethical standards [24,58], facilitate justice perceptions [47,128] and delegate responsibility [58], thereby enhancing their employees’ feelings of involvement, congruence and just treatment. Thus, at their core, TFL leaders represent, translate and channel organizational values, goals and rules and convey security, belonging and appraisal to their employees, thereby contributing to individual well-being.

Personal resources, on the other hand, had the weakest mediating effects on the relationship between TFL and all the indicators of well-being, except for affective–motivational well-being. It seems that personal resources, such as occupational self-efficacy or proactivity, although job-specific, are only influenced by the leader to a small degree and rely greatly on personal initiative and past experiences [121,129]. These findings imply that leaders would be most successful in enhancing personal resources by giving their followers room to develop themselves (e.g., by granting time for personal development and encouragement) and supporting organizational initiatives for personal coaching or training [22].

The models entailing all the work characteristics showed a full mediation of the relationship between TFL and pleased–relaxed well-being. Thus, it seems that, for employees to be calm, focused and content, leaders are measured only by their ability to create a pleasant work environment. Caring for an abundance of job resources and reducing job demands are the leaders’ role in this regard [127,130].

## 5. Limitations and Implications for Future Research

First and foremost, it must be noted that our findings are based on cross-sectional data and are, therefore, not conducive to inferences of causality and reciprocity. Although there is compelling theoretical reasoning to suggest this order of the studied variables, it would also be conceivable, for example, for work characteristics to influence how TFL leaders behave in response to ambient factors, in turn influencing the well-being of their employees [131,132]. Other longitudinal studies have proposed a reciprocal relationship between TFL and employee well-being, in which better well-being predicted better perceived leadership or in which both variables influenced one another [133,134]. Moreover, Inceoglu et al. [10] found some inconsistencies in the results of cross-sectional and longitudinal studies exploring the mediation effects between TFL and employee well-being. Thus, longitudinal research is necessary to establish the right ordering of the variables of leadership, job resources and job demands in addition to the aspects of positive and negative employee well-being and to confirm the cross-sectional results of this study. The same is true for the prevention of selection bias in that employees were chosen for certain TFL leaders based on certain characteristics. Adding to this call, due to the inevitable broadness of the work characteristic and well-being categories in this study, this piece should be regarded as a first prospect on the relative value of different work characteristic categories in the TFL–well-being relationship and should be complemented by narrower and more differentiated scopes of research questions. 

Second, the data in our study were based on self-report data, which poses the risk of inflated results due to a common method bias [135]. However, we decided to focus on this reporting style for the examined variables for various reasons. For example, TFL in its linkage to employee well-being can best be rated by followers. They are the ones interacting with and observing their leader at their workplace, while ratings by the leaders would have had a high risk of being biased in their favor. Furthermore, well-being is very subjective and is unlikely to be influenced by faking or other impression management issues that would threaten the validity of the measurements [136]. Moreover, self-reports provide qualitatively rich information about the conditions of a person who no one else has access to, and participants will most likely be very willing to provide information about their workplace or state of well-being and use this information to their advantage (e.g., to provoke change after a survey) (e.g., [137,138]). What is more, the research is not united regarding a methodology bias of self-report data [136,139]. Therefore, we considered self-reports to be a suitable measurement approach for the examined variables in our study.

Based on the very nature of meta-analyses, we were dependent on the data provided by the examined studies. Thus, our data consisted of studies from many different contexts, populations, etc., leading to substantial heterogeneity. To reduce the heterogeneity, we tried to find a good balance between types/indicators that were narrow enough to measure similar facets of well-being and work characteristics while also being broad enough to acquire a substantial number of correlations per type/indicator. Additionally, we conducted several moderator analyses of study-level moderators. Because these analyses did not yield consistent results over different levels of aggregation and did not explain a huge amount of variance, the question of potential moderators remains unanswered and warrants further research. The study of boundary conditions and contextual factors of the TFL–well-being relationship seems to be a promising one here [1] as, especially when regarding well-being, people might have different starting points and ways to adapt to external stimuli [22,140]. Unfortunately, it is not yet possible to implement such individual-level moderators in meta-analytic research so far, which is why we have to point to primary research to enhance knowledge in this area. For example, the study of personal resources as boundary conditions for how well employees can make use of the TFL behaviors of their leaders would advance the current research. Some previous research identified personality traits as an important leverage point for making use of the TFL behaviors of the leader [141,142,143]. Other research identified more malleable personal resources, such as detachment from work, as important boundary conditions in the relationship between TFL and well-being [22,144]. However, for example, a study by Gregersen et al. [129] could not confirm occupational self-efficacy as a relevant moderator. Thus, the study of the optimal conditions under which TFL leaders most effectively exert their influence warrants further research. Moreover, since the results of our study question the mediating effect of personal resources, it would be valuable to compare their mediating and moderating potential in one study to see how they can be installed by leaders to create the greatest benefit for employees.

Lastly, the concept of TFL is not without critique [19,20]. The ambiguity regarding the scale construction of this concept gives rise to the question of the rightful usage of this concept. Thus, it would certainly be valuable to confirm the findings of this study regarding more stringent leadership concepts to verify the results for leadership in general. Moreover, previous research has identified facets of TFL to be overtaxing to employees, leading to negative well-being outcomes [21,23]. Our study, however, could not support these negative effects. Thus, the question of the negative impacts of certain TFL facets remains equivocal. Unfortunately, we were not able to explore the impact of subdimensions of TFL in our models due to insufficient studies reporting on them. It would be valuable for future research to incorporate subdimensions of TFL to gain a more differentiated picture of TFL behaviors and resolve the inconsistencies in study findings [1]. 

## 6. Practical Implications

The involvement of leaders in promoting their employees’ health is increasingly considered essential for effective occupational health promotion [2,145]. We found in our study that work characteristics are an important means by which TFL leaders can indirectly influence their employees’ well-being. In particular, our study hints at the possibility that the provision of organizational resources could be an important leverage for them. Additionally, their position as role models and messengers of organizational resources makes them ideal “carriers” of organizational core values and proceedings. However, one has to keep in mind that the ordering of the variables could also be different. For example, TFL leaders could be more prevalent in organizations with high organizational resources (e.g., a positive health-specific organizational climate). Thus, again, the longitudinal confirmation of the results is needed. 

Leaders and organizations alike might not be aware of their power and responsibility in this regard and need to be trained to effectively apply this knowledge. In this regard, TFL leaders should, for example, learn to represent and live by organizational values, involve their employees and treat them fairly. This promotes an organizational culture of support, respect and authenticity, which enhances the well-being of employees [146]. Additionally, establishing a climate for innovation by the (structural) empowerment of employees, timely feedback and the transportation of organizational support should be especially well handled by TFL leaders due to their change-oriented leadership competencies [126].

Furthermore, TFL leaders should be aware of how finely nuanced their possibilities for influencing employee well-being are: for example, increasing job demands is highly associated with increases in negative well-being, while the enhancement of job resources both increases positive well-being (especially affective–motivational well-being) and decreases negative well-being. The improvement of work characteristics seems especially important to gain calm, relaxed and content employees since direct leadership efforts are in vain here.

Since leaders often do not have psychological work knowledge and are not aware of their potential influence as a designer of their employees’ work characteristics and work environment, organizations should offer this knowledge through training interventions and enable their TFL leaders to enhance and protect their employees’ well-being by modifying their work characteristics. Since the scope of leaders in this regard is always tied to organizational boundaries, this topic also requires the support of the organization at large.

## 7. Conclusions

The aim of our study was to shed light on the indirect mechanisms of the TFL–well-being relationship by examining the relative impact of various types of job resources and demands on TFL and well-being. The mediation patterns differed for each type of work characteristic and each indicator of well-being, indicating the complexity by which TFL leaders influence employee well-being. While all job resources and job demands were relevant mediators, organizational resources were identified as the most relevant mediators in the TFL–well-being relationship. These findings provide new insights on the importance of organizational resources in the scope of influence of TFL leaders and contribute to a new prioritization of different types of resources in TFL research. The results of our study will hopefully provide guidance on the indirect mechanisms between TFL and employee well-being for researchers and practitioners seeking to develop new research designs and effective health-promoting interventions. 

## Figures and Tables

**Table 1:** Indirect effects of the Job Resources Model, the Job Demands Model and the Comparison Model.

Mediator (M)	Dependent Variable (DV)	*β*_1_ (TFL -> M)	95%-CI	*β*_2_ (M -> DV)	95%-CI	*β_i_* (Indirect)	95%-CI
*the Job Resources Model*							
PERS	AMWB	0.30	[0.27, 0.33]	0.31	[0.28, 0.35]	0.09	[0.08, 0.11]
TASK	AMWB	0.42	[0.39, 0.45]	0.29	[0.24, 0.34]	0.12	[0.10, 0.15]
ORG	AMWB	0.56	[0.52, 0.59]	0.31	[0.25, 0.36]	0.17	[0.14, 0.21]
REL	AMWB	0.43	[0.39, 0.47]	0.25	[0.19, 0.31]	0.11	[0.08, 0.14]
total effect:						0.37	[0.34, 0.39]
PERS	PRWB			0.23	[0.16, 0.29]	0.07	[0.05, 0.09]
TASK	PRWB			0.22	[0.15, 0.30]	0.09	[0.06, 0.13]
ORG	PRWB			0.47	[0.35, 0.59]	0.26	[0.19, 0.33]
REL	PRWB			0.26	[0.16, 0.35]	0.11	[0.07, 0.16]
total effect:						0.32	[0.28, 0.36]
PERS	DEWB			−0.26	[−0.30, −0.22]	−0.08	[−0.09, −0.07]
TASK	DEWB			−0.31	[−0.35, −0.28]	−0.13	[−0.15, −0.12]
ORG	DEWB			−0.39	[−0.45, −0.34]	−0.22	[−0.26, −0.18]
REL	DEWB			−0.33	[−0.38, −0.28]	−0.14	[−0.17, −0.12]
total effect:						−0.28	[−0.31, −0.26]
PERS	IDWB			−0.25	[−0.29, −0.20]	−0.07	[−0.09, −0.06]
TASK	IDWB			−0.26	[−0.30, −0.21]	−0.11	[−0.13, −0.09]
ORG	IDWB			−0.40	[−0.49, −0.32]	−0.22	[−0.28, −0.17]
REL	IDWB			−0.34	[−0.41, −0.26]	−0.15	[−0.18, −0.11]
total effect:						−0.20	[−0.23, −0.17]
*the Job Demands Model*							
CD	AMWB	−0.23	[−28, −0.17]	−0.26	[−0.31, −0.21]	0.06	[0.04, 0.08]
HD	AMWB	−0.30	[−0.33, −0.26]	−0.28	[−0.33, −0.23]	0.08	[0.07, 0.10]
total effect:						0.39	[0.36, 0.41]
CD	PRWB			−0.33	[−0.40, −0.25]	0.07	[0.05, 0.10]
HD	PRWB			−0.37	[−0.44, −0.29]	0.11	[0.08, 0.14]
total effect:						0.36	[0.32, 0.39]
CD	DEWB			0.47	[0.42, 0.51]	−0.11	[−0.13, −0.08]
HD	DEWB			0.35	[0.31, 0.38]	−0.10	[−0.12, −0.09]
total effect:						−0.29	[−0.31, −0.27]
CD	IDWB			0.39	[0.34, 0.44]	−0.09	[−0.11, −0.07]
HD	IDWB			0.37	[0.33, 0.41]	−0.11	[−0.13, −0.09]
total effect:						−0.22	[−0.25, −0.18]
*the Comparison Model*							
PERS	AMWB	0.35	[0.32, 0.37]	0.36	[0.32, 0.40]	0.12	[0.11, 0.14]
JR	AMWB	0.51	[0.49, 0.53]	0.30	[0.26, 0.34]	0.15	[0.13, 0.18]
JD	AMWB	−0.31	[−0.34, −0.28]	−0.16	[−0.20, −0.11]	0.05	[0.04, 0.06]
total effect:						0.39	[0.36, 0.42]
PERS	PRWB			0.32	[0.25, 0.39]	0.11	[0.09, 0.14]
JR	PRWB			0.24	[0.18, 0.31]	0.12	[0.09, 0.16]
JD	PRWB			−0.26	[−0.32, −0.20]	0.08	[0.06, 0.10]
total effect:						0.35	[0.31, 0.39]
PERS	DEWB			−0.27	[−0.31, −0.23]	−0.09	[−0.11, −0.08]
JR	DEWB			−0.22	[−0.25, −0.18]	−0.11	[−0.13, −0.09]
JD	DEWB			0.39	[0.36, 0.43]	−0.12	[−0.14, −0.11]
total effect:						−0.26	[−0.29, −0.24]
PERS	IDWB			−0.26	[−0.31, −0.21]	−0.09	[−0.11, −0.07]
JR	IDWB			−0.17	[−0.21, −0.12]	−0.08	[−0.11, −0.06]
JD	IDWB			0.40	[0.36, 0.44]	−0.13	[−0.14, −0.11]
total effect:						−0.19	[−0.22, −0.15]

Note: independent variable = TFL, 95%-CI = likelihood-based confidence intervals; TFL = transformational leadership, CD = challenge demands, HD = hindrance demands, PERS = personal resources, TASK = task-related resources, ORG = organizational resources, REL = relational resources, JR = job resources, JD = job demands, AMWB = affective–motivational well-being, PRWB = pleased–relaxed well-being, DEWB = depressed–exhaustive well- being, IDWB = irritated–distressed well-being.

**Table 2:** Indirect effects of the four models with all types of work characteristics and one well-being indicator.

Mediator (M)	Dependent Variable (DV)	*β*_1_ (TFL -> M)	95%-CI	*β*_2_ (M -> DV)	95%-CI	*β_i_* (Indirect)	95%-CI
CD	AMWB	−0.35	[−0.39, −0.31]	−0.05	[−0.12, 0.02]	−0.02	[−0.01, 0.04]
HD	AMWB	−0.44	[−0.47, −0.40]	−0.15	[−0.21, −0.09]	0.06	[0.04, 0.09]
PERS	AMWB	0.34	[0.31, 0.37]	0.32	[0.28, 0.35]	0.11	[0.09, 0.12]
TASK	AMWB	0.49	[0.47, 0.52]	0.27	[0.21, 0.33]	0.13	[0.10, 0.16]
ORG	AMWB	0.59	[0.56, 0.63]	0.27	[0.19, 0.34]	0.16	[0.11, 0.21]
REL	AMWB	0.49	[0.46, 0.53]	0.20	[0.12, 0.28]	0.10	[0.06, 0.14]
total effect:						0.39	[0.36, 0.41]
CD	PRWB			−0.15	[−0.25, −0.05]	0.05	[0.02, 0.09]
HD	PRWB			−0.23	[−0.32, −0.13]	0.10	[0.06, 0.14]
PERS	PRWB			0.18	[0.11, 0.26]	0.06	[0.04, 0.09]
TASK	PRWB			0.17	[0.08, 0.26]	0.08	[0.04, 0.13]
ORG	PRWB			0.19	[0.19, 0.35]	0.11	[0.01, 0.21]
REL	PRWB			0.13	[−0.00, −0.25]	0.06	[−0.00, 0.12]
total effect:						0.34	[0.30, 0.38]
CD	DEWB			0.30	[0.24, 0.36]	−0.10	[−0.13, −0.08]
HD	DEWB			0.27	[0.23, 0.32]	−0.12	[−0.14, −0.10]
PERS	DEWB			−0.18	[−0.23, −0.14]	−0.06	[−0.08, −0.05]
TASK	DEWB			−0.23	[−0.28, −0.19]	−0.12	[−0.14, −0.09]
ORG	DEWB			−0.25	[−0.32, −0.18]	−0.15	[−0.19, −0.11]
REL	DEWB			−0.21	[−0.27, −0.15]	−0.10	[−0.14, −0.07]
total effect:						−0.28	[−0.30, −0.25]
CD	IDWB			0.22	[0.16, 0.29]	−0.08	[−0.10, −0.05]
HD	IDWB			0.33	[0.28, 0.38]	−0.14	[−0.16, −0.12]
PERS	IDWB			−0.18	[−0.23, −0.13]	−0.06	[−0.08, −0.04]
TASK	IDWB			−0.16	[−0.22, −0.10]	−0.12	[−0.11, −0.05]
ORG	IDWB			−0.16	[−0.27, −0.05]	−0.15	[−0.16, −0.03]
REL	IDWB			−0.18	[−0.27, −0.09]	−0.10	[−0.14, −0.04]
total effect:						−0.20	[−0.24, −0.17]

Note: independent variable = TFL, 95%-CI = likelihood-based confidence intervals; numbers in italics = nonsignificant, TFL = transformational leadership, CD = challenge demands, HD = hindrance demands, PERS = personal resources, TASK = task-related resources, ORG = organizational resources, REL = relational resources, JR = job resources, JD = job demands, AMWB = affective–motivational well-being, PRWB = pleased–relaxed well-being, DEWB = depressed–exhaustive well- being, IDWB = irritated–distressed well-being.

## Data Availability

All data and the R Markdown can be found at https://osf.io/c59q2/ (accessed on 26 January 2022).

## Supplementary Materials

The following supporting information can be downloaded at: https://www.mdpi.com/article/10.3390/ijerph19053133/s1, File S1: Analyses of publication bias for positive affect and emotional exhaustion; File S2: Significant moderator analyses and study quality; File S3: Reference list of all studies included in the analyses; Table S1: Search history; Table S2: Prisma checklist; Table S3: Coding manual; Table S4: Detailed information on the studies included in the analyses; Table S5: Full correlation matrices for SEM. Data, R Markdowns and forest plots can be viewed on https://osf.io/c59q2/ (accessed on 26 January 2022).

## Appendix A. Three-Level Meta-Analyses on TFL, Indicators of Well-Being and Mediators

**Table A1:** Three-Level Meta-Analyses of TFL with all Well-Being Indicators and their Subcomponents.

Variables	*N*	*k_s_*	*k_c_*	*r_c_*	*r*	*SE*	95%-CI	*τ*_(2)_^2^	*τ*_(3)_^2^	*I* ^2^_(3)_
**positive well-being**	154,858	148	172	0.41	0.37	0.01	[0.39, 0.44]	0.004	0.01	0.69
*affective-motivational*	45,629	106	121	0.43	0.39	0.01	[0.41, 0.46]	0.004	0.01	0.65
work engagement	39,173	89	98	0.44	0.39	0.01	[0.41, 0.47]	0.002	0.01	0.74
positive affect	8503	22	23	0.38	0.35	0.03	[0.33, 0.44]	0.01	0.003	0.18
*pleased-relaxed*	114,430	50	51	0.38	0.34	0.02	[0.35, 0.41]	<0.001	0.009	0.91
**negative well-being**	152,720	151	257	−0.27	−0.24	0.01	[−0.29, −0.25]	0.006	0.006	0.45
*depressed-exhaustive*	39,005	87	138	−0.32	−0.28	0.01	[−0.34, −0.29]	0.005	0.007	0.50
burnout	11,069	22	23	−0.30	−0.27	0.03	[−0.37, −0.24]	<0.001	0.02	0.89
emotional exhaustion	27,427	63	65	−0.32	−0.28	0.01	[−0.35, −0.29]	0.008	<0.001	0.00
depersonalization	8547	27	28	−0.33	−0.29	0.02	[−0.38, −0.28]	0.01	<0.001	0.00
RPA	4135	16	16	−0.28	−0.25	0.03	[−0.34, −0.22]	0.005	0.005	0.36
depression	2292	6	6	−0.32	−0.28	0.05	[−0.42, −0.23]	0.005	0.005	0.39
*irritated-distressed*	31,015	64	75	−0.22	−0.20	0.01	[−0.25, −0.20]	0.002	0.006	0.61
negative affect	4232	9	9	−0.19	−0.17	0.03	[−0.24, −0.14]	0.002	0.002	0.29
job stress	10,918	29	33	−0.26	−0.23	0.02	[−0.30, −0.22]	<0.001	0.009	0.76
irritation	16,860	29	33	−0.20	−0.18	0.02	[−0.23, −0.17]	0.005	<0.001	0.00

*Note: N* = sample size; *k_s_* = number of samples; *k_c_* = number of correlations; *r_c_* = corrected correlation coefficient; *r* = uncorrected correlation coefficient; *SE* = standard error; 95%-CI = Wald confidence intervals for *r_c_*; *τ*_(2)_^2^ = residual, variance; *τ*_(3)_^2^ = estimate of variance between studies; *I*^2^_(3)_ = proportion of total variation due to between-study heterogeneity; RPA = reduced personal accomplishment.

**Table A2:** Three-Level Meta-Analyses of the Mediators with TFL and Well-Being.

Variables	*N*	*k_s_*	*k_c_*	*r_c_*	*r*	*SE*	95%-CI	*τ*_(2)_^2^	*τ*_(3)_^2^	*I* ^2^_(3)_
***Job demands***										
*challenge demands (CD)*										
TFL~cd	12,538	19	20	−0.25	−0.22	0.03	[−0.31, −0.18]	0.002	0.02	0.83
cd~amwb	5128	7	18	−0.20	−0.17	0.05	[−0.30, −0.11]	0.02	0.002	0.09
cd~prwb	4382	6	9	−0.26	−0.22	0.06	[−0.37, −0.15]	0.02	0.004	0.19
cd~dewb	7475	13	25	0.49	0.41	0.04	[0.41, 0.58]	0.01	0.02	0.57
cd~idwb	7878	10	20	0.33	0.28	0.03	[0.27, 0.39]	0.01	<0.001	0.00
*hindrance demands (HD)*										
TFL~hd	17,789	25	49	−0.33	−0.29	0.03	[−0.31, −0.18]	0.02	0.005	0.19
hd~amwb	8723	9	32	−0.30	−0.26	0.05	[−0.40, −0.20]	0.01	0.01	0.40
hd~prwb	4565	7	12	−0.38	−0.32	0.03	[−0.43, −0.33]	0.002	0.002	0.39
hd~dewb	7393	14	65	0.42	0.35	0.02	[0.37, 0.46]	0.03	<0.001	0.00
hd~idwb	10,718	13	51	0.41	0.34	0.03	[0.35, 0.47]	0.02	0.007	0.26
***Job resources***										
*personal resources (PERS)*										
TFL~pers	28,434	61	80	0.31	0.27	0.02	[0.26, 0.35]	0.03	<0.001	0.00
pers~amwb	12,329	33	67	0.47	0.40	0.03	[0.41, 0.54]	0.02	0.02	0.47
pers~prwb	4588	7	17	0.35	0.30	0.02	[0.30, 0.39]	0.01	<0.001	0.00
pers~dewb	13,215	20	54	−0.33	−0.27	0.04	[−0.40, −0.26]	0.02	0.02	0.48
pers~idwb	10,862	20	36	−0.25	−0.21	0.02	[−0.30, −0.20]	0.02	<0.001	0.00
*task-related r. (TASK)*										
TFL~task	16,577	40	71	0.44	0.38	0.02	[0.40, 0.48]	0.02	0.005	0.22
task~amwb	9273	21	41	0.49	0.40	0.06	[0.38, 0.60]	0.02	0.003	0.13
task~prwb	4660	9	17	0.39	0.33	0.04	[0.32, 0.47]	0.001	0.01	0.80
task~dewb	7834	17	72	−0.39	−0.31	0.04	[−0.47, −0.32]	0.007	0.02	0.70
task~idwb	8031	11	38	−0.22	−0.17	0.04	[−0.31, −0.13]	0.004	0.02	0.79
*organizational r. (ORG)*										
TFL~org	22,263	37	46	0.57	0.51	0.03	[0.52, 0.63]	0.02	0.01	0.44
org~amwb	10,516	24	36	0.48	0.42	0.04	[0.41, 0.56]	0.005	0.03	0.78
org~prwb	9121	5	6	0.40	0.35	0.05	[0.30, 0.49]	<0.001	0.01	0.93
org~dewb	11,550	15	42	−0.38	−0.32	0.03	[−0.45, −0.32]	0.02	0.009	0.34
org~idwb	6338	6	13	−0.21	−0.18	0.05	[−0.31, −0.11]	0.005	0.01	0.61
*relational resources (REL)*										
TFL~rel	17,126	28	32	0.46	0.40	0.02	[0.42, 0.50]	0.02	<0.001	0.00
rel~amwb	8082	15	21	0.40	0.34	0.03	[0.34, 0.45]	0.01	<0.001	0.00
rel~prwb	4394	6	8	0.31	0.26	0.04	[0.24, 0.39]	<0.001	0.006	0.80
rel~dewb	12,528	16	36	−0.36	−0.30	0.03	[−0.42, −0.31]	0.008	0.006	0.39
rel~idwb	5422	8	14	−0.28	−0.24	0.03	[−0.34, −0.23]	0.004	0.002	0.31

*Note: N* = sample size; *k_s_* = number of samples; *k_c_* = number of correlations; *r_c_* = corrected correlation coefficient; *r* = uncorrected correlation coefficient; *SE* = standard error; 95%-CI = Wald confidence intervals for *r_c_*; *τ*_(2)_^2^ = residual variance; *τ*_(3)_^2^ = estimate of variance between studies; *I*^2^_(3)_ = proportion of total variation due to between-study heterogeneity; RPA = reduced personal accomplishment; amwb = affective-motivational well-being; prwb = pleased-relaxed well-being; dewb = depressed-exhaustive well-being; idwb = irritated-distressed well-being.

A reference list of all studies included in the analyses can be found in File S3.

## References

[1] Arnold K.A. (2017). Transformational leadership and employee psychological well-being: A review and directions for future research. J. Occup. Health Psychol..

[2] Vincent-Höper S., Teetzen F., Gregersen S., Nienhaus A. (2017). Leadership and employee well-being. Research Handbook on Work and Well-Being.

[3] Skakon J., Nielsen K., Borg V., Guzman J. (2010). Are leaders’ well-being, behaviours and style associated with the affective well-being of their employees? A systematic review of three decades of research. Work Stress.

[4] Wegge J., Shemla M., Haslam S.A. (2014). Leader Behavior as a Determinant of Health at Work: Specification and Evidence of Five Key Pathways. Ger. J. Hum. Resour. Manag..

[5] Hentrich S., Zimber A., Garbade S.F., Gregersen S., Nienhaus A., Petermann F. (2017). Relationships between transformational leadership and health: The mediating role of perceived job demands and occupational self-efficacy. Int. J. Stress Manag..

[6] Vincent-Höper S., Stein M. (2019). The Role of Leaders in Designing Employees’ Work Characteristics: Validation of the Health- and Development-Promoting Leadership Behavior Questionnaire. Front. Psychol..

[7] Tafvelin S., Armelius K., Westerberg K. (2011). Toward Understanding the Direct and Indirect Effects of Transformational Leadership on Well-Being: A Longitudinal Study. J. Leadersh. Organ. Stud..

[8] Breevaart K., Bakker A.B., Demerouti E., Sleebos D.M., Maduro V. (2014). Uncovering the Underlying Relationship between Transformational Leaders and Followers’ Task Performance. J. Pers. Psychol..

[9] Fernet C., Trépanier S.-G., Austin S., Gagné M., Forest J. (2015). Transformational leadership and optimal functioning at work: On the mediating role of employees’ perceived job characteristics and motivation. Work Stress.

[10] Inceoglu I., Thomas G., Chu C., Plans D., Gerbasi A. (2018). Leadership behavior and employee well-being: An integrated review and a future research agenda. Leadersh. Q..

[11] Demerouti E., Bakker A.B., Nachreiner F., Schaufeli W. (2001). The job demands-resources model of burnout. J. Appl. Psychol..

[12] Schaufeli W.B. (2015). Engaging leadership in the job demands-resources model. Career Dev. Int..

[13] Montano D., Reeske A., Franke F., Hüffmeier J. (2017). Leadership, followers’ mental health and job performance in organizations: A comprehensive meta-analysis from an occupational health perspective: Leadership and Followers’ Mental Health. J. Organ. Behav..

[14] Harms P.D., Credé M., Tynan M., Leon M., Jeung W. (2017). Leadership and stress: A meta-analytic review. Leadersh. Q..

[15] Bass B.M. (1985). Leadership and Performance beyond Expectations.

[16] Hoch J.E., Bommer W.H., Dulebohn J.H., Wu D. (2018). Do Ethical, Authentic, and Servant Leadership Explain Variance Above and Beyond Transformational Leadership? A Meta-Analysis. J. Manag..

[17] Avolio B.J., Bass B.M. (2004). Multifactor Leadership Questionnaire (MLQ).

[18] Rowold J., Borgmann L. (2013). Are leadership constructs really independent?. Leadersh. Organ. Dev. J..

[19] van Knippenberg D., Sitkin S.B. (2013). A Critical Assessment of Charismatic—Transformational Leadership Research: Back to the Drawing Board?. Acad. Manag. Ann..

[20] Yukl G. (1999). An evaluation of the conceptual weaknesses in transformational and charismatic leadership theories. Leadersh. Q..

[21] Diebig M., Bormann K.C., Rowold J. (2016). A double-edged sword: Relationship between full-range leadership behaviors and followers’ hair cortisol level. Leadersh. Q..

[22] Stein M., Schümann M., Vincent-Höper S. (2021). A Conservation of Resources View of the Relationship between Transformational Leadership and Emotional Exhaustion: The Role of Extra Effort and Psychological Detachment. Work Stress.

[23] Syrek C.J., Antoni C.H. (2014). Unfinished tasks foster rumination and impair sleeping—Particularly if leaders have high performance expectations. J. Occup. Health Psychol..

[24] Franke F., Felfe J. (2011). How does transformational leadership impact employees’ psychological strain?: Examining differentiated effects and the moderating role of affective organizational commitment. Leadership.

[25] Diener E., Seligman M.E.P. (2004). Toward an Economy of Well-Being. Psychol. Sci. Public Interest.

[26] Wright T.A., Emich K.J., Klotz D., Burke R.J., Page K.M. (2017). The many “faces” of well-being. Work and Well-Being.

[27] Salanova M., Del Líbano M., Llorens S., Schaufeli W.B. (2014). Engaged, Workaholic, Burned-Out or Just 9-to-5? Toward a Typology of Employee Well-being: Employee Well-being and Work Investment. Stress Health.

[28] Russell J.A., Carroll J.M. (1999). On the Bipolarity of Positive and Negative Affect. Psychol. Bull..

[29] Warr P. (1990). The measurement of well-being and other aspects of mental health. J. Occup. Psychol..

[30] Deci E.L., Ryan R.M. (2008). Hedonia, eudaimonia, and well-being: An introduction. J. Happiness Stud..

[31] Shamir B., House R.J., Arthur M.B. (1993). The Motivational Effects of Charismatic Leadership: A Self-Concept Based Theory. Organ. Sci..

[32] Sivanathan N., Arnold K.A., Turner N., Barling J. (2004). Leading well: Transformational leadership and well-being. Positive Psychology in Practice.

[33] Berkman L.F. (1995). The Role of Social Relations in Health Promotion. Psychosom. Med..

[34] Munir F., Nielsen K., Carneiro G.I. (2010). Transformational leadership and depressive symptoms: A prospective study. J. Affect. Disord..

[35] Bakker A., Demerouti E., Schaufeli W. (2003). Dual processes at work in a call centre: An application of the job demands—Resources model. Eur. J. Work Organ. Psychol..

[36] Breevaart K., Bakker A.B., Hetland J., Hetland H. (2014). The influence of constructive and destructive leadership behaviors on follower burnout. Burnout at Work: A Psychological Perspective.

[37] Kelloway E.K., Sivanathan N., Francis L., Barling J. (2005). Poor Leadership. Handbook of Workplace Stress.

[38] Liu J., Siu O.-L., Shi K. (2009). Transformational Leadership and Employee Well-Being: The Mediating Role of Trust in the Leader and Self-Efficacy: Transformational Leadership. Appl. Psychol. Int. Rev..

[39] Nielsen K., Nielsen M.B., Ogbonnaya C., Känsälä M., Saari E., Isaksson K. (2017). Workplace resources to improve both employee well-being and performance: A systematic review and meta-analysis. Work Stress.

[40] Hawkes A.J., Biggs A., Hegerty E. (2017). Work Engagement: Investigating the Role of Transformational Leadership, Job Resources, and Recovery. J. Psychol..

[41] Gilbert M.-H., Dagenais-Desmarais V., St-Hilaire F. (2017). Transformational leadership and autonomy support management behaviors: The role of specificity in predicting employees’ psychological health. Leadersh. Organ. Dev. J..

[42] Javeed T., Farooqi Y.A. (2013). Impact of Transformational Leadership Style on Employees’ Satisfaction and Well-Being with Working Conditions as Mediator. Int. J. Multidiscip. Sci. Eng..

[43] Besieux T., Baillien E., Verbeke A.L., Euwema M.C. (2015). What goes around comes around: The mediation of corporate social responsibility in the relationship between transformational leadership and employee engagement. Econ. Ind. Democr..

[44] Dimoff J.K., Kelloway E.K., Kelloway E.K., Nielsen K., Dimoff J.K. (2017). Leaders as resources: How managers and supervisors can socially support employees towards better mental health and wellbeing. Leading to Occupational Health and Safety.

[45] Kark R., Shamir B. (2002). The dual effect of transformational leadership: Priming relational and collective selves and further effects on followers. Transformational and Charismatic Leadership: The Road Ahead.

[46] Cho J., Dansereau F. (2010). Are transformational leaders fair? A multi-level study of transformational leadership, justice perceptions, and organizational citizenship behaviors. Leadersh. Q..

[47] Gillet N., Fouquereau E., Bonnaud-Antignac A., Mokounkolo R., Colombat P. (2013). The mediating role of organizational justice in the relationship between transformational leadership and nurses’ quality of work life: A cross-sectional questionnaire survey. Int. J. Nurs. Stud..

[48] Jena L.K., Pradhan S., Panigrahy N.P. (2018). Pursuit of organisational trust: Role of employee engagement, psychological well-being and transformational leadership. Asia Pac. Manag. Rev..

[49] Walsh M., Dupre K., Arnold K.A. (2014). Processes through Which Transformational Leaders Affect Employee Psychological Health. Ger. J. Hum. Resour. Manag. Z. Pers..

[50] Schaufeli W.B., Bakker A.B. (2004). Job demands, job resources, and their relationship with burnout and engagement: A multi-sample study. J. Organ. Behav..

[51] Bakker A.B., Hakanen J.J., Demerouti E., Xanthopoulou D. (2007). Job resources boost work engagement, particularly when job demands are high. J. Educ. Psychol..

[52] Bakker A.B., Xanthopoulou D. (2013). Creativity and charisma among female leaders: The role of resources and work engagement. Int. J. Hum. Resour. Manag..

[53] Vincent-Höper S., Gregersen S., Nienhaus A. (2017). Do Work Characteristics Mediate the Negative Effect of Transformational Leadership on Impaired Well-Being?. Z. Arb. Organ. AO.

[54] Hobfoll S.E. (1989). Conservation of Resources: A new attempt at conceptualizing stress. Am. Psychol..

[55] Hobfoll S.E., Freedy J., Lane C., Geller P. (1990). Conservation of social resources: Social support resource theory. J. Soc. Pers. Relatsh..

[56] Ghadi M., Fernando M., Caputi P. (2013). Transformational leadership and work engagement: The mediating effect of meaning in work. Leadersh. Organ. Dev. J..

[57] Nielsen K., Randall R., Yarker J., Brenner S.-O. (2008). The effects of transformational leadership on followers’ perceived work characteristics and psychological well-being: A longitudinal study. Work Stress.

[58] Holstad T.J., Rigotti T., Otto K. (2013). Prozedurale Fairness als Mediator zwischen transformationaler Führung und psychischer Beanspruchung am Arbeitsplatz: Eine Mehrebenenstudie. Z. Arb. Organ. AO.

[59] Lewis H.S., Cunningham C.J.L. (2016). Linking Nurse Leadership and Work Characteristics to Nurse Burnout and Engagement. Nurs. Res..

[60] Schmidt B., Loerbroks A., Herr R., Litaker D., Wilson M., Kastner M., Fischer J. (2014). Psychosocial resources and the relationship between transformational leadership and employees’ psychological strain. Work.

[61] Dubinsky A.J., Yammarino F.J., Jolson M.A., Spangler W.D. (2013). Transformational Leadership: An Initial Investigation in Sales Management. J. Pers. Sell. Sales Manag..

[62] Green A.E., Albanese B.J., Shapiro N.M., Aarons G.A. (2014). The roles of individual and organizational factors in burnout among community-based mental health service providers. Psychol. Serv..

[63] Cavanaugh M.A., Boswell W.R., Roehling M.V., Boudreau J.W. (2000). An Empirical Examination of Self-Reported Work Stress among U.S. Managers. J. Appl. Psychol..

[64] Bass B.M. (1998). Transformational Leadership: Industry, Military, and Educational Impact.

[65] Rafferty A.E., Griffin M.A. (2004). Dimensions of transformational leadership: Conceptual and empirical extensions. Leadersh. Q..

[66] Dionne S.D., Yammarino F.J., Atwater L.E., Spangler W.D. (2004). Transformational leadership and team performance. J. Organ. Chang. Manag..

[67] Syrek C.J., Apostel E., Antoni C.H. (2013). Stress in highly demanding IT jobs: Transformational leadership moderates the impact of time pressure on exhaustion and work–Life balance. J. Occup. Health Psychol..

[68] Danna K., Griffin R.W. (1999). Health and Well-Being in the Workplace: A Review and Synthesis of the Literature. J. Manag..

[69] Bakker A.B., Demerouti E., Verbeke W. (2004). Using the job demands-resources model to predict burnout and performance. Hum. Resour. Manag..

[70] Bakker A.B., Demerouti E., Euwema M.C. (2005). Job Resources Buffer the Impact of Job Demands on Burnout. J. Occup. Health Psychol..

[71] Llorens S., Bakker A.B., Schaufeli W., Salanova M. (2006). Testing the robustness of the job demands-resources model. Int. J. Stress Manag..

[72] Crawford E.R., LePine J.A., Rich B.L. (2010). Linking job demands and resources to employee engagement and burnout: A theoretical extension and meta-analytic test. J. Appl. Psychol..

[73] Breevaart K., Bakker A.B. (2018). Daily job demands and employee work engagement: The role of daily transformational leadership behavior. J. Occup. Health Psychol..

[74] Tadić M., Bakker A.B., Oerlemans W.G.M. (2015). Challenge versus hindrance job demands and well-being: A diary study on the moderating role of job resources. J. Occup. Organ. Psychol..

[75] Gorgievski M.J., Hobfoll S.E. (2008). Work can burn us out or fire us up: Conservation of resources in burnout and engagement. Handbook of Stress and Burnout in Health Care.

[76] Albrecht S.L. (2015). Challenge Demands, Hindrance Demands, and Psychological Need Satisfaction: Their Influence on Employee Engagement and Emotional Exhaustion. J. Pers. Psychol..

[77] LePine J.A., Podsakoff N.P., LePine M.A. (2005). A Meta-Analytic Test of the Challenge Stressor–Hindrance Stressor Framework: An Explanation for Inconsistent Relationships Among Stressors and Performance. Acad. Manag. J..

[78] Oates M.J. (2012). How Do Transformational Leaders Reduce Managerial Stress? A Comparison of Mediated and Moderated Models.

[79] Berger R., Czakert J.P., Leuteritz J.-P., Leiva D. (2019). How and When Do Leaders Influence Employees’ Well-Being? Moderated Mediation Models for Job Demands and Resources. Front. Psychol..

[80] Diebig M., Bormann K.C., Rowold J. (2017). Day-level transformational leadership and followers’ daily level of stress: A moderated mediation model of team cooperation, role conflict, and type of communication. Eur. J. Work Organ. Psychol..

[81] Avolio B.J., Bass B.M. (1994). Multifactor Leadership Questionnaire (MLQ). Manual and Sampler Set.

[82] Carless S.A., Wearing A.J., Mann L. (2000). A Short Measure of Transformational Leadership. J. Bus. Psychol..

[83] Podsakoff P.M., MacKenzie S.B., Bommer W.H. (1996). Transformational leader behaviors and substitutes for leadership as determinants of employee satisfaction, commitment, trust, and organizational citizenship behaviors. J. Manag..

[84] Kaluza A.J., Boer D., Buengeler C., van Dick R. (2020). Leadership behaviour and leader self-reported well-being: A review, integration and meta-analytic examination. Work Stress.

[85] Warr P., Wang M., Sinclair R.R., Tetrick L.E. (2013). How to Think About and Measure Psychological Well-being. Research Methods in Occupational Health Psychology.

[86] Judge T.A., Piccolo R.F. (2004). Transformational and Transactional Leadership: A Meta-Analytic Test of Their Relative Validity. J. Appl. Psychol..

[87] Warr P., Cook J.D., Wall T.D. (1979). Scales for the measurement of some work attitudes and aspects of psychological well-being. J. Occup. Organ. Psychol..

[88] Watson D., Anna L., Tellegen A. (1988). Development and Validation of Brief Measures of Positive and Negative Affect: The PANAS Scales. J. Pers. Soc. Psychol..

[89] Schaufeli W.B., Salanova M., González-Romá V., Bakker A.B. (2002). The Measurement of Engagement and Burnout: A Two Sample Confirmatory Factor Analytic Approach. J. Happiness Stud..

[90] van Katwyk P.T., Fox S., Spector P., Kelloway E.K. (2000). Using the Job-Related Affective Well-Being Scale (JAWS) to investigate affective responses to work stressors. J. Occup. Health Psychol..

[91] Bech P. (2004). Measuring the dimension of psychological general well-being by the WHO-5. Qual. Life Newsl..

[92] Goldberg D., Williams P.A. (1988). A User’s Guide to the General Health Questionnaire.

[93] Graham L., van Witteloostuijn A., Tjalling C. (2010). Leader-member exchange, communication frequency and burnout. Proceedings of the Discussion Paper Series.

[94] Maslach C., Jackson S.E. (1981). The measurement of experienced burnout. J. Organ. Behav..

[95] Gillespie D.F., Numerof R.E. (1984). The Development of a Unidimensional Measure of Burnout: The GNBI (Working Paper).

[96] Kristensen T.S., Hannerz H., Høgh A., Borg V. (2005). The Copenhagen Psychosocial Questionnaire—A tool for the assessment and improvement of the psychosocial work environment. Scand. J. Work Environ. Health.

[97] Mohr G., Müller A., Rigotti T., Aycan Z., Tschan F. (2006). The Assessment of Psychological Strain in Work Contexts. Eur. J. Psychol. Assess..

[98] Lovibond S.H., Lovibond P.F. (1993). Manual for the Depression Anxiety Stress Scales (DASS).

[99] Cohen S., Kamarck T., Mermelstein R. (1983). A Global Measure of Perceived Stress. J. Health Soc. Behav..

[100] Rigotti T., Schyns B., Mohr G. (2008). A Short Version of the Occupational Self-Efficacy Scale: Structural and Construct Validity Across Five Countries. J. Career Assess..

[101] de Jonge J., Reuvers M.M.E.N., Houtman I.L.D., Bongers P.M., Kompier M.A.J. (2000). Linear and nonlinear relations between psychosocial job characteristics, subjective outcomes, and sickness absence: Baseline results from SMASH. J. Occup. Health Psychol..

[102] Leiter M.P., Maslach C. (2003). Areas of worklife: A structured approach to organizational predictors of job burnout. Research in Occupational Stress and Well-Being.

[103] Semmer N.K., Zapf D., Dunckel H. (1999). Instrument zur Stressbezogenen Tätigkeitsanalyse (Instrument for stress-related job analysis (ISTA). Handbuch Psychologischer Arbeitsanalyseverfahren.

[104] Rizzo J.R., House R.J., Lirtzman S.I. (1970). Role conflict and ambiguity in complex organizations. Adm. Sci. Q..

[105] R Core Team R: A Language and Environment for Statistical Computing. https://www.r-project.org/.

[106] Viechtbauer W. (2010). Conducting Meta-Analyses in *R* with the metafor Package. J. Stat. Softw..

[107] Cheung M.W.-L. (2015). metaSEM: An R package for meta-analysis using structural equation modeling. Front. Psychol..

[108] Rosseel Y. (2012). lavaan: An R package for structural equation modeling and more Version 0.5-12 (BETA). J. Stat. Softw..

[109] Conigrave J. (2019). Msemtools: Routines, Tables and Figures for metaSEM Analyses. https://github.com/JConigrave/msemtools.

[110] Wiernik B.M., Dahlke J.A. (2020). Obtaining Unbiased Results in Meta-Analysis: The Importance of Correcting for Statistical Artefacts. Adv. Methods Pract. Psychol. Sci..

[111] Cheung M.W.-L. (2014). Modeling dependent effect sizes with three-level meta-analyses: A structural equation modeling approach. Psychol. Methods.

[112] Cheung S.F., Chan D.K.-S. (2004). Dependent Effect Sizes in Meta-Analysis: Incorporating the Degree of Interdependence. J. Appl. Psychol..

[113] van Den Noortgate W., Onghena P. (2003). Multilevel Meta-Analysis: A Comparison with Traditional Meta-Analytical Procedures. Educ. Psychol. Meas..

[114] Marín-Martínez F., Sánchez-Meca J. (2010). Weighting by Inverse Variance or by Sample Size in Random-Effects Meta-Analysis. Educ. Psychol. Meas..

[115] Cheung M.W.-L., Chan W. (2005). Meta-analytic structural equation modeling: A two-stage approach. Psychol. Methods.

[116] Wilson S.J., Polanin J.R., Lipsey M.W. (2016). Fitting meta-analytic structural equation models with complex datasets. Res. Synth. Methods.

[117] Cleophas T.J., Zwinderman A.H. (2017). Modern Meta-Analysis: Review and Update of Methodologies.

[118] Sterne J.A.C., Becker B.J., Egger M. (2005). The Funnel Plot. Publication Bias in Meta-Analysis: Prevention, Assessment and Adjustments.

[119] Egger M., Smith G.D., Schneider M., Minder C. (1997). Bias in meta-analysis detected by a simple, graphical test. BMJ.

[120] Duval S., Tweedie R. (2000). Trim and Fill: A Simple Funnel-Plot-Based Method of Testing and Adjusting for Publication Bias in Meta-Analysis. Biometrics.

[121] Tims M., Bakker A.B., Xanthopoulou D. (2011). Do transformational leaders enhance their followers’ daily work engagement?. Leadersh. Q..

[122] Breevaart K., Bakker A., Hetland J., Demerouti E., Olsen O.K., Espevik R. (2014). Daily transactional and transformational leadership and daily employee engagement. J. Occup. Organ. Psychol..

[123] Widmer P.S., Semmer N.K., Kälin W., Jacobshagen N., Meier L.L. (2012). The ambivalence of challenge stressors: Time pressure associated with both negative and positive well-being. J. Vocat. Behav..

[124] Li P., Taris T.W., Peeters M.C.W. (2020). Challenge and hindrance appraisals of job demands: One man’s meat, another man’s poison?. Anxiety Stress Coping.

[125] Faupel S., Süß S. (2019). The Effect of Transformational Leadership on Employees During Organizational Change—An Empirical Analysis. J. Chang. Manag..

[126] Monje Amor A., Abeal Vázquez J.P., Faíña J.A. (2020). Transformational leadership and work engagement: Exploring the mediating role of structural empowerment. Eur. Manag. J..

[127] Gilbert S.L., Kelloway E.K. (2018). Leadership, Recognition and Well-Being: A Moderated Mediational Model. Can. J. Adm. Sci. Rev. Can. Sci. Adm..

[128] Strom D.L., Sears K.L., Kelly K.M. (2014). Work Engagement: The Roles of Organizational Justice and Leadership Style in Predicting Engagement Among Employees. J. Leadersh. Organ. Stud..

[129] Gregersen S., Vincent-Höper S., Nienhaus A. (2014). The Relation Between Leadership and Perceived Well-Being: What Role Does Occupational Self-Efficacy Play?. J. Leadersh. Stud..

[130] Arnold K.A., Turner N., Barling J., Kelloway E.K., McKee M.C. (2007). Transformational leadership and psychological well-being: The mediating role of meaningful work. J. Occup. Health Psychol..

[131] Luchman J.N., González-Morales M.G. (2013). Demands, control, and support: A meta-analytic review of work characteristics interrelationships. J. Occup. Health Psychol..

[132] Gregersen S., Vincent-Höper S., Nienhaus A. (2016). Job-related resources, leader–member exchange and well-being—A longitudinal study. Work Stress.

[133] Perko K., Kinnunen U., Tolvanen A., Feldt T. (2016). Investigating occupational well-being and leadership from a person-centred longitudinal approach: Congruence of well-being and perceived leadership. Eur. J. Work Organ. Psychol..

[134] van Dierendonck D., Haynes C., Borrill C., Stride C. (2004). Leadership Behavior and Subordinate Well-Being. J. Occup. Health Psychol..

[135] Podsakoff P.M., MacKenzie S.B., Podsakoff N.P. (2012). Sources of Method Bias in Social Science Research and Recommendations on How to Control It. Annu. Rev. Psychol..

[136] Chan D. (2009). So why ask me? Are self-report data really that bad?. Statistical and Methodological Myths and Urban Legends.

[137] Paulhus D.L., Vazire S. (2009). The self-report method. Handbook of Research Methods in Personality Psychology.

[138] Sandvik E., Diener E., Seidlitz L. (1993). Subjective Well-Being: The Convergence and Stability of Self-Report and Non-Self-Report Measures. J. Pers..

[139] Crampton S.M., Wagner J.A. (1994). Percept-percept inflation in microorganizational research: An investigation of prevalence and effect. J. Appl. Psychol..

[140] Diener E., Lucas R.E., Scollon C.N. (2006). Beyond the hedonic treadmill: Revising the adaptation theory of well-being. Am. Psychol..

[141] Hildenbrand K., Sacramento C.A., Binnewies C. (2018). Transformational leadership and burnout: The role of thriving and followers’ openness to experience. J. Occup. Health Psychol..

[142] Perry S.J., Witt L.A., Penney L.M., Atwater L. (2010). The downside of goal-focused leadership: The role of personality in subordinate exhaustion. J. Appl. Psychol..

[143] De Hoogh A.H.B., Den Hartog D.N. (2009). Neuroticism and locus of control as moderators of the relationships of charismatic and autocratic leadership with burnout. J. Appl. Psychol..

[144] Zhu W., Avolio B.J., Walumbwa F.O. (2009). Moderating Role of Follower Characteristics With Transformational Leadership and Follower Work Engagement. Group Organ. Manag..

[145] Zimolong B., Elke G. (2006). Occupational health and safety management. Handbook of Human Factors and Ergonomics.

[146] Arfat Y., Rehman M., Mahmood K., Saleem R. (2017). The role of leadership in work engagement: The moderating role of a bureaucratic and supportive culture. Pak. Bus. Rev..

