# Supplementary material for: The Mediating Effects of Work Characteristics on the Relationship between Transformational Leadership and Employee Well-Being: A Meta-Analytic Investigation
Source: Int J Environ Res Public Health. 2022 Mar 7;19(5):3133. doi: 10.3390/ijerph19053133 (PMC8910295; doi:10.3390/ijerph19053133)
Supplement: Supplementary file 1 [file ijerph-19-03133-s001.zip › File S1.pdf]

File S1. Analyses of Publication Bias for Positive Affect and Emotional Exhaustion.

Significant tests for publication bias of the three-level meta-analyses of the outcomes *positive affect* and *emotional exhaustion*.

a) Positive affect

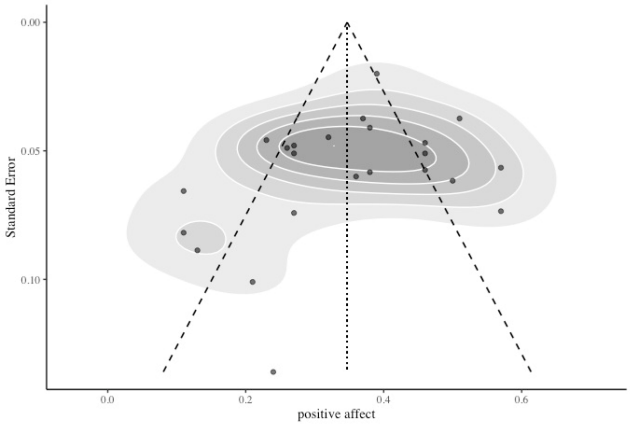

Figure S1a. Test for publication bias on the three-level meta-analysis of positive affect.

Egger's Test: z = -1.8205, p = 0.07

Trim and fill =
Estimated number of missing studies on the right side = 2, SE = 2.45, p = 0.13

Model results: estimate = 0.37, SE = 0.03, p = <0.001, CI [0.31; 0.43]

b) Emotional exhaustion

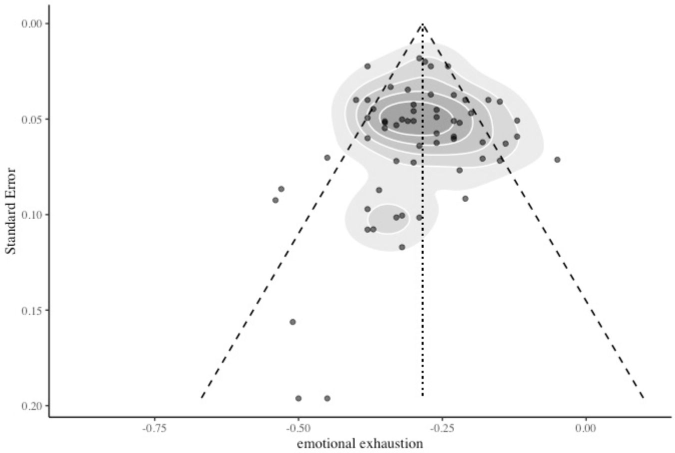

Figure S1b. Test for publication bias on the three-level meta-analysis of emotional exhaustion.

Egger's Test: z = -1.6615, p = 0.10

Trim and fill =
Estimated number of missing studies on the right side = 0, SE = 1.41, p = 0.5

Model results: estimate = -0.28, SE = 0.01, p = <0.001, CI [-0.31; -0.26]
