# Supplementary material for: The Mediating Effects of Work Characteristics on the Relationship between Transformational Leadership and Employee Well-Being: A Meta-Analytic Investigation
Source: Int J Environ Res Public Health. 2022 Mar 7;19(5):3133. doi: 10.3390/ijerph19053133 (PMC8910295; doi:10.3390/ijerph19053133)
Supplement: Supplementary file 1 [file ijerph-19-03133-s001.zip › File S2.pdf]

File S2. Significant Moderator Analyses and Study Quality.

When between-study heterogeneity in the relationship of TFL and employee well-being exceeded 50% (see Table 1 in the manuscript), we conducted moderator analyses with various categorial study level moderators (see Appendix Table below). The meta-analysis of TFL and positive well-being showed an $I^2_{(3)}$ = 0.69. A moderator analysis indicated the moderators *continent* (p = 0.05, R^2 = 0.08) and *industry of the sample* (p = 0.008, R^2 = 0.15). *Collectivistic countries* (k_c = 19, r = 0.49, *95%-CI* = [0.43, 0.55]), next to the *service sector* (k_c = 30, r = 0.48, *95%-CI* = [0.43, 0.53]) and *mixed samples* (k_c = 65, r = 0.41, *95%-CI* = [0.38, 0.44]), displayed stronger relationships of TFL and positive well-being than *European countries* (k_c = 78, r = 0.39, *95%-CI* = [0.36, 0.42]) and the *health care sector* (k_c = 26, r = 0.35, *95%-CI* = [0.30, 0.40]). Further analyses of the sub-components of positive well-being revealed that the TFL-work engagement relationship reflected the moderator *continent* (p = 0.04, R^2 = 0.13; *collectivistic countries*: k_c = 15, r = 0.53, *95%-CI* = [0.46, 0.60]; *European countries*: k_c = 40, r = 0.41, *95%-CI* = [0.36, 0.45]) and that the relationship between TFL and PRWB was moderated by *industry of the sample* (p = <.001, R^2 = 0.64). However, here, the *mixed samples* alone displayed the strongest relationships between TFL and PRWB (k_c = 24, r = 0.44, *95%-CI* = [0.41, 0.47]), while the *health care sector* (k_c = 8, r = 0.28, *95%-CI* = [0.23, 0.33]), *industry* (k_c = 15, r = 0.33, *95%-CI* = [0.28, 0.38]), and the *service sector* (k_c = 2, r = 0.25, *95%-CI* = [0.15, 0.35]) indicated significantly lower relationships. Note, however, that the n's of the subgroups were, in parts, very small.

Regarding the negative indicators of well-being, we found no significant moderator for the TFL-burnout relationship. Obviously, a moderator that was not examined must have been responsible for the heterogeneity ($I^2_{(3)}$ = 0.89) here. The relationship between TFL and job stress indicated the moderator *measurement of TFL* (p = 0.05, R^2 = 0.03). When TFL was measured by *other* instruments, the relationship between TFL and job stress was significantly stronger (k_c = 7, r = -0.39, *95%-CI* = [-0.46, -0.31]) than when it was measured by the *MLQ* or the *GTL* (*MLQ*: k_c = 18, r = -0.22 *95%-CI* = [-0.26, -0.17], *GTL*: k_c = 33, r = -0.26 *95%-CI* = [-0.30, -0.22]).

In sum, we found different moderators being relevant for different indicators and sub-components of well-being. The moderator *continent of the sample* seems to be a relevant moderator in the TFL-work engagement relationship, while the moderator *industry of the sample* seems to be of relevance to the TFL-PRWB relationship. The TFL-job stress relationship was moderated by the moderator *measurement of TFL*. However, this last moderator did not show on another aggregation level of well-being (e.g., negative well-being, IDWB). The moderator *study quality* did not yield any significant results. There was a tendency for dissertations to report the strongest results, especially compared to unpublished literature, however, the confidence intervals still overlapped, so that we cannot confidently interpret these findings as real differences.

Due to results being contingent on the level of analysis and since the relevance of the moderators, in part, was also shown to be very low (e.g., R^2 = 0.03 for the moderator *measurement of TFL*), we were hesitant to interpret the moderator results too confidently. Further research on the individual level (instead of the study level) could help to bring more light into relevant moderators of the TFL-well-being relationship.

Unfortunately, it was not possible to conduct subgroup analyses of the SEM's according to these found moderators due to insufficient data in each cell of the SEM's.

Complete moderator analyses can be found in the *R* Markdown on https://osf.io/c59q2/.

Appendix Table. Significant moderators in the TFL-well-being relationship.

**moderator category**	$I^2_{(3)}$	p	R^2	k_c	r	*95% CI*
TFL ~ positive well-being	0.69					
moderator = continent		0.05	0.08			
collectivistic countries				19	0.49	[0.43, 0.55]
others				32	0.42	[0.37, 0.47]
USA/Australia				43	0.41	[0.37, 0.45]

European countries				78	0.39	[0.36, 0.42]
moderator = industry of the sample		0.008	0.15			
service sector				30	0.48	[0.43, 0.53]
mixed samples				65	0.41	[0.38, 0.44]
educational sector				9	0.40	[0.31, 0.48]
public service				6	0.40	[0.30, 0.51]
industry samples				27	0.37	[0.32, 0.42]
other industry				9	0.37	[0.29, 0.46]
health care sector				26	0.35	[0.30, 0.40]
TFL ~ work engagement	0.74					
moderator = continent		0.04	0.13			
collectivistic countries				15	0.53	[0.46, 0.60]
European countries				40	0.41	[0.36, 0.45]
other countries				19	0.44	[0.38, 0.50]
USA/Australia				24	0.44	[0.38, 0.50]
TFL ~ PRWB	0.91					
moderator = industry of the sample		<0.001	0.69			
mixed samples				24	0.44	[0.41, 0.47]
educational sector				1	0.37	[0.26, 0.48]
industry samples				15	0.33	[0.28, 0.38]
health care sector				8	0.28	[0.23, 0.33]
other industry				1	0.27	[0.13, 0.41]
service sector				2	0.25	[0.15, 0.35]
TFL ~ job stress	0.76					
moderator = measurement of TFL		0.05	0.03			
other measurements				7	-0.39	[-0.46, -0.31]
raffgriff				5	-0.29	[-0.38, -0.20]
TLI				2	-0.26	[-0.38, -0.14]
MLQ				18	-0.22	[-0.26, -0.17]

Note: $I^2_{(3)}$ = between-study heterogeneity, k_c = number of correlations

Study Quality Assessment

We assessed the study quality of the primary studies by the impact factor of the journal the study was published in and differentiated between the categories “dissertations”, “unpublished literature/conference papers”, “no impact”, “low impact” and “high impact”. Moderator analyses indicated slightly higher associations between TFL and the well-being indicators (especially positive well-being) and a slightly lower correlation of TFL and well-being for unpublished literature/conference papers. However, the differences were very small (confidence intervals overlapped), so that we cannot confidently interpret these results. Due to these neglectable differences we do not see an influence of study quality of the primary studies on the results of our study. Complete analyses can be found on https://osf.io/c59q2/.
