# Supplementary material for: The Mediating Effects of Work Characteristics on the Relationship between Transformational Leadership and Employee Well-Being: A Meta-Analytic Investigation
Source: Int J Environ Res Public Health. 2022 Mar 7;19(5):3133. doi: 10.3390/ijerph19053133 (PMC8910295; doi:10.3390/ijerph19053133)
Supplement: Supplementary file 1 [file ijerph-19-03133-s001.zip › File S3.pdf]

**File S3. Reference List of References Included into the Analyses.**

1. Abate GB, Sewasew DT, Woldemeskel BM. THE MODERATING EFFECT OF SELF-EFFICACY ON THE RELATIONSHIPS BETWEEN TRANSFORMATIONAL LEADERSHIP AND SUBORDINATES HEALTH. Innovare Journal of social Sciences. 2014;2: 5.

2. Ali ZS. The Moderating Effect of Perceived Leadership Style On the Stressor-Strain Relationship in the Nonprofit Sector. Hofstra University. 2015.

3. Altansukh N. MEDIATION EFFECT OF WORK ENGAGEMENT ON RELATIONSHIP BETWEEN TRANSFORMATIONAL LEADERSHIP AND TASK PERFORMANCE AND MODERATION EFFECT OF ADVERSITY QUOTIENT AMONG WORK ENGAGEMENT AND TASK PERFORMANCE. Masters Thesis, Nanhua University. 2015.

4. Arfat Y, Rehman M, Mahmood K, Saleem R. THE ROLE OF LEADERSHIP IN WORK ENGAGEMENT: THE MODERATING ROLE OF A BUREAUCRATIC AND SUPPORTIVE CULTURE. Pakistan Business Review. 2017; 18.

5. Arifin F, Troena EA, Rahayu M. Organizational Culture, Transformational Leadership, Work Engagement and Teacher's Performance : Test of a Model. International Journal of Education and Research. 2014;2: 14.

6. Ariyani N, Hidayati S. Influence of Transformational Leadership and Work Engagement On Innovative Behavior. Etikonomi. 2018;17: 275–284. doi:10.15408/etk.v17i2.7427

7. Arnold KA, Turner N, Barling J, Kelloway EK, McKee MC. Transformational leadership and psychological well-being: The mediating role of meaningful work. Journal of Occupational Health Psychology. 2007;12: 193–203. doi:10.1037/1076-8998.12.3.193

8. Arnold KA, Walsh M. Customer incivility and employee well-being: testing the moderating effects of meaning, perspective taking and transformational leadership. Work & Stress. 2015;29: 362–378. doi:10.1080/02678373.2015.1075234

9. Aryee S, Walumbwa FO, Zhou Q, Hartnell CA. Transformational Leadership, Innovative Behavior, and Task Performance: Test of Mediation and Moderation Processes. Human Performance. 2012;25: 1–25. doi:10.1080/08959285.2011.631648

10. Bae SH, Song JH, Park S, Kim HK. Influential Factors for Teachers' Creativity: Mutual Impacts of Leadership, Work Engagement, and Knowledge Creation Practices. Perf Improvement Qrtly. 2013;26: 33–58. doi:10.1002/piq.21153

11. Bass BI, Cigularov KP, Chen PY, Henry KL, Tomazic RG, Li Y. The effects of student violence against school employees on employee burnout and work engagement: The roles of perceived school unsafety and transformational leadership. International Journal of Stress Management. 2016;23: 318–336. doi:10.1037/str0000011

12. Berger R, Czakert JP, Leuteritz J-P, Leiva D. How and When Do Leaders Influence Employees' Well-Being? Moderated Mediation Models for Job Demands and Resources. Front Psychol. 2019;10: 2788. doi:10.3389/fpsyg.2019.02788

13. Besieux T, Baillien E, Verbeke AL, Euwema MC. What goes around comes around: The mediation of corporate social responsibility in the relationship between transformational leadership and employee engagement. Economic and Industrial Democracy. 2015;39: 249–271. doi:10.1177/0143831X15614914

14. Boerner S, Dütschke E, Wied S. Charismatic leadership and organizational citizenship behaviour: examining the role of stressors and strain. Human Resource Development International. 2008;11: 507–521. doi:10.1080/13678860802417643

15. Bohorquez N. Perception of Leadership Styles, Organizational Commitment and Burnout in Faculty of Colombian Universities. ProQuest Dissertations and Theses. D.B.A., Northcentral University. 2016. Available: https://search.proquest.com/docview/1844411602/abstract/2013F228EF674643PQ/1

16. Bono JE, Foldes HJ, Vinson G, Muros JP. Workplace emotions: The role of supervision and leadership. Journal of Applied Psychology. 2007;92: 1357–1367. doi:10.1037/0021-9010.92.5.1357

17. Breevaart K, Bakker AB, Demerouti E, Sleebos DM, Maduro V. Uncovering the Underlying Relationship Between Transformational Leaders and Followers' Task Performance. Journal of Personnel Psychology. 2014;13: 194–203. doi:10.1027/1866-5888/a000118

18. Breevaart K, Bakker A, Hetland J, Demerouti E, Olsen OK, Espevik R. Daily transactional and transformational leadership and daily employee engagement. Journal of Occupational and Organizational Psychology. 2014;87: 138–157. doi:10.1111/joop.12041

19. Bright JB. The Relationship between Transformational Leadership Behaviors and Employee Engagement and Turnover Intent. Dissertation Abstracts International Section A: Humanities and Social Sciences. Vol.79(9-A(E)),2018, pp. No Pagination Specified. 2018.

20. Buck VB. The Impact of Transformational Leadership on Nonprofit Volunteer Engagement and Commitment. PhD Thesis, Walden University. 2018. Available: https://scholarworks.waldenu.edu/dissertations/5310/

21. Bui HTM, Zeng Y, Higgs M. The role of person-job fit in the relationship between transformational leadership and job engagement. Journal of Managerial Psych. 2017;32: 373–386. doi:10.1108/JMP-05-2016-0144

22. Buil I, Martínez E, Matute J. From internal brand management to organizational citizenship behaviours: Evidence from frontline employees in the hotel industry. Tourism Management. 2016;57: 256–271. doi:10.1016/j.tourman.2016.06.009

23. Caniëls MCJ, Semeijn JH, Renders IHM. Mind the mindset! The interaction of proactive personality, transformational leadership and growth mindset for engagement at work. Career Dev Int. 2018;23: 48–66. doi:10.1108/CDI-11-2016-0194

24. Carlström C, Johansson H. Transformativt och transaktionellt ledarskap och dess samband med medarbetarnas välbefinnande [Transformational and transactional leadership and their relationship with well-being]. Masters Thesis, Högskolan University. 2014. Available: https://www.diva-portal.org/smash/record.jsf?pid=diva2%3A711396&dswid=435

25. Cheng C, Bartram T, Karimi L, Leggat S. Transformational leadership and social identity as predictors of team climate, perceived quality of care, burnout and turnover intention among nurses. Personnel Review. 2016;45: 1200–1216. doi:10.1108/PR-05-2015-0118

26. Childers K. Teacher Perception of Principal Leadership and its Influence on Teacher Constructs: A Path Analysis Study. Ph.D., Northcentral University. 2019. Available: https://search.proquest.com/docview/2171103451/abstract/D6BD61BC4E4E49F8PQ/1

27. Chua J, Ayoko OB. Employees' self-determined motivation, transformational leadership and work engagement. Journal of Management & Organization. 2019; 1–21. doi:10.1017/jmo.2018.74

28. Cole MS, Bedeian AG. Leadership consensus as a cross-level contextual moderator of the emotional exhaustion–work commitment relationship. The Leadership Quarterly. 2007;18: 447–462. doi:10.1016/j.leaqua.2007.07.002

29. Corrigan PW, Diwan S, Campion J, Rashid F. Transformational Leadership and the Mental Health Team. Adm Policy Ment Health. 2002;30: 97–108. doi:10.1023/A:1022569617123

30. Daenzer BE. QUANTITATIVE CORRELATION OF LEADERSHIP STYLES AND JOB STRESS IN A MIDWEST UNITED STATES AUTO COMPANY. ProQuest Dissertation, University of Phoenix. 2009.

31. Dartey-Baah K, Yaw Ampofo E. Examining the Influence of Transformational and Transactional Leadership Styles on Perceived Job Stress among Ghanaian Banking Employees. IJBM. 2015;10: p161. doi:10.5539/ijbm.v10n8p161

32. Dibley J, Viviers R, van Zyl LE. A preliminary study: perceived transformational leadership and work engagement within the South African army. South African Journal of Labour relations. 2014;38: 16.

33. Diebig M, Bormann KC, Rowold J. Day-level transformational leadership and followers' daily level of stress: a moderated mediation model of team cooperation, role conflict, and type of communication. European Journal of Work and Organizational Psychology. 2017;26: 234–249. doi:10.1080/1359432X.2016.1250741

34. Diebig M, Poethke U, Rowold J. Leader strain and follower burnout: Exploring the role of transformational leadership behaviour - Mathias Diebig, Ute Poethke, Jens Rowold, 2017. German Journal of Human Resource Management. 2017;31: 329–348. doi:https://doi.org/10.1177/2397002217721077

35. Divya S, Suganthi L. Influence of transformational-servant leadership styles and justice perceptions on employee burnout: a moderated mediation model. International Journal of Business Innovation and Research. 2018;15: 119–135.

36. Dubinsky AJ, Yammarino FJ, Jolson MA, Spangler WD. Transformational Leadership: An Initial Investigation in Sales Management. Journal of Personal Selling & Sales Management. 2013;15: 17–31.

37. Dunkl A, Jiménez P, Šarotar Žižek S, Milfelner B, Kallus WK. Similarities and Differences of Health-promoting Leadership and Transformational Leadership. Naše gospodarstvo/Our economy. 2015;61: 3–13. doi:10.1515/ngoe-2015-0013

38. Eghdamy H, Ganjiniya H, Akhlagh EM. Relationship between Transformational Leadership and Reduction of Burnout among Employees of Greater Tehran Foundation of Martyrs and Veterans Affairs. KCAJBMR. 2013;3: 34–39. doi:10.12816/0018157

39. Enwereuzor IK, Ugwu LI, Eze OA. How Transformational Leadership Influences Work Engagement Among Nurses: Does Person–Job Fit Matter? West J Nurs Res. 2018;40: 346–366. doi:10.1177/0193945916682449

40. Espinoza-Parra S, Molero F, Fuster-Ruizdeapodaca MJ. Transformational leadership and job satisfaction of police officers ( *carabineros* ) in Chile: the mediating effects of group identification

and work engagement / Liderazgo transformacional y satisfacción laboral en carabineros de Chile: los efectos mediadores de la identificación con el grupo y el work engagement. Revista de Psicología Social. 2015;30: 439–467. doi:10.1080/02134748.2015.1065087

41. Eyal O, Roth G. Principals' leadership and teachers' motivation: Self‐determination theory analysis. Journal of Educational Admin. 2011;49: 256–275. doi:10.1108/09578231111129055

42. Faupel S, Süß S. The Effect of Transformational Leadership on Employees During Organizational Change – An Empirical Analysis. Journal of Change Management. 2019;19: 145–166. doi:10.1080/14697017.2018.1447006

43. Felfe J, Franke F, Liepmann D. Scale documentation. Unpublished manuscript of the Helmut-Schmidt-Universität Hamburg and the Freie Universität Berlin. Hamburg, Germany; 2012.

44. Felfe J, Schyns B. Is Similarity in Leadership Related to Organizational Outcomes? The Case of Transformational Leadership. Journal of Leadership & Organizational Studies. 2004;10: 92–102. doi:10.1177/107179190401000407

45. Fernet C, Trépanier S-G, Austin S, Gagné M, Forest J. Transformational leadership and optimal functioning at work: On the mediating role of employees' perceived job characteristics and motivation. Work & Stress. 2015;29: 11–31. doi:10.1080/02678373.2014.1003998

46. Franke F. Leadership and follower health: The effects of transformational and health-oriented leadership on follower health outcomes. unpublished dissertation, Helmut-Schmidt-Universität Hamburg. 2012.

47. Franke F, Felfe J, Pundt A. The Impact of Health-Oriented Leadership on Follower Health: Development and Test of a New Instrument Measuring Health-Promoting Leadership. German Journal of Human Resource Management: Zeitschrift für Personalforschung. 2014;28: 139–161. doi:10.1177/239700221402800108

48. Franke F, Felfe J. How does transformational leadership impact employees' psychological strain?: Examining differentiated effects and the moderating role of affective organizational commitment. Leadership. 2011;7: 295–316. doi:10.1177/1742715011407387

49. Freeborough R, Patterson K. Exploring the Effect of Transformational Leadership on Nonprofit Leader Engagement. Servant Leadership: Theory and Practice. 2015;2: 49–70.

50. Garcia-Rivera BR, Alejandro Mendoza-Martinez IA, Ramirez-Baron MC. The Impact Of Transformational Leadership In Burnout Of Nurses And Doctors In A Public Hospital Of Mexico. AJHS. 2012;4: 1–12. doi:10.19030/ajhs.v4i1.7592

51. García-Sierra R, Fernández-Castro J. Relationships between leadership, structural empowerment, and engagement in nurses. J Adv Nurs. 2018;74: 2809–2819. doi:10.1111/jan.13805

52. Gautam A, Enslin E. Transformational Leadership and Work Engagement in the Automotive Retail Industry: A Study of South Africa. IBR. 2019;12: 133. doi:10.5539/ibr.v12n5p133

53. Gerards R, de Grip A, Baudewijns C. Do new ways of working increase work engagement? Personnel Review. 2018;47: 517–534. doi:10.1108/PR-02-2017-0050

54. Gerick J. Transformationale Führung aus Lehrersicht und ihre Bedeutung für die Lehrergesundheit. In: Drossel K, Strietholt R, Bos W, editors. Empirische Bildungsforschung

und evidenzbasierte Reformen im Bildungswesen. Münster, Germany: Waxmann Verlag GmbH; 2014. pp. 109–127.

55. Ghadi M, Fernando M, Caputi P. Transformational leadership and work engagement: The mediating effect of meaning in work. Leadership & Org Development J. 2013;34: 532–550. doi:10.1108/LODJ-10-2011-0110

56. Ghislieri C, Gatti P, Tartari M, Cortese CG. Quale relazione tra leadership e work engagement? Uno studio pilota in una pubblica amministrazione [Investigating the relationship between leadership and work engagement: A pilot study in a public administration environment]. Counseling. 2013;6: 61–73.

57. Gilbert M-H, Dagenais-Desmarais V, St-Hilaire F. Transformational leadership and autonomy support management behaviors: The role of specificity in predicting employees' psychological health. Leadership & Org Development J. 2017;38: 320–332. doi:10.1108/LODJ-08-2015-0173

58. Gilbert SL, Kelloway EK. Leadership, Recognition and Well-Being: A Moderated Mediational Model. Can J Adm Sci. 2018;35: 523–534. doi:10.1002/cjas.1477

59. Gill A, Flaschner AB, Bhutani S. The Impact of Transformational Leadership and Empowerment on Employee Job Stress. Business and Economics Journal. 2010;2010: BEJ–3: 12.

60. Gill AS, Flaschner AB, Shachar M. Mitigating stress and burnout by implementing transformational-leadership. International Journal of Contemporary Hospitality Management. 2006;18: 469–481. doi:10.1108/09596110610681511

61. Gillet N, Fouquereau E, Bonnaud-Antignac A, Mokounkolo R, Colombat P. The mediating role of organizational justice in the relationship between transformational leadership and nurses' quality of work life: A cross-sectional questionnaire survey. International Journal of Nursing Studies. 2013;50: 1359–1367. doi:10.1016/j.ijnurstu.2012.12.012

62. Gong T, Zimmerli L, Hoffer HE. The effects of transformational leadership and the sense of calling on job burnout among special education teachers. Journal of School Leadership. 2013;23: 969–994.

63. Gordick DH. Employee dispositional characteristics and supervisor leadership behavior as predictors of employee coping with organizational change. Georgia State University. 2002.

64. Goswami A, Nair P, Beehr T, Grossenbacher M. The relationship of leaders' humor and employees' work engagement mediated by positive emotions: Moderating effect of leaders' transformational leadership style. Leadership & Org Development J. 2016;37: 1083–1099. doi:10.1108/LODJ-01-2015-0001

65. Green AE, Albanese BJ, Shapiro NM, Aarons GA. The roles of individual and organizational factors in burnout among community-based mental health service providers. Psychological Services. 2014;11: 41–49. doi:10.1037/a0035299

66. Green AE, Miller EA, Aarons GA. Transformational Leadership Moderates the Relationship Between Emotional Exhaustion and Turnover Intention Among Community Mental Health Providers. Community Ment Health J. 2013;49: 373–379. doi:10.1007/s10597-011-9463-0

67. Gregersen S, Vincent-Höper S, Nienhaus A. Health-Relevant Leadership Behaviour: A Comparison of Leadership Constructs. German Journal of Human Resource Management: Zeitschrift für Personalforschung. 2014;28: 117–138. doi:10.1177/239700221402800107

68. Gregersen S, Vincent-Höper S, Nienhaus A. The Relation Between Leadership and Perceived Well-Being: What Role Does Occupational Self-Efficacy Play? J Ldrship Studies. 2014;8: 6–18. doi:10.1002/jls.21318

69. Gutierrez AM. An Exploration of the Effects of Psychological Climate, Workplace Friendships, and Psychological Needs on Perceived Transformational Leadership and Work Engagement. Dissertation Abstracts International: Section B: The Sciences and Engineering. Vol.76(2-B(E)),2015, pp. No Pagination Specified., Alliant International University. 2014.

70. Hand LE, Hicks RE, Bahr M. Relationships among transformational and transactional leadership styles, role pressures, stress levels, and coping resources in senior Queensland catholic education executives. 2015. Available: https://research.bond.edu.au/en/publications/relationships-among-transformational-and-transactional-leadership

71. Hansen A, Byrne Z, Kiersch C. How interpersonal leadership relates to employee engagement. Journal of Managerial Psych. 2014;29: 953–972. doi:10.1108/JMP-11-2012-0343

72. Hartling NC. The LEAD program and the Effect of Leadership on Employee Well-Being. Saint Mary's University. 2019. Available: http://t.library2.smu.ca/bitstream/handle/01/29134/Hartling_Nikola_PHD_2019.pdf?sequence=1&isAllowed=y

73. Hawkes AJ, Biggs A, Hegerty E. Work Engagement: Investigating the Role of Transformational Leadership, Job Resources, and Recovery. The Journal of Psychology. 2017;151: 509–531. doi:10.1080/00223980.2017.1372339

74. Hayati D, Charkhabi M, Naami A. The relationship between transformational leadership and work engagement in governmental hospitals nurses: a survey study. SpringerPlus. 2014;3: 25. doi:10.1186/2193-1801-3-25

75. Hee CO, Ibrahim R, Owee Kowan T, Chin Fei G. Employee Engagement as a Mediator Between Transformational Leadership and Employee Performance. Asian J of Scientific Research. 2018;11: 441–448. doi:10.3923/ajsr.2018.441.448

76. Henkel TG. The relationship between transformational leadership styles and university adjunct faculty work engagement. Northcentral University. 2012. Available: file:///Users/Fri/Downloads/The%20Relationship%20Between%20Transformational%20Leadership%20Styles%20and%20U.pdf

77. Hentrich S, Zimber A, Garbade SF, Gregersen S, Nienhaus A, Petermann F. Relationships between transformational leadership and health: The mediating role of perceived job demands and occupational self-efficacy. International Journal of Stress Management. 2017;24: 34–61. doi:10.1037/str0000027

78. Hetland H, Sandal GM, Johnsen TB. Burnout in the information technology sector: Does leadership matter? European Journal of Work and Organizational Psychology. 2007;16: 58–75. doi:10.1080/13594320601084558

79. Hildenbrand K, Sacramento CA, Binnewies C. Transformational leadership and burnout: The role of thriving and followers' openness to experience. Journal of Occupational Health Psychology. 2018;23: 31–43. doi:10.1037/ocp0000051

80. Holstad TJ, Korek S, Rigotti T, Mohr G. The relation between transformational leadership and follower emotional strain: The moderating role of professional ambition. Leadership. 2014;10: 269–288. doi:10.1177/1742715013476083

81. Holstad TJ, Rigotti T, Otto K. Prozedurale Fairness als Mediator zwischen transformationaler Führung und psychischer Beanspruchung am Arbeitsplatz: Eine Mehrebenenstudie. Zeitschrift für Arbeits- und Organisationspsychologie A&O. 2013;57: 163–176. doi:10.1026/0932-4089/a000120

82. Holten A-L, Bøllingtoft A, Carneiro IG, Borg V. A within-country study of leadership perceptions and outcomes across native and immigrant employees: Questioning the universality of transformational leadership. Journal of Management & Organization. 2018;24: 145–162. doi:10.1017/jmo.2017.2

83. Ilyavi Y. Correlational Study of Principal Leadership Styles and Teacher's Emotional Exhaustion, Depersonalization, and Feeling of Reduced Personal Accomplishment. Ph.D. 2019. Available: https://search.proquest.com/docview/2209973722/6B7771FD97D24120PQ/1

84. Jacobs C, Pfaff H, Lehner B, Driller E, Nitzsche A, Stieler-Lorenz B, et al. The Influence of Transformational Leadership on Employee Well-Being: Results From a Survey of Companies in the Information and Communication Technology Sector in Germany. Journal of Occupational and Environmental Medicine. 2013;55: 772–778. doi:10.1097/JOM.0b013e3182972ee5

85. Jansen in de Wal J, van den Beemt A, Martens RL, den Brok PJ. The relationship between job demands, job resources and teachers' professional learning: is it explained by self-determination theory? Studies in Continuing Education. 2020;42: 17–39. doi:10.1080/0158037X.2018.1520697

86. Javeed T, Farooqi YA. Impact of Transformational Leadership Style on Employees' Satisfaction and Well-Being with Working Conditions as Mediator. International Journal of multidisciplinary sciences and engineering. 2013;4: 8.

87. Jena LK, Pradhan S, Panigrahy NP. Pursuit of organisational trust: Role of employee engagement, psychological well-being and transformational leadership. Asia Pacific Management Review. 2018;23: 227–234. doi:10.1016/j.apmrv.2017.11.001

88. Johnston PJ. Transformational leadership and employee well-being; a longitudinal two-study investigation. Saint Mary's University. 2012. Available: http://www.springerlink.com.library2.smu.ca/handle/01/24841#.X18ZQtbgpp8

89. Jordan DE. Examining the relationship between perceived leadership behaviors and employee engagement among part-time retail employees. Ph.D. 2016. Available: https://search.proquest.com/docview/1810727801/abstract/D5360E090D954D4APQ/1

90. Kadian R, Ahlawat P. Role of Empowerment, Transformational Leadership and Social Support in Influencing Sales People Burnout. International Journal of Research. 2017; 14.

91. Kanste O, Kyngäs H, Nikkilä J. The relationship between multidimensional leadership and burnout among nursing staff. J Nurs Manag. 2007;15: 731–739. doi:10.1111/j.1365-2934.2006.00741.x

92. Kara D, Uysal M, Sirgy MJ, Lee G. The effects of leadership style on employee well-being in hospitality. International Journal of Hospitality Management. 2013;34: 9–18. doi:10.1016/j.ijhm.2013.02.001

93. Kath LM, Stichler JF, Ehrhart MG, Schultze TA. Predictors and Outcomes of Nurse Leader Job Stress Experienced by AWHONN Members. Journal of Obstetric, Gynecologic & Neonatal Nursing. 2013;42: E12–E25. doi:10.1111/j.1552-6909.2012.01430.x

94. Kath LM, Stichler JF, Ehrhart MG, Sievers A. Predictors of nurse manager stress: A dominance analysis of potential work environment stressors. International Journal of Nursing Studies. 2013;50: 1474–1480. doi:10.1016/j.ijnurstu.2013.02.011

95. Kelloway EK, Turner N, Barling J, Loughlin C. Transformational leadership and employee psychological well-being: The mediating role of employee trust in leadership. Work & Stress. 2012;26: 39–55. doi:10.1080/02678373.2012.660774

96. Kelloway EK, Weigand H, McKee MC, Das H. Positive Leadership and Employee Well-Being. Journal of Leadership & Organizational Studies. 2013;20: 107–117. doi:10.1177/1548051812465892

97. Kensbock JM, Boehm SA. The role of transformational leadership in the mental health and job performance of employees with disabilities. The International Journal of Human Resource Management. 2016;27: 1580–1609. doi:10.1080/09585192.2015.1079231

98. Klaic A, Burtscher MJ, Jonas K. Person-supervisor fit, needs-supplies fit, and team fit as mediators of the relationship between dual-focused transformational leadership and well-being in scientific teams. European Journal of Work and Organizational Psychology. 2018;27: 669–682. doi:10.1080/1359432X.2018.1502174

99. Kranabetter C, Niessen C. Managers as role models for health: Moderators of the relationship of transformational leadership with employee exhaustion and cynicism. Journal of Occupational Health Psychology. 2017;22: 492–502. doi:10.1037/ocp0000044

100. Lam CS, O'Higgins ERE. Enhancing employee outcomes. Leadership & Organization Development Journal. 2012;33: 149–174. doi:10.1108/01437731211203465

101. Lamade MJ. The Impact of Leadership Style on Work Engagement in the United States Volunteer Fire Service. Ph.D. 2019. Available: https://search.proquest.com/docview/2312908295/abstract/D6A86855E5CB48BEPQ/1

102. LaRocca MA, Scogin FR, Hilgeman MM, Smith AJ, Chaplin WF. The impact of posttraumatic growth, transformational leadership, and self-efficacy on PTSD and depression symptom severity among combat Veterans. Military Psychology. 2018;30: 162–173. doi:10.1080/08995605.2018.1425073

103. Laukamp LH. Good, better, best! - Führungsstile und wirtschaftliche Leistung. Westfälische Wilhelms-Universität Münster. 2008.

104. Lee H, Cummings GG. Examining relationships between director leadership practices and manager worklife and burnout. J Ldrship Studies. 2008;2: 47–62. doi:10.1002/jls.20061

105. Lee M-Y, Kim K-H. Influence of head nurses' transformational leadership on staff nurse's psychological well-being, stress and somatization-focused on the mediating effect of positive psychological capital. Journal of Korean Academy of Nursing Administration. 2012;18: 166–175.

106. Lee MCC, Idris MohdA, Tuckey M. Supervisory coaching and performance feedback as mediators of the relationships between leadership styles, work engagement, and turnover

intention. Human Resource Development International. 2019;22: 257–282. doi:10.1080/13678868.2018.1530170

107. Lewis HS, Cunningham CJL. Linking Nurse Leadership and Work Characteristics to Nurse Burnout and Engagement: Nursing Research. 2016;65: 13–23. doi:10.1097/NNR.0000000000000130

108. Li H, Sajjad N, Wang Q, Muhammad Ali A, Khaqan Z, Amina S. Influence of Transformational Leadership on Employees' Innovative Work Behavior in Sustainable Organizations: Test of Mediation and Moderation Processes. Sustainability. 2019;11: 1594. doi:10.3390/su11061594

109. Li Y. Leadership styles and knowledge workers' work engagement: Psychological capital as a mediator. Curr Psychol. 2019;38: 1152–1161. doi:10.1007/s12144-018-9968-6

110. Liang T-L, Chang H-F, Ko M-H, Lin C-W. Transformational leadership and employee voices in the hospitality industry. Int J Contemp Hospitality Mngt. 2017;29: 374–392. doi:10.1108/IJCHM-07-2015-0364

111. Lin P-Y, MacLennan S, Hunt N, Cox T. The influences of nursing transformational leadership style on the quality of nurses' working lives in Taiwan: a cross-sectional quantitative study. BMC Nurs. 2015;14: 33. doi:10.1186/s12912-015-0082-x

112. Lin S-H (Joanna), Scott BA, Matta FK. The Dark Side of Transformational Leader Behaviors for Leaders Themselves: A Conservation of Resources Perspective. AMJ. 2019;62: 1556–1582. doi:10.5465/amj.2016.1255

113. Liu C, Liu S, Yang S, Wu H. Association between transformational leadership and occupational burnout and the mediating effects of psychological empowerment in this relationship among CDC employees: a cross-sectional study. PRBM. 2019;Volume 12: 437–446. doi:10.2147/PRBM.S206636

114. Liu J, Siu O-L, Shi K. Transformational Leadership and Employee Well-Being: The Mediating Role of Trust in the Leader and Self-Efficacy: TRANSFORMATIONAL LEADERSHIP. Applied Psychology: an International Review. 2009;59: 454–479. doi:10.1111/j.1464-0597.2009.00407.x

115. Liu WK, Lee Y. Assessment of Cultural Dimensions, Leadership Behaviors and Leadership Self-Efficacy: Examination of Multinational Corporations in Taiwan. IPEDR, Vol 28. Singapore: IACSIT Press; 2012. p. 6.

116. Liu Y. LINKING AUTHENTIC LEADERSHIP TO POSITIVE EMPLOYEE HEALTH, BEHAVIORAL ENGAGEMENT, AND JOB PERFORMANCE. Louisiana Tech University. 2012. Available: https://search.proquest.com/openview/93a8501140b2cebd72586b08aa34d4d7/1?pq-origsite=gscholar&cbl=18750&diss=y

117. Lozano-Chapa MM. An Investigation of the Relationship between Counselor's Ratings of Their Principal's Leadership Style and Counselor Burnout in the Public-School System. 2017. p. 108. Available: https://search.proquest.com/docview/2034281417/6C2A7D4335C74A99PQ/1

118. Madathil R, Heck NC, Schuldberg D. Burnout in Psychiatric Nursing: Examining the Interplay of Autonomy, Leadership Style, and Depressive Symptoms. Archives of Psychiatric Nursing. 2014;28: 160–166. doi:10.1016/j.apnu.2014.01.002

119. Malloy T, Penprase B. Nursing leadership style and psychosocial work environment: Leadership style and work environment. Journal of Nursing Management. 2010;18: 715–725. doi:10.1111/j.1365-2834.2010.01094.x

120. Maricle WH. SCHOOL ADMINISTRATOR SELF-PERCEIVED LEADERSHIP STYLES AFFECT ON OCCUPATIONAL BURNOUT. ProQuest Dissertation, Lamar University. 2013. Available: https://search.proquest.com/openview/3c579ec19461372d01ea6ebaa6f8d645/1?pq-origsite=gscholar&cbl=18750&diss=y

121. Mauno S, Ruokolainen M, Kinnunen U, De Bloom J. Emotional labour and work engagement among nurses: examining perceived compassion, leadership and work ethic as stress buffers. J Adv Nurs. 2016;72: 1169–1181. doi:10.1111/jan.12906

122. Mayr ML. Transformational Leadership and Volunteer Firefighter Engagement: The Mediating Role of Group Identification and Perceived Social Impact. Nonprofit Management and Leadership. 2017;28: 259–270. doi:10.1002/nml.21279

123. Mazzetti G, Vignoli M, Petruzziello G, Palareti L. The Hardier You Are, the Healthier You Become. May Hardiness and Engagement Explain the Relationship Between Leadership and Employees' Health? Front Psychol. 2019;9: 2784. doi:10.3389/fpsyg.2018.02784

124. McKee MC, Driscoll C, Kelloway EK, Kelley E. Exploring linkages among transformational leadership, workplace spirituality and well-being in health care workers. Journal of Management, Spirituality & Religion. 2011;8: 233–255. doi:10.1080/14766086.2011.599147

125. Mehari B. Transformational leadership and well-being: The mediating role of trust in leadership, meaningfulness and job satisfaction. Masters Thesis, Linnaeus University. 2015. Available: http://rgdoi.net/10.13140/RG.2.1.1362.4489

126. Monje Amor A, Abeal Vázquez JP, Faíña JA. Transformational leadership and work engagement: Exploring the mediating role of structural empowerment. European Management Journal. 2020;38: 169–178. doi:10.1016/j.emj.2019.06.007

127. Moss S. Cultivating the Regulatory Focus of Followers to Amplify Their Sensitivity to Transformational Leadership. Journal of Leadership & Organizational Studies. 2009;15: 241–259. doi:10.1177/1548051808327880

128. Mostafa AMS. Transformational leadership and restaurant employees customer-oriented behaviours: The mediating role of organizational social capital and work engagement. Int J Contemp Hospitality Mngt. 2019;31: 1166–1182. doi:10.1108/IJCHM-02-2018-0123

129. Munir F, Nielsen K, Carneiro GI. Transformational leadership and depressive symptoms: A prospective study. Journal of Affective Disorders. 2010;120: 235–239. doi:10.1016/j.jad.2009.03.020

130. Nelson SA, Shraim O. Leadership behaviour and employee engagement: a Kuwaiti services company. IJHRDM. 2014;14: 119. doi:10.1504/IJHRDM.2014.068078

131. Nielsen K, Daniels K. Does shared and differentiated transformational leadership predict followers' working conditions and well-being? The Leadership Quarterly. 2012;23: 383–397. doi:10.1016/j.leaqua.2011.09.001

132. Niessen C, Mäder I, Stride C, Jimmieson NL. Thriving when exhausted: The role of perceived transformational leadership. Journal of Vocational Behavior. 2017;103: 41–51. doi:10.1016/j.jvb.2017.07.012

133. Odink N. Three Dimensions of Employee Well-Being: Happiness, Health and Relationships. Masters Thesis, Tilburg University. 2014.

134. Othman AK, Hamzah MI, Abas MK, Zakuan NM. The influence of leadership styles on employee engagement: The moderating effect of communication styles. Int j adv appl sci. 2017;4: 107–116. doi:10.21833/ijaas.2017.03.017

135. Parveen M, Adeinat I. Transformational leadership: does it really decrease work-related stress? LODJ. 2019;40: 860–876. doi:10.1108/LODJ-01-2019-0023

136. Peng J-C, Tseng M-M. Antecedent and Consequence of Nurse Engagement. The Journal of Psychology. 2019;153: 342–359. doi:10.1080/00223980.2018.1536639

137. Perilla-Toro LE, Gómez-Ortiz V. Relación del estilo de liderazgo transformacional con la salud y el bienestar del empleado: el rol mediador de la confianza en el líder. Revista de Psicología del Trabajo y de las Organizaciones. 2017;33: 95–108. doi:10.1016/j.rpto.2017.02.005

138. Perko K, Kinnunen U, Feldt T. Transformational leadership and depressive symptoms among employees: mediating factors. Leadership & Org Development J. 2014;35: 286–304. doi:10.1108/LODJ-07-2012-0082

139. Perko K, Kinnunen U, Tolvanen A, Feldt T. Investigating occupational well-being and leadership from a person-centred longitudinal approach: congruence of well-being and perceived leadership. European Journal of Work and Organizational Psychology. 2016;25: 105–119. doi:10.1080/1359432X.2015.1011136

140. Prochazka J, Gilova H, Vaculik M. The Relationship Between Transformational Leadership and Engagement: Self-Efficacy as a Mediator. J Ldrship Studies. 2017;11: 22–33. doi:10.1002/jls.21518

141. Reddy AV, Mehta HN. Mediating role of transformational leadership on the relationship between burnout and intention to quit among the employees of select hotels in South India. Organizational Psychology. 2019;9: 8–17.

142. Rittschof KR, Fortunato VJ. The Influence of Transformational Leadership and Job Burnout on Child Protective Services Case Managers' Commitment and Intent to Quit. Journal of Social Service Research. 2016;42: 372–385. doi:10.1080/01488376.2015.1101047

143. Rowold J, Heinitz K. Führungsstile als Stressbarrieren: Zum Zusammenhang zwischen transformationaler, transaktionaler, mitarbeiter- und aufgabenorientierter Führung und Indikatoren von Stress bei Mitarbeitern. Zeitschrift für Personalpsychologie. 2008;7: 129–140. doi:10.1026/1617-6391.7.3.129

144. Russell LM, Cole BM, Jones RJ. High-risk Occupations: How Leadership, Stress, and Ability to Cope Influence Burnout in Law Enforcement. Journal of Leadership, Accountability and Ethics. 2014;11: 49–69.

145. Şahin D, Çubuk D, Uslu T. The Effect of Organizational Support, Transformational Leadership, Personnel Empowerment, Work Engagement, Performance and Demographical Variables on the Factors of Psychological Capital. emaj. 2014;3: 1–17. doi:10.5195/EMAJ.2014.49

146. Sahu S, Pathardikar A, Kumar A. Transformational leadership and turnover: Mediating effects of employee engagement, employer branding, and psychological attachment. Leadership & Org Development J. 2018;39: 82–99. doi:10.1108/LODJ-12-2014-0243

147. Salanova M, Del Líbano M, Llorens S, Schaufeli WB. Engaged, Workaholic, Burned-Out or Just 9-to-5? Toward a Typology of Employee Well-being: Employee Well-being and Work Investment. Stress and Health. 2014;30: 71–81. doi:10.1002/smi.2499

148. Salanova M, Lorente L, Chambel MJ, Martínez IM. Linking transformational leadership to nurses' extra-role performance: the mediating role of self-efficacy and work engagement: Linking transformational leadership to nurses' extra-role performance. Journal of Advanced Nursing. 2011;67: 2256–2266. doi:10.1111/j.1365-2648.2011.05652.x

149. Salem IE-B. Transformational leadership: Relationship to job stress and job burnout in five-star hotels. Tourism and Hospitality Research. 2015;15: 240–253. doi:10.1177/1467358415581445

150. Schmitt A, Den Hartog DN, Belschak FD. Transformational leadership and proactive work behaviour: A moderated mediation model including work engagement and job strain. J Occup Organ Psychol. 2016;89: 588–610. doi:10.1111/joop.12143

151. Seltzer J, Numerof RitaE, Bass BM. Transformational leadership: is it a source of more burnout and stress? Journal of Health and Human Resources Administration. 1989;12: 174–185.

152. Sharma D, Krishnan VR. The Impact of Pay Satisfaction and Transformational Leadership on Employee Engagement. Proceedings of the 5th International Conference of Management and Behavioural Sciences. Haridwar, India; 2012. p. 13. Available: file:///Users/Fri/Downloads/devyani.pdf

153. Shi R, Zhang S, Xu H, Liu X, Miao D. Regulatory focus and burnout in nurses: The mediating effect of perception of transformational leadership: Regulatory focus, leadership, and burnout. Int J Nurs Pract. 2015;21: 858–867. doi:10.1111/ijn.12315

154. Shokory SM, Suradi NRM. Transformational Leadership and its Impact on Extra-Role Performance of Project Team Members: The Mediating Role of Work Engagement. Academy of Strategic Management Journal. 2018 [cited 27 Apr 2020]. Available: https://www.abacademies.org/abstract/transformational-leadership-and-its-impact-on-extrarole-performance-of-project-team-members-the-mediating-role-of-work-e-7605.html

155. Skogstad A, Einarsen S, Torsheim T, Aasland MS, Hetland H. The destructiveness of laissez-faire leadership behavior. Journal of Occupational Health Psychology. 2007;12: 80–92. doi:10.1037/1076-8998.12.1.80

156. Smith HK. Testing work characteristics as mediating factors in the relationships among nurse leadership, burnout, and engagement. Masters Thesis, University of Tennessee. 2012.

157. Song HJ, Kolb JA, Hee Lee U, Kyoung Kim H. Role of transformational leadership in effective organizational knowledge creation practices: Mediating effects of employees' work engagement. Human Resource Development Quarterly. 2012;23: 65–101. doi:10.1002/hrdq.21120

158. Stein M, Schümann M, Vincent-Höper S. A Conservation of Resources View of the Relationship Between Transformational Leadership and Emotional Exhaustion: The Role of Extra Effort and Psychological Detachment. Work & Stress. in press.

159. Stein M, Vincent-Höper S, Deci N, Gregersen S, Nienhaus A. Compensatory Coping Through the Extension of Working Hours: The Buffering Role of Leader–Member Exchange. Zeitschrift für Arbeits- und Organisationspsychologie A&O. 2019;63: 115–128. doi:10.1026/0932-4089/a000285

160. Stenling A, Tafvelin S. Transformational Leadership and Well-Being in Sports: The Mediating Role of Need Satisfaction. Journal of Applied Sport Psychology. 2014;26: 182–196. doi:10.1080/10413200.2013.819392

161. Stordeur S, D'Hoore W, Vandenberghe C. Leadership, organizational stress, and emotional exhaustion among hospital nursing staff. J Adv Nurs. 2001;35: 533–542. doi:10.1046/j.1365-2648.2001.01885.x

162. Strom DL, Sears KL, Kelly KM. Work Engagement: The Roles of Organizational Justice and Leadership Style in Predicting Engagement Among Employees. Journal of Leadership & Organizational Studies. 2014;21: 71–82. doi:10.1177/1548051813485437

163. Syrek CJ, Apostel E, Antoni CH. Stress in highly demanding IT jobs: Transformational leadership moderates the impact of time pressure on exhaustion and work–life balance. Journal of Occupational Health Psychology. 2013;18: 252–261. doi:10.1037/a0033085

164. Tafvelin S, Armelius K, Westerberg K. Toward Understanding the Direct and Indirect Effects of Transformational Leadership on Well-Being : A Longitudinal Study. Journal of Leadership & Organizational Studies. 2011;18: 480–492. doi:https://doi.org/10.1177/1548051811418342

165. Tafvelin S, Nielsen K, von Thiele Schwarz U, Stenling A. Leading well is a matter of resources: Leader vigour and peer support augments the relationship between transformational leadership and burnout. Work & Stress. 2019;33: 156–172. doi:10.1080/02678373.2018.1513961

166. Taylor TM. The relationship between transformation leadership and staff and faculty work engagement. Dissertation Abstracts International Section A: Humanities and Social Sciences. Vol.75(7-A(E)),2015, pp. No Pagination Specified., Northcentral University. 2015.

167. Teare R. International Journal of Contemporary Hospitality Management. Mrkting Intelligence & Plan. 1990;8: 12–14. doi:10.1108/EUM0000000001079

168. Tims M, Bakker AB, Xanthopoulou D. Do transformational leaders enhance their followers' daily work engagement? The Leadership Quarterly. 2011;22: 121–131. doi:10.1016/j.leaqua.2010.12.011

169. Tsai W-C, Chen H-W, Cheng J-W. Employee positive moods as a mediator linking transformational leadership and employee work outcomes. The International Journal of Human Resource Management. 2009;20: 206–219. doi:10.1080/09585190802528714

170. Tse HHM. The effects of leadership styles on individuals' time use orientation, well-being and effectiveness. Masters Thesis, Hong Kong Polytechnic University. 2002.

171. van Dick R, Lemoine JE, Steffens NK, Kerschreiter R, Akfirat SA, Avanzi L, et al. Identity leadership going global: Validation of the Identity Leadership Inventory across 20 countries. J Occup Organ Psychol. 2018;91: 697–728. doi:10.1111/joop.12223

172. van Dierendonck D, Stam D, Boersma P, de Windt N, Alkema J. Same difference? Exploring the differential mechanisms linking servant leadership and transformational leadership to follower outcomes. The Leadership Quarterly. 2014;25: 544–562. doi:10.1016/j.leaqua.2013.11.014

173. Velkova GG. AN EXAMINATION OF LEADERSHIP STYLES: THEIR RELATIONSHIP TO WELL-BEING AMONG EMPLOYEES IN A MANUFACTURING ENVIRONMENT. Capella University. 2011.

174. Verbraak K. Transformational leadership and employee well-being The role of trust in the leader. Masters Thesis, Tilburg University. 2014.

175. Vignoli M, Depolo M, Cifuentes M, Punnett L. Disagreements on leadership styles between supervisors and employees are related to employees' well-being and work team outcomes. Intl J of Workplace Health Mgt. 2018;11: 274–293. doi:10.1108/IJWHM-11-2016-0084

176. Vila-Vázquez G, Castro-Casal C, Álvarez-Pérez D, del Río-Araújo L. Promoting the Sustainability of Organizations: Contribution of Transformational Leadership to Job Engagement. Sustainability. 2018;10: 4109. doi:10.3390/su10114109

177. Vincent-Höper S. Transformational leadership and employee well-being. Unpublished data. Stellenbosh, South Africa; 2013.

178. Vincent-Höper S, Gregersen S, Nienhaus A. Do Work Characteristics Mediate the Negative Effect of Transformational Leadership on Impaired Well-Being? Zeitschrift für Arbeits- und Organisationspsychologie A&O. 2017;61: 167–180. doi:10.1026/0932-4089/a000249

179. Vincent-Höper S, Muser C, Janneck M. Transformational leadership, work engagement, and occupational success. Career Development International. 2012;17: 663–682. doi:10.1108/13620431211283805

180. Vincent-Höper S, Stein M. The Role of Leaders in Designing Employees' Work Characteristics: Validation of the Health- and Development-Promoting Leadership Behavior Questionnaire. Front Psychol. 2019;10: 1049. doi:10.3389/fpsyg.2019.01049

181. Vincent-Höper S, Stein M. Transformational leadership and employee well-being. Unpublished data. 2020.

182. Vincent-Höper S, Stein M, Gregersen S, Nienhaus A. Messung gesundheitsförderlicher Führung in Gesundheitsdienst und Wohlfahrtspflege. Arbeitsmedizin, Sozialmedizin, Umweltmedizin. 2018;53: 46–53.

183. Walsh M, Dupre K, Arnold KA. Processes through Which Transformational Leaders Affect Employee Psychological Health. German Journal of Human Resource Management: Zeitschrift für Personalforschung. 2014;28: 162–172. doi:10.1177/239700221402800109

184. Walsh MM, Arnold KA. The bright and dark sides of employee mindfulness: Leadership style and employee well-being. Stress Health. 2020 [cited 22 Apr 2020]. doi:10.1002/smi.2926

185. Wang Z, Li C, Li X. Resilience, Leadership and Work Engagement: The Mediating Role of Positive Affect. Soc Indic Res. 2017;132: 699–708. doi:10.1007/s11205-016-1306-5

186. Wefald AJ, Reichard RJ, Serrano SA. Fitting Engagement Into a Nomological Network: The Relationship of Engagement to Leadership and Personality. Journal of Leadership & Organizational Studies. 2011;18: 522–537. doi:10.1177/1548051811404890

187. Whitford T, Moss SA. Transformational Leadership in Distributed Work Groups: The Moderating Role of Follower Regulatory Focus and Goal Orientation. Communication Research. 2009;36: 810–837. doi:10.1177/0093650209346800

188. Widmann BS. INFLUENCE OF LEADERSHIP STYLE ON WORK ENGAGEMENT OF KNOWLEDGE WORKERS IN AN ENGINEERING ORGANIZATION. Indiana Wesleyan University. 2013.

189. Wolf S. Führung und Gesundheit im Längsschnitt: Eine Cross-Lagged-Panel-Analyse zur Wirkung transformationaler Führung auf die Entwicklung und den Abbau von Mitarbeiterressourcen. [leadership and well-being in a longitudinal investigation: a cross-lagged-panel-analysis of the effect of transformational leadership on the development and the dissamination of follower resources. dissertation, TU Dresden. 2012.

190. Wolf S, Nebel-Töpfer C, Richter P. Burnout components as predictors of work engagement, psychological well-being and physical health. Unpublished article. 2007.

191. Wolf S, Nebel-Töpfer C, Richter P. Organizational changes and health complaints: An intervention study of the effects of transformational leadership. Unpublished manuscript. 2007.

192. Wolf S, Nebel-Töpfer C, Richter P. The relationship between transformational leadership and physical health. Unpublished article. 2007.

193. Wolf S, Nebel-Töpfer C, Richter P. Transformational leadership, job design and employees health: Differences between blue- and white-collar workers. Unpublished manuscript. 2007.

194. Wolf S, Nebel-Töpfer C, Richter P. Direct and indirect effects of four transformational leadership components on subordinates well-being. Unpublished manuscript. 2009.

195. Wolf S, Nebel-Töpfer C, Richter P. The impact of transformational leadership on followers' health: Exploring the effects on burnout, depression, anxiety and stress. Unpublished article. 2009.

196. Wolf S, Nebel-Töpfer C, Richter P. The effects of work characteristics and transformational leadership on followers' perceived health. Unpublished manuscript. 2010.

197. Wolf S, Nebel-Töpfer C, Richter P. The relationship between effort reward imbalance at work and health complaints- The mediating role of transformational leadership. Unpublished article. 2011.

198. Wolf S, Nebel-Töpfer C, Richter P. The role of transformational leadership in job demands-control and effort-reward imbalance model. Unpublished manuscript. 2011.

199. Wolfram H-J, Mohr G. Transformational Leadership, Team Goal Fulfillment, and Follower Work Satisfaction: The Moderating Effects of Deep-Level Similarity in Leadership Dyads. Journal of Leadership & Organizational Studies. 2009;15: 260–274. doi:10.1177/1548051808326595

200. Wu X, Mark H, Lee AJ, Yuan Y, Li S, Bi Y, et al. Positive spiritual climate supports transformational leadership as means to reduce nursing burnout and intent to leave. J Nurs Manag. 2020 [cited 23 Apr 2020]. doi:10.1111/jonm.12994

201. Yao Y-H, Fan Y-Y, Guo Y-X, Li Y. Leadership, work stress and employee behavior. Song Lin and Professor David Lamond P, editor. Chinese Management Studies. 2014;8: 109–126. doi:10.1108/CMS-04-2014-0089

202. Young, KH, Jong P, Yeon RS, Woo CS, Ah HM. The Effects of Preceptors' Transformational Leadership on Job Stress and Clinical Performance among New Graduate Nurses. Korean Journal of Health Policy & Administration. 2012;22: 347–364.

203. Zhang Y, LePine JA, Buckman BR, Wei F. It's Not Fair … Or Is It? The Role of Justice and Leadership in Explaining Work Stressor–Job Performance Relationships. AMJ. 2014;57: 675–697. doi:10.5465/amj.2011.1110

204. Zhu W, Avolio BJ, Walumbwa FO. Moderating Role of Follower Characteristics With Transformational Leadership and Follower Work Engagement. Group & Organization Management. 2009;34: 590–619. doi:10.1177/1059601108331242

205. Zwingmann I, Wegge J, Wolf S, Rudolf M, Schmidt M, Richter P. Is Transformational Leadership Healthy for Employees? A Multilevel Analysis in 16 Nations. German Journal of Human Resource Management: Zeitschrift für Personalforschung. 2014;28: 24–51. doi:10.1177/239700221402800103
