# Supplementary material for: The Mediating Effects of Work Characteristics on the Relationship between Transformational Leadership and Employee Well-Being: A Meta-Analytic Investigation
Source: Int J Environ Res Public Health. 2022 Mar 7;19(5):3133. doi: 10.3390/ijerph19053133 (PMC8910295; doi:10.3390/ijerph19053133)
Supplement: Supplementary file 1 [file ijerph-19-03133-s001.zip › Table S1.pdf]

Table S1. Search history.

<table>
<tr><th>Step</th><th>Search Medium</th><th>Search Terms</th><th>Results</th></tr>
<tr><td>1.</td><td>Search engines</td><td rowspan="8">S1: TI "Transformational leadership"
S2: SU "Transformational leadership"
S3: S1 OR S2
S4: AB "well-being" or "wellbeing" or "well being"
S5: SU "well-being" or "wellbeing" or "well being"
S6: SU "work engagement"
S7: S7 TI "work engagement"
S8: TI “flow” or “positive work orientation” or or “emotion based well-being” or “job attitudes” or “feelings towards ones job”, “flourishing”
S9: TI "burnout"
S10: SU "burnout"
S11: SU "mental health"
S12: TI "mental health"
S13: S4 OR S5 OR S6 OR S7 OR S8 OR S9 OR S10 OR S11 OR S12
S14: S3 AND S13</td><td></td></tr>
<tr><td rowspan="7"></td><td>PsycInfo</td><td>51</td></tr>
<tr><td>Psyndex</td><td>2</td></tr>
<tr><td>MEDLINE</td><td>5</td></tr>
<tr><td>PsychArticles</td><td>1</td></tr>
<tr><td>EconLit</td><td>3</td></tr>
<tr><td>BusinessSourcePremier</td><td>9</td></tr>
<tr><td>AcademicSearchPremier</td><td>8</td></tr>
<tr><td></td><td>PsycInfo, Psyndex, MEDLINE, PsychArticles; EconLit; BusinessSourcePremier; AcademicSearchPremier</td><td>S1: TI “leadership” instead of “Transformational leadership”

the rest as above; leadership terms and well-being terms separately</td><td>39</td></tr>
<tr><td></td><td>The Cochrane Library</td><td>“Transformational leadership” AND “well-being”; “burnout”; “work engagement”; “exhaustion”; “mental health”; “psychological complaints”; and variants of “leadership” and “burnout”, “psychological”, “well-being”, “mental”</td><td>0</td></tr>
<tr><td></td><td>DigiBib</td><td>“Transformational leadership” AND “well-being”; “burnout”; “work engagement”; “exhaustion”; “mental health”; “psychological complaints”; and variants of “leadership” and “burnout”, “psychological”, “well-being”, “mental”</td><td>7</td></tr>
<tr><td></td><td>PubPsych</td><td rowspan="2">“Transformational leadership” AND “well-being”; “burnout”; “work engagement”; “exhaustion”; “mental health”; “psychological complaints”; and variants of “leadership” and “burnout”, “psychological”, “well-being”, “mental”</td><td>3</td></tr>
<tr><td></td><td>PsychSpider</td><td>0</td></tr>
<tr><td></td><td>Science Direct</td><td>“Transformational leadership” AND “well-being”; “burnout”; “work engagement”;</td><td>4</td></tr>
</table>

Corresponding author: Friederike Teetzen, Department of Work and Organizational Psychology, University of Hamburg, friederike.teetzen@uni-hamburg.de

		"exhaustion"; "mental health"; "psychological complaints"; and variants of "leadership" and "burnout", "psychological", "well-being", "mental"	
2.	**Congress papers**		
	EAWOP Abstracts	"Transformational Leadership" and "well-being"	0
	Web of Science	"Transformational Leadership" and "well-being"; "leadership and "burnout"	10
3.	**Internet search engines**		
	Google Scholar	"Transformational leadership" AND "well-being"; "burnout"; "work engagement"; "exhaustion"; "mental health"; "psychological complaints"; "flow"; "work orientation"; "calling"; "job attitudes"; "feelings towards ones' job"; "flourishing"; and variants of "leadership" and "burnout", "psychological", "well-being", "mental"	34
	google.de		9
4.	**Dissertations**		
	German National Library (http://dbf-opac.ddb.de)	"Transformational Leadership" and "well-being"	0
	GVK (Gemeinsamer Verbund Katalog)	"Transformational leadership" AND "well-being"; "burnout"; "work engagement"; "exhaustion"; "mental health"; "psychological complaints"; and variants of "leadership" and "burnout", "psychological", "well-being", "mental"	2
	Dissertation Abstracts (ProQuest)	"Transformational leadership" AND "well-being"; "burnout"; "work engagement"; "exhaustion"; "mental health"; "psychological complaints"; and variants of "leadership" and "burnout", "psychological", "well-being", "mental"	13
	ABI-Inform	"transformational leadership" + "well-being"	0
5.	**Contacting authors**		8
6.	**Scanning relevant reference lists**		50
			**Total: 200 Studies**

Corresponding author: Friederike Teetzen, Department of Work and Organizational Psychology, University of Hamburg, friederike.teetzen@uni-hamburg.de
