# Supplementary material for: The Mediating Effects of Work Characteristics on the Relationship between Transformational Leadership and Employee Well-Being: A Meta-Analytic Investigation
Source: Int J Environ Res Public Health. 2022 Mar 7;19(5):3133. doi: 10.3390/ijerph19053133 (PMC8910295; doi:10.3390/ijerph19053133)
Supplement: Supplementary file 1 [file ijerph-19-03133-s001.zip › Table S3.pdf]

Table **S3. Coding Manual**

1. STUDY DESCRIPTION		
Number	Variable	Coding instructions
Missing data		Type in 999
1	Authors, Year	Type in "Authors, Year" with the first three author names, otherwise write "et al."
2	StudyID	Give a running number to each study included
3	ClusterID	Give a running number to each sample included
4	Quality	dis = dissertation<br>noi = no impact<br>Otherwise write the number of the impact, retrieved from the InCites Journal Citation Reports 2018 (https://apps.clarivate.com)
5	Type of publication	0 = unpublished<br>1 = published<br>2 = dissertation
6	Year	Type in the year of publication formatted like this '19XX'
7	continent	India<br>North America<br>South America<br>Europe<br>Northern Europe<br>Eastern Europe<br>Africa<br>Asia<br>Mixed<br>Middle East
8	Sample size	Sample size of the study
9	gender (female)	Type in the number of **female** participants
10	Industry	Education<br>Health care<br>Industry<br>MBA students (working at least 20 hours per week)<br>Mixed<br>Others<br>Public service<br>service
11	TFL measurement	MLQ<br>GTL<br>Raffgriff<br>TLI<br>other
12	TFL measurement	0 = MLQ<br>1 = other

13	TAL measurement	TAL measured<br>No TAL measured<br>Other measure
14	TAL measured	0 = no<br>1 = yes
15	PAL measurement	No PAL measured<br>PAL measured<br>Other measure
16	PAL measured	0 = no<br>1 = yes
17	AV	amwb = affective-motivational well-being<br>deex = depressed-exhaustive well-being<br>id = irritated-distressed well-being<br>plre = pleased-relaxed well-being<br>sc = psychosomatic complaints
18	subcategory	We (work engagement)<br>Depersonalization<br>Burnout<br>Depression<br>Emotional exhaustion<br>Irritation<br>Job stress<br>Negative affect<br>Positive affect<br>Reduced personal accomplishment<br>Somatic complaints<br>Subjective well-being
19	Variable name	Name of the outcome variable as indicated in the study
20	Instrument	The measurement instrument of the outcome variable
21	Item number	The item number of the measurement instrument used for the outcome variable
22	Exclusion	0 = no<br>1 = yes
23	Comment	Place a comment if you like

STATISTICS		
Number	Variable	Coding instructions
Missing data		Type in 999
14	Variance	Use the formula (1/n-3)
15	RelTFL	Type in the alpha value of TFL
16	Rel1	Type in the alpha value of the outcome variable
17	CorrTFL1	Type in the correlation value of TFL with the outcome variable
18	Mind.korr.TFL	Type in the corrected correlation value (Formula: corr/(Square root of product of reliabilities))

19	Fisherz.TFL	Use the Formula in Excel to produce a fisher's Z score
20	workc	Name the work characteristic measured in the study as named in the study
21	type	Job demand<br>Personal<br>Task-related<br>Organizational<br>Relational<br>Leadership
22	Workc.cd.hd	Cd = challenge demand<br>Hd = hindrance demand
23	Alpha.mediator1	Type in the alpha value of the work characteristic
	Cor.uv.workc	Correlation of TFL with the work characteristic
	Mind.korr.UV+M	Type in the corrected correlation value (Formula: corr/(Square root of product of reliabilities))
	Fisherz.UV+M	Use the Formula in Excel to produce a fisher's Z score
	Cor.av.workc	Correlation of outcome with the work characteristic
	Mind.korr.AV+M	Type in the corrected correlation value (Formula: corr/(Square root of product of reliabilities))
	Fisherz.AV+M	Use the Formula in Excel to produce a fisher's Z score
