# Supplementary material for: The Mediating Effects of Work Characteristics on the Relationship between Transformational Leadership and Employee Well-Being: A Meta-Analytic Investigation
Source: Int J Environ Res Public Health. 2022 Mar 7;19(5):3133. doi: 10.3390/ijerph19053133 (PMC8910295; doi:10.3390/ijerph19053133)
Supplement: Supplementary file 1 [file ijerph-19-03133-s001.zip › Table S4.pdf]

Authors, Year	sample size	country	economic sector	TFL measure	outcome variable	sub-category	variable name in study	type of work characteristic	work characteristic name in study
Abate et al., 2014	528	Spain	social service	MLQ	PRWB	prwb	well-being	PERS	occupational self-efficacy
Ali, 2015	203	USA	NGO	MLQ	DEWB	ee	emotional exhaustion	HD	role ambiguity
Altansukh, 2015	337	Mongolia	mixed	TLI	AMWB	we	work engagement		
Arfat et al, 2017	700	Pakistan	finance	other	AMWB	we	work engagement	REL	supportive culture
Arifin et al., 2014	251	Indonesa	education	MLQ	AMWB	we	work engagement	ORG	organizational culture
Ariyani & Hidayati, 2018	378	Indonesia	finance	n/a	AMWB	we	work engagement	TASK	innovative work behavior
Arnold & Walsh, 2015	215	n/a	industry	GTL	PRWB	prwb	psych. well-being	HD	customer incivility
					IDWB	na	negative affectivity	TASK	meaning of work
Arnold et al., 2007 (1)	319	Canada	health care	GTL	AMWB	posaff	pos. aff. well-being	TASK	meaningful work
Arnold et al., 2007 (2)	146	Canada	mixed	MLQ	PRWB	prwb	psych. well-being	TASK	meaningful work
Aryee et al., 2012	193	China	industry	MLQ	AMWB	we	work engagement	REL	LMX (relational)
								TASK	meaningfulness of work
Bae et al., 2013	280	USA	education	MLQ	AMWB	we	work engagement		
Bamberg et al., 2011	1958	Germany	mixed	MLQ	IDWB	irri	irritation		
Bass et al., 2016	728	USA	education	other	AMWB	we	work egagement		
					DEWB	ee			
Berger et al., 2019	501	Germany	industry	other	IDWB	js	strain	REL	team climate for learning
								TASK	autonomy
								HD	role ambiguity
Besieux et al., 2015	5313	Belgium	finance sector	MLQ	AMWB	we	work engagement		
Boerner et al., 2008	142	Germany	health care	other	IDWB	js		CD	stressors
Bohorquez, 2016	192	Colombia	education	MLQ	DEWB	ee	emotional exhaustion	ORG	organizational commitment
					DEWB	dep	depersonalization		

Authors, Year	sample size	country	economic sector	TFL measure	outcome variable	sub-category	variable name in study	type of work characteristic	work characteristic name in study
					DEWB	rpa	personal realization		
Bono et al., 2007	57	USA	health care	MLQ	AMWB	posaff	positive affect (happiness, enthusiasm, optimism)		
					IDWB	js	general stress (opposed to momentary due to day level)		
					IDWB	js	negative affect (anxiety, anger irritation)		
Breevaart et al., 2014a	61	Norway	naval cadets	MLQ	AMWB	we	work engagement	REL	social support
								TASK	autonomy
Breevaart et al., 2014b	162	Norway	navel cadets	TLI	AMWB	we	work engagement	TASK	job resources
Bright, 2018	116	USA	industry	MLQ	AMWB	we	work engagement		
Buck, 2018	111	USA	NGO	MLQ	AMWB	we	work engagement		
Bui et al., 2017	691	China	mixed	MLQ	AMWB	we	work engagement	TASK	person-job-fit
Buil et al., 2016	323	Spain	industry	GTL	AMWB	we	work engagement	ORG	organizational citizenship behavior employees
								PERS	organizational identification
Caniels et al., 2018	259	Netherlands	industry	TLS	AMWB	we	work engagement		
Carlstroem & Johanson, 2014	48	Sweden	industry	MLQ	PRWB	prwb	positive well-being		
Cheng et al., 2016	201	Australia	health care	MLQ	DEWB	burn	burnout	REL	social identity

Authors, Year	sample size	country	economic sector	TFL measure	outcome variable	sub-category	variable name in study	type of work characteristic	work characteristic name in study
Childers, 2019	196	USA	education	MLQ	AMWB	we	work engagement	PERS	occupational self-efficacy
					DEWB	ee	burnout	TASK	autonomy
					DEWB	dep	burnout		
					DEWB	rpa	personal realization		
Chua & Ayoko, 2019	155	Singapore	industry	MLQ	AMWB	we	work engagement	PERS	intrinsic motivation
Cole & Bedeian, 2007	828	USA	NGO	MLQ	DEWB	ee	emotional exhaustion		
Collier, 2004	44	USA	health care	MLQ	DEWB	ee	emotional exhaustion		
					DEWB	dep	depersonalization		
					DEWB	rpa	burnout		
Corrigan et al., 2003	620	USA	health care	MLQ	DEWB	ee	emotional exhaustion		
					DEWB	burn	reduced professional efficacy		
					DEWB	dep	depersonalization		
Daenzer, 2009	32	USA	industry	MLQ	IDWB	js	job stress		
Dartey-Baah & Ampofo, 2015	196	Ghana	financial sector	MLQ	IDWB	js			
Dibley et al., 2014	307	South Africa	public service	MLQ	AMWB	we	work engagement		
Diebig et al., 2017a	292	Germany	mixed	TLI	DEWB	burn	burnout	REL	team cooperation
					AMWB	we	work engagement		
Diebig et al., 2017b	205	Germany	mixed	TLI	IDWB	irri	stress	HD	role confict
Divya & Suganthi, 2018	336	India	industry	TLS	DEWB	burn	burnout	ORG	organizational justice
Dubinsky et al., 1995	174	USA	industry	MLQ	DEWB	burn	burnout	HD	role ambiguity
					IDWB	js	job stress	HD	role conflict

Authors, Year	sample size	country	economic sector	TFL measure	outcome variable	sub-category	variable name in study	type of work characteristic	work characteristic name in study
								PERS	personal commitment
Dunkl et al., 2015	212	Slovenia	industry	MLQ	DEWB	burn	loss of meaning/ burnout		
					IDWB	js	social emotional stress, performance-related stress		
Eghdamy et al., 2013	917	Iran	n/a	MLQ	DEWB	ee	emotional exhaustion		
Enwereuzor et al., 2018	224	Nigeria	mixed	TLI	AMWB	we	work engagement	TASK	person-job-fit
Espinoza-Parra et al., 2015	985	Chile	public service	MLQ	AMWB	we	work engagement	REL	social identity
Eyal & Roth, 2010	122	Israel	education	MLQ	DEWB	ee	burnout	PERS	autonomous motivation
Faupel & Suess, 2019	328	n/a	mixed	TLI	AMWB	we	work engagement	PERS	championing behavior
								PERS	occupational self-efficacy
Felfe & Schyns, 2004	213	Germany	mixed	MLQ	IDWB	irri	irritation		
					IDWB	js	stress		
Fernet et al., 2015 (1)	637	USA	health care	GTL	DEWB	ee		CD	job demands
								PERS	autonomous motivation
								PERS	occupational commitment
								TASK	job resources
Fernet et al., 2015 (2)	210	USA	education	GTL	DEWB	burn	psychological strain	PERS	autonomous motivation
								PERS	occupational commitment
								TASK	job resources
								CD	job demands

Authors, Year	sample size	country	economic sector	TFL measure	outcome variable	sub-category	variable name in study	type of work characteristic	work characteristic name in study
Franke & Felfe, 2011 (1)	201	Germany	industry	MLQ	IDWB	irri	perceived strain	PERS	affective organizational commitment
Franke & Felfe, 2011 (2)	509	Germany	mixed	MLQ	IDWB	irri	perceived strain	PERS	affective organizational commitment
Franke et al., 2012 (1)	136	Germany	mixed	MLQ	IDWB	irri	irritation		
Franke et al., 2012 (2)	82	Germany	public service	MLQ	IDWB	irri	irritation		
					IDWB	irri	irritation		
Franke et al., 2012 (3)	80	Germany	mixed	MLQ	IDWB	irri	irritation		
					IDWB	irri	irritation		
Franke et al., 2012 (4)	101	Germany	industry	MLQ	IDWB	irri	irritation		
					IDWB	irri	irritation		
Franke et al., 2012 (5)	795	Romania	mixed	MLQ	IDWB	irri	irritation		
					IDWB	irri	irritation		
Franke et al., 2012 (6)	201	Germany	health care	MLQ	IDWB	irri	irritation		
Franke et al., 2012 (7)	251	mixed	industry	MLQ	IDWB	irri	irritation		
Franke et al., 2012 (8)	195	Germany	public service	MLQ	IDWB	irri	irritation		
Franke et al., 2012 (9)	154	Germany	industry	MLQ	IDWB	irri	irritation		
Franke et al., 2014	535	Germany	mixed	MLQ	IDWB	irri	irritation		
Freeborough & Patterson, 2015	389	USA	NGO	MLQ	AMWB	we	work engagement		
Garcia-Rivera et al., 2013	102	Mexico	health care	MLQ	DEWB	ee	emotional exhaustion		
Garcia-Sierra & Fernandez-Castro, 2018	131	Spain	health care	MLQ	AMWB	we	work engagement (only vigor + dedication)	ORG	structural empowerment
Gautam & Enslin, 2019	191	South Africa	mixed	MLQ	AMWB	we	work engagement		
Gerards et al., 2018	656	Netherla nds	mixed	other	AMWB	we	work engagement	ORG	access to organizational knowledge

Authors, Year	sample size	country	economic sector	TFL measure	outcome variable	sub-category	variable name in study	type of work characteristic	work characteristic name in study
								ORG	freely accessible and open workplace
								REL	social interaction
								TASK	new ways of working facet 1 (autonomy)
Gerick, 2014	3018	Germany	education	MLQ	PRWB	prwb	habitual well-being	ORG	flow of information
					DEWB	ee	emotional exhaustion	ORG	participation
Ghadi et al., 2013	530	Australia	mixed	GTL	AMWB	we	work engagement	TASK	meaning in work
Ghislieri et al., 2013	125	Italy	public service	GTL	AMWB	we	work engagement		
Gilbert & Kelloway, 2018	3132	USA	mixed	GTL	PRWB	prwb	well-being	ORG	recognition
Gilbert et al., 2017	487	Canada	mixed	MLQ	DEWB	burn	burnout	PERS	personal resources
Gill et al., 2006	137	USA	industry	other	DEWB	ee	burnout		
					IDWB	js			
Gill et al., 2010	264	India	industry	other	IDWB	js			
Gillet et al., 2013	343	France	health care	GTL	AMWB	we	work engagement	ORG	distributive justice
								ORG	interactional justice
								ORG	organizational quality (quality of work life)
Gong et al., 2013	256	USA	education	MLQ	DEWB	rpa	reduced personal accomplishment		
					DEWB	ee	emotional exhaustion		
					DEWB	dep	depersonalization		
Gordick, 2002	296	USA	mixed	TLI	AMWB	posaff	positive affect (happiness, enthusiasm, optimism)	PERS	coping with organizational change

Authors, Year	sample size	country	economic sector	TFL measure	outcome variable	sub-category	variable name in study	type of work characteristic	work characteristic name in study
					IDWB	na	negative affect (anxiety, anger irritation)		
					IDWB	js	perceived stress		
Goswami et al., 2016	235	India	industry	TLI	AMWB	posaff	positive emotions at work	PERS	OCB
					AMWB	we	work egagement		
Green et al., 2013	388	USA	health care	MLQ	DEWB	ee	emotional exhaustion		
Green et al., 2014	285	USA	health care	MLQ	DEWB	ee	emotional exhaustion	HD	role overload
					DEWB	rpa	burnout	ORG	growth/advancement
					DEWB	dep	depersonalization	HD	role conflict
								REL	cooperation
								TASK	role clarity
Gregersen et al., 2011	461	Germany	health care	MLQ	DEWB	ee	emotional exhaustion	PERS	occupational self-efficacy
					DEWB	dep	depersonalization		
					IDWB	irri	irritation		
Gutierrez, 2014	430	USA	mixed	MLQ	AMWB	we	work engagement		
Hand et al., 2015	136	Australia	education	MLQ	IDWB	js	personal strain/stress	HD	role overload
								HD	role conflict
Hansen et al., 2014	451	USA	industry	TLS	AMWB	we	work engagement	PERS	organizational commitment
					IDWB	js	work tension	PERS	organizational identification
Hartling, 2019	278	USA	mixed	MLQ	DEWB	dep	cynicism		
					DEWB	rpa	professional efficacy		
					IDWB	js	stress		

Authors, Year	sample size	country	economic sector	TFL measure	outcome variable	sub-category	variable name in study	type of work characteristic	work characteristic name in study
					DEWB	ee	emotional exhaustion		
Hawkes et al., 2017	277	Australia	mixed	TLI	AMWB	we	work engagement	ORG	recognition
								TASK	influence at work
Hayati et al., 2014	240	Iran	health care	MLQ	AMWB	we	work engagement		
Hee et al., 2018	200	Malaysia	public service	MLQ	AMWB	we	employee engagement		
Henkel, 2012	148	USA	education	MLQ	AMWB	we	work engagement		
Hentrich et al., 2016	1074	Germany	health care	MLQ	IDWB	irri	cognitive irritation	HD	emotional demands
					IDWB	irri	emotional irritation	PERS	occupational self-efficacy
								CD	quantitative demands
Hetland et al., 2007	289	Norway	industry	MLQ	DEWB	dep	depersonalization		
					DEWB	burn	reduced professional efficacy		
					DEWB	ee	emotional exhaustion		
Hildenbrand et al., 2018	148	Germany	n/a	MLQ	DEWB	burn	burnout		
					AMWB	we	thriving		
					IDWB	na	negative affect		
Hofslett-Kopperud et al., 2014 (1)	1226	Norway	financial sector	MLQ	AMWB	we	work engagement	ORG	service climate
Hofslett-Kopperud et al., 2014 (2)	291	Norway	industry	MLQ	AMWB	we	work engagement		
Holstad et al., 2011 (1)	1255	Germany	mixed	GTL	AMWB	we	work engagement		
					IDWB	na	negative affect		
Holstad et al., 2011 (2)	437	Finland	mixed	GTL	AMWB	we	work engagement		
					IDWB	na	negative affect		
Holstad et al., 2011 (3)	552	Finland	mixed	GTL	AMWB	we	work engagement		

Authors, Year	sample size	country	economic sector	TFL measure	outcome variable	sub-category	variable name in study	type of work characteristic	work characteristic name in study
					IDWB	na	negative affect		
Holstad et al., 2013	2092	Germany	mixed	GTL	DEWB	ee	emotional exhaustion	ORG	procedural fairness
					IDWB	irri	cognitive irritation		
Holstad et al., 2014	199	Germany	financial sector	MLQ	IDWB	irri	emotional irritation	PERS	professional ambition
Holten et al., 2018	2947	Germany	mixed	GTL	PRWB	prwb	well-being		
Ilyavi, 2019	100	USA	education	MLQ	DEWB	dep	depersonalization		
					DEWB	ee	emotional exhaustion		
					DEWB	rpa	personal accomplishment		
Jacobs et al., 2013	315	Germany	industry	TLI	PRWB	prwb	well-being	HD	job demands
								CD	job demands physical
								TASK	decision latitude
								TASK	decision authority
Jansen in de Wal et al., 2020	687	Netherlands	education	other	AMWB	we	engagement in learning	CD	work pressure
								HD	emotional pressure
								PERS	autonomous motivation
								PERS	competence
								PERS	autonomy
								REL	relatedness
								REL	social support
								TASK	autonomy
Javeed & Farooqi, 2013	152	Pakistan	financial sector	GTL	AMWB	posaff	well-being	TASK	meaningful work
								TASK	influence
								ORG	involvement

Authors, Year	sample size	country	economic sector	TFL measure	outcome variable	sub-category	variable name in study	type of work characteristic	work characteristic name in study
Jena et al., 2018	511	India	industry	TLI	AMWB	we	employee engagement	ORG	organizational trust
Johnston, 2012 (1)	187	Canada	education	GTL	DEWB	depression	well-being		
					AMWB	posaff	well-being		
Johnston, 2012 (2)	915	Canada	mixed	GTL	PRWB	prwb	mental health		
Jordan, 2016	44	USA	industry	MLQ	AMWB	we	work engagement		
Kadian & Ahlawat, 2017	400	India	n/a	n/a	DEWB	rpa	reduced personal accomplishment	REL	social support
					DEWB	ee	emotional exhaustion	PERS	empowerment
					DEWB	dep	depersonalization		
Kanste et al., 2007	601	Finland	health care	MLQ	DEWB	ee	emotional exhaustion		
					DEWB	dep	depersonalization		
					DEWB	rpa	personal accomplishment		
Kara et al., 2013	443	Turkey	service sector	MLQ	DEWB	burn	emoloyee burnout	ORG	organizational quality
								PERS	organizational commitment
Kath et al., 2012	470	USA	health care	TLS	DEWB	dep	burnout	TASK	job control
					DEWB	ee	burnout	REL	coworker support
					AMWB	posaff	mental health	HD	role overload
					IDWB	js	perceptions of job stress	HD	role conflict
					IDWB	js	perceptions of job stress	HD	role ambiguity
					DEWB	rpa	burnout	TASK	predictability
								CD	non-participation

Authors, Year	sample size	country	economic sector	TFL measure	outcome variable	sub-category	variable name in study	type of work characteristic	work characteristic name in study
								HD	organizational constraints
								HD	interpersonal conflict
Kath et al., 2013	486	USA	health care	TLS	DEWB	dep	burnout	TASK	job control
					DEWB	ee	burnout	HD	role overload
					IDWB	js	perceptions of job stress	HD	role conflict
					IDWB	js	perceptions of job stress	HD	role ambiguity
					IDWB	na	negative affect	CD	non-participation
					DEWB	rpa	burnout	HD	organizational constraints
					AMWB	posaff	mental health	PERS	organizational commitment
								ORG	organizational support
								TASK	predictability
Kelloway et al., 2012 (1)	436	USA	industry	MLQ	PRWB	prwb	psychological well-being		
Kelloway et al., 2012 (2)	269	USA	mixed	MLQ	DEWB	depression	job related affective well-being		
					IDWB	na	job related affective well-being		
					AMWB	posaff	job related affective well-being		
					PRWB	prwb	job related affective well-being		
Kelloway et al., 2013	454	USA	health care	TLS	AMWB	posaff	mental health	PERS	affective commitment

Authors, Year	sample size	country	economic sector	TFL measure	outcome variable	sub-category	variable name in study	type of work characteristic	work characteristic name in study
Kensbock & Boehm, 2016	89	Israel	industry	MLQ	DEWB	ee	emotional exhaustion	PERS	organization-based self-esteem
Klaic et al., 2018	134	Germany	education	other	IDWB	js	work-related strain	PERS	need-supplies fit
Kranabetter & Niessen, 2016 (1)	206	n/a	finance	MLQ	DEWB	dep	cynicism	PERS	HoL (awareness)
					DEWB	ee	exhaustion		
Kranabetter & Niessen, 2016 (2)	247	n/a	industry	MLQ	DEWB	dep	cynicism	PERS	HoL (awareness)
					DEWB	ee	exhaustion	PERS	HoL (behavior)
Lam & O'Higgins, 2012	323	China	industry	MLQ	IDWB	js	job stress	PERS	organizational commitment - affective commitment
Lamade, 2019	194	USA	NGO	MLQ	AMWB	we	work engagement		
LaRocca et al., 2018	130	USA	veterans	MLQ	DEWB	depression	depression		
Laukamp, 2008	309	Germany	industry	MLQ	IDWB	js			
Lee & Cummings, 2008	172	USA	health care	other	DEWB	dep	depersonalization	TASK	managable workload
					DEWB	ee	emotional exhaustion	ORG	reward
					DEWB	burn	reduced professional efficacy	ORG	fairness
								ORG	values (meaningfulness/ organizaional commitment)
								REL	community
								TASK	control
Lee et al., 2019	500	China	mixed	TLI	AMWB	we	work engagement	ORG	performance feedback
Lewis & Cunningham, 2016	120	USA	health care	TLS	DEWB	ee	emotional exhaustion	TASK	managable workload

Authors, Year	sample size	country	economic sector	TFL measure	outcome variable	sub-category	variable name in study	type of work characteristic	work characteristic name in study
					AMWB	we	work engagement	ORG	fairness
								REL	community
								TASK	control
								TASK	reward
Li et al., 2019	281	China	industry	MLQ	AMWB	we	work engagement	PERS	empowerment
								PERS	innovative work behavior
Li, 2019	558	China	industry	MLQ	AMWB	we	work engagement	PERS	psychological capital
Liang et al., 2017	211	China	industry	MLQ	AMWB	we	work engagement	PERS	employee voice behavior
Lin et al, 2015	651	China	health care	MLQ	PRWB	prwb	general health		
Lin et al., 2019	130	USA	mixed	MLQ	AMWB	posaff	positive affect	PERS	OCB
					DEWB	ee	emotional exhaustion		
Liu et al., 2010	745	China	mixed	other	IDWB	js	stress symptoms	jd	work stress
Liu et al., 2019	385	China	public service	MLQ	DEWB	ee	occupational burnout	PERS	psychological empowerment
					DEWB	dep	occupational burnout		
					DEWB	rpa	occupational burnout		
Liu, 2012	107	USA	health care	MLQ	AMWB	we	job engagement		
Lozano-Chapa, 2017	109	USA	education	MLQ	DEWB	ee	emotional exhaustion		
Madathil et al., 2014	89	USA	health care	MLQ	DEWB	ee	emotional exhaustion	TASK	autonomy
					DEWB	rpa	personal accomplishment		
					DEWB	dep	depersonalization		
Malloy & Penprase, 2010	122	USA	health care	MLQ	DEWB	depressi on	depressive symptoms	HD	role conflict

Authors, Year	sample size	country	economic sector	TFL measure	outcome variable	sub-category	variable name in study	type of work characteristic	work characteristic name in study
					IDWB	js	behavioral and cognitive stress	CD	work demands
					DEWB	burn	burnout	CD	quantitative demands
								CD	cognitive demands
								HD	emotional demands
								HD	hiding emotion demands
								PERS	occupational self-efficacy
								REL	social support (colleagues)
								TASK	role clarity
								TASK	influence over work
								TASK	development possibilities (work)
								TASK	variation of work
								PERS	commitment
								TASK	meaning of work
Maricle, 2013	70	USA	Education	MLQ	DEWB	ee			
Mauno, 2016	3466	Finland	health care	GTL	AMWB	we	work engagement	HD	emotional labour
Mayr, 2017	213	Germany	NGO	MLQ	AMWB	we	work engagement		
Mazzetti et al., 2019	358	Italy	industry	MLQ	AMWB	we	work engagement	PERS	hardiness
					PRWB	prwb	general health		
McKee et al., 2011	178	Canada	health care	MLQ	PRWB	prwb	mental well-being	REL	sense of community
								TASK	meaningful work
Mehari, 2015	82	Sweden	health care	GTL	PRWB	prwb	well-being	TASK	meaningful work
Monje Amor et al., 2020	240	Spain	industry	TLS	AMWB	we	work engagement	ORG	structural empowerment
Moss, 2009	160	Australia	mixed	TLS	AMWB	we	dedication	PERS	self-esteem
					AMWB	we	vigor		

Authors, Year	sample size	country	economic sector	TFL measure	outcome variable	sub-category	variable name in study	type of work characteristic	work characteristic name in study
Mostafa, 2019	229	Great Britain	service sector	TLI	AMWB	we	work engagement	ORG	organizational social capital
Munir et al., 2010	447	Denmark	health care	GTL	AMWB	posaff	well-being	ORG	involvement
					DEWB	depression	depressive symptoms	PERS	occupational self-efficacy
								TASK	role clarity
								TASK	opportunities for development
								TASK	influence
								TASK	meaningful work
Nelson & Shraim, 2014	231	Kuwait	service sector	MLQ	AMWB	we	work engagement		
Nielsen & Daniels, 2012	425	Denmark	mixed	GTL	AMWB	we	vitality	jd	role conflict
					DEWB	burn	burnout	REL	social support
								REL	cohesion
								TASK	meaningful work
Niessen et al., 2017	200	Germany	education	MLQ	AMWB	we	thriving	PERS	proactivity
					DEWB	ee	emotional exhaustion		
Odink, 2014	191	mixed	mixed	other	AMWB	we	happiness (work related)	REL	relationships
					AMWB	we	health		
Othman, 2017	112	Malaysia	n/a	other	AMWB	we	work engagement		
Parveen & Adeinat, 2019	250	Saudi Arabia	finance sector	MLQ	DEWB	burn	burnout		
					IDWB	js	work stress		
Peng & Tseng, 2019	234	China	health care	MLQ	AMWB	we	work engagement		
Perilla Toro & Ortiz, 2017	597	Columbia, Mexico	mixed	MLQ	AMWB	posaff	positive affect	PERS	occupational self-efficacy

Authors, Year	sample size	country	economic sector	TFL measure	outcome variable	sub-category	variable name in study	type of work characteristic	work characteristic name in study
					PRWB	prwb	symptoms of discomfort		
					IDWB	na	negative affect		
Perko et al., 2014	557	Finland	public sector	GTL	DEWB	depression	depression	PERS	occupational self-efficacy
					IDWB	irri	work-related rumination	TASK	meaningfulness of work
Perko et al., 2016	262	Finland	public sector	GTL	AMWB	we	vigor		
					DEWB	ee	emotional exhaustion		
Prochazka et al., 2017	307	Czech Republic	mixed	other	AMWB	we	work engagement	PERS	occupational self-efficacy
Reddy & Mehta, 2019	150	India	Service sector	MLQ	DEWB	burn	burnout		
Rittschof & Fortunato, 2016	197	USA	mixed	MLQ	DEWB	ee	emotional exhaustion	PERS	affective commitment
					DEWB	rpa	personal accomplishment		
					DEWB	dep	depersonalization		
Rowold & Heinitz, 2008 (1)	101	Germany	industry	MLQ	IDWB	js	chronic stress		
Rowold & Heinitz, 2008 (2)	1311	Germany	public service	MLQ	IDWB	js	work-related stress		
Russell et al., 2014	379	USA	public service	TLI	DEWB	ee	emotional exhaustion	HD	stressors
					DEWB	dep	depersonalization		
					DEWB	rpa	reduced personal accomplishment		
Sahin et al., 2014	365		mixed	MLQ	AMWB	we	work engagement	ORG	organizatiol support
								PERS	psychological capital
								TASK	job control

Authors, Year	sample size	country	economic sector	TFL measure	outcome variable	sub-category	variable name in study	type of work characteristic	work characteristic name in study
								TASK	job clarity
								TASK	job competency
Sahu et al., 2018	405	India	industry	MLQ	AMWB	we	employee engagement	ORG	employer branding
Salanova et al., 2011	280	Spain	health care	MLQ	AMWB	we	vigour	PERS	occupational self-efficacy
					AMWB	we	dedication		
Salanova et al., 2014	702	Spain	mixed	TLI	DEWB	dep	cynicism	CD	quantitative overload
					IDWB	js	anxiety	jd	role ambiguity
					DEWB	depression	boredom	jd	role conflict
					AMWB	we	pleasure	jd	monotony
					DEWB	ee	fatigue	jd	emotional overload
					DEWB	rpa	professional competence	jd	mental overload
					AMWB	we	dedication	jd	mobbing
					AMWB	we	vigor	jd	emotional competences
					AMWB	we	absorption	ORG	organizational commitment
								PERS	occupational self-efficacy
								PERS	intrinsic interest
								PERS	inefficacy beliefs (recoded)
								REL	teamwork
								TASK	control
Salem, 2015	327	Egypt	service sector	MLQ	DEWB	burn	burnout		
					IDWB	js	job stress		

Authors, Year	sample size	country	economic sector	TFL measure	outcome variable	sub-category	variable name in study	type of work characteristic	work characteristic name in study
Schmitt et al., 2016	148	Netherlands	mixed	other	IDWB	js	job strain	PERS	personal initiative
					AMWB	we	work engagement		
Seltzer et al., 1989	277	USA	MBA students	MLQ	DEWB	burn	burnout		
					IDWB	js	job stress		
Sharma & Krishnan, 2012	93	India	mixed	other	AMWB	we	work engagement		
Shi et al., 2014	378	China	health care	TLI	DEWB	dep	cynicism		
					DEWB	ee	emotional exhaustion		
Shokory & Suradi, 2018	195	Malaysia	mixed	MLQ	AMWB	we	work engagement		
Skogstad et al., 2007	2273	Northern Norway	mixed		IDWB	js	psychologcial distress	HD	role conflict
								HD	conflicts with coworkers
								HD	bullying
								HD	role ambiguity
Smith, 2012	142	USA	health care	TLS	DEWB	burn	burnout	TASK	workload
					DEWB	burn	burnout	REL	community
					AMWB	we	work engagement	TASK	reward
								TASK	control
								ORG	fairness
								ORG	values
Song et al., 2012	432	Korea	mixed	MLQ	AMWB	we	work engagement		
Soo & Ali, 2013	474	Malaysia	financial sector	MLQ	IDWB	js	emotional stress		
					IDWB	js	psychogical stress		
Stein et al., 2019	356	Germany	education	GTL	IDWB	irri	irritation		
Stenling & Tafvelin, 2014	184	Sweden	athletes	other	AMWB	posaff	well-being	PERS	need satisfaction

Authors, Year	sample size	country	economic sector	TFL measure	outcome variable	sub-category	variable name in study	type of work characteristic	work characteristic name in study
Stordeur et al., 2001	625	Belgium	health care	MLQ	DEWB	ee	emotional exhaustion	HD	role ambiguity
								HD	role conflict
								CD	workload
								HD	social environment
Strom et al., 2014	348	USA	industry	MLQ	AMWB	we	work engagement	ORG	procedural justice
								ORG	distributive justice
Syrek et al., 2013	262	Germany	industry	TLS	DEWB	ee	emotional exhaustion	CD	time pressure
Tafvelin et al., 2011	101	Sweden	social service	MLQ	AMWB	posaff	affective well-being	ORG	climate for innovation
Tafvelin et al., 2019	217	Sweden	public service	GTL	DEWB	burn	burnout		
Taylor, 2014	78	USA	education	MLQ	AMWB	we	work engagement		
Teare, 2006	147	USA	service sector	other	IDWB	js	job stress		
					DEWB	burn	burnout		
Tims et al., 2011	45	Netherlands	industry	MLQ	AMWB	we	work engagement		
Tsai et al., 2009	282	Taiwan	industry	MLQ	AMWB	posaff	positive moods		
van Dick et al., 2018	5290	mixed	mixed	GTL	DEWB	burn	burnout	PERS	innovative work behavior
								REL	team identification
								PERS	OCB
van Dierendonck et al., 2014	200	Netherlands	education	TLS	AMWB	we	work engagement	jd	job uncertainty
								PERS	affective commitment
								PERS	psychological need satisfaction
Velkova, 2011	71	USA	industry	MLQ	PRWB	prwb	well-being		

Authors, Year	sample size	country	economic sector	TFL measure	outcome variable	sub-category	variable name in study	type of work characteristic	work characteristic name in study
Verbraak, 2014	119	mixed	mixed	other	AMWB	we	psychological well-being		
					AMWB	we	physical well-being		
					DEWB	ee	burnout		
Vignoli et al., 2018	492	Italy	industry	individualized consideration of MLQ	AMWB	we	work engagement	REL	social support (social well-being)
					PRWB	prwb	general health	HD	conflict with colleagues
Vila-Vázquez et al., 2018	320	Spain	industry	GTL	AMWB	we	work engagement	ORG	perceived organizational support
								PERS	core self-evaluations
Vincent-Hoeper, 2013	134	South Africa	mixed	GTL	DEWB	ee	emotional exhaustion	CD	quantitative demands
Vincent-Hoeper, 2013	618	Germany	health care		PRWB	prwb	affective well-being	TASK	predictability
Vincent-Hoeper et al., 2012	1132	Germany	mixed	MLQ	AMWB	we	work engagement	HD	role conflict
Vincent-Hoeper et al., 2017a	582	Germany	health care	MLQ	IDWB	irri	irritation	HD	hide emotions
					DEWB	dep	depersonalization	HD	job insecurity
					DEWB	ee	emotional exhaustion	TASK	role clarity
					PRWB	prwb	psychological well-being	ORG	influence at work
								ORG	closeness to organization
								REL	social support colleagues
								REL	feelings of community
								TASK	freedom of action

Authors, Year	sample size	country	economic sector	TFL measure	outcome variable	sub-category	variable name in study	type of work characteristic	work characteristic name in study
								TASK	possibilities for development
								TASK	meaning of work
Vincent-Hoeper et al., 2017b	1639	Germany	mixed	MLQ	IDWB	irri	irritation	CD	qualitative overload
					DEWB	ee	emotional exhaustion	CD	time pressure
					AMWB	posaff	positive well-being	HD	social stressors
								HD	insecurity
								HD	insufficient challenge
								PERS	occupational self-efficacy
								TASK	possibilities to present
								TASK	freedom of action
								TASK	development possibilities
Vincent-Hoeper et al., 2018	412	Germany	health care	GTL	IDWB	irri	irritation		
					DEWB	ee	emotional exhaustion		
					PRWB	prwb	subjective well-being		
					AMWB	we	work engagement		
Vincent-Hoeper et al., 2019 (1)	386	Germany	industry	MLQ	DEWB	ee	emotional exhaustion	PERS	occupational self-efficacy
					AMWB	posaff	positive well-being		
					IDWB	irri	irritation		
Vincent-Hoeper et al., 2019 (2)	306	USA	industry	MLQ	DEWB	ee		PERS	occupational self-efficacy
					IDWB	irri	irritation		
					AMWB	posaff	positive well-being		
					AMWB	we	work engagement		

Authors, Year	sample size	country	economic sector	TFL measure	outcome variable	sub-category	variable name in study	type of work characteristic	work characteristic name in study
Vincent-Hoeper et al., 2020	2015	Germany	mixed	GTL	AMWB	we	work engagement	CD	quantiative demands
					DEWB	ee	emotional exhaustion	CD	qualitative overexertion
					DEWB	ee	need for recovery	HD	illigitimate tasks
					IDWB	irri	emotional irritation	HD	uncertainty about the job
					IDWB	irri	cognitive irritation	lead	social support leader
					PRWB	prwb	psychologcial well-being	ORG	perceived organizational support
								PERS	need for personal development
								PERS	occupational ambition
								PERS	hope for success
								PERS	coping self-efficacy
								PERS	increasing structural resources
								PERS	supervisor crafting
								REL	social support colleagues
								TASK	possibilites for development
								TASK	role clarity
								TASK	autonomy
								TASK	perceived control
Walsh & Arnold, 2020	246	n/a	mixed	MLQ	IDWB	na	negative affect	PERS	mindfulness
					AMWB	we	psychological well-being (vigor)		
Walsh et al., 2014	254	USA	mixed	MLQ	PRWB	prwb	psychological health	ORG	procedural justice
								PERS	psychological empowerment

Authors, Year	sample size	country	economic sector	TFL measure	outcome variable	sub-category	variable name in study	type of work characteristic	work characteristic name in study
Wang et al., 2017	422	Taiwan	industry	TLI	AMWB	posaff	positive affect	PERS	resilience
					AMWB	we	work engagement		
Wefald et al., 2011	382	USA	industry	GTL	AMWB	we	work engagement	PERS	affective organizational commitment
					AMWB	we	work engagement	ORG	perceived organizational support
					AMWB	we	vigor		
					AMWB	posaff	positive affect		
Whitford & Moss, 2009	165	mixed	mixed	TLS	AMWB	we	work engagement	PERS	goal orientation learning
Widmann, 2013	224	USA	industry	MLQ	AMWB	we	work engagement		
Wolf et al., 2007a	545	Germany	mixed	MLQ	DEWB	ee	emotional exhaustion		
					PRWB	prwb	well-being		
Wolf et al., 2007b	390	Germany	mixed	MLQ	DEWB	ee	emotional exhaustion		
					PRWB	prwb	well-being		
					DEWB	rpa	burnout		
					DEWB	dep	cynicism		
Wolf et al., 2007c	76	Germany	mixed	MLQ	DEWB	ee	emotional exhaustion		
					PRWB	prwb	well-being		
Wolf et al., 2009a	29	Germany	mixed	MLQ	DEWB	ee	emotional exhaustion		
					DEWB	ee	inability to recover		
					PRWB	prwb	well-being		
Wolf et al., 2009b	25	Germany	mixed	MLQ	DEWB	ee	emotional exhaustion		
					PRWB	prwb	well-being		
Wolf et al., 2010	417	Germany	mixed	MLQ	PRWB	prwb	well-being		

Authors, Year	sample size	country	economic sector	TFL measure	outcome variable	sub-category	variable name in study	type of work characteristic	work characteristic name in study
					DEWB	ee	emotional exhaustion		
Wolf et al., 2011a	279	Germany	mixed	MLQ	DEWB	ee	emotional exhaustion		
					PRWB	prwb	well-being		
Wolf et al., 2011b	42	Germany	mixed	MLQ	DEWB	ee	emotional exhaustion		
					PRWB	prwb	well-being		
					DEWB	dep	cynicism		
					DEWB	rpa	burnout		
Wolf, 2012	100	Germany	mixed	MLQ	DEWB	ee	emotional exhaustion		
					DEWB	dep	cynicism		
					DEWB	rpa	burnout		
					PRWB	prwb	well-being		
Wolfram & Mohr, 2009	403	Germany	industry	MLQ	IDWB	irri	emotional irritation	PERS	occupational self-efficacy
								TASK	subjective meaning of work
Wu et al., 2019	391	China	health care	MLQ	DEWB	ee	emotional exhaustion		
Yao et al, 2014	347	China	mixed	MLQ	IDWB	js	work stress	PERS	deviant negative behavior (recoded)
Young et al., 2012	180	South Korea	health care	MLQ	IDWB	js	job stress		
Zhang et al., 2014	339	China	mixed	MLQ	DEWB	ee	emotional exhaustion	CD	challenge stressors
								HD	hindrance stressors
								ORG	organizational justice
Zhu et al., 2009	140	South Africa	mixed	MLQ	AMWB	we	work engagement	PERS	follower characteristics

Authors, Year	sample size	country	economic sector	TFL measure	outcome variable	sub-category	variable name in study	type of work characteristic	work characteristic name in study
Zwingmann et al., 2014 (1)	1438	Austria	mixed	MLQ	PRWB	prwb	well-being		
Zwingmann et al., 2014 (10)	297	Malaysia	mixed	MLQ	PRWB	prwb	well-being		
Zwingmann et al., 2014 (11)	335	Mexico	mixed	MLQ	PRWB	prwb	well-being		
Zwingmann et al., 2014 (12)	1509	Netherlands	mixed	MLQ	PRWB	prwb	well-being		
Zwingmann et al., 2014 (13)	1464	Slovakia	mixed	MLQ	PRWB	prwb	well-being		
Zwingmann et al., 2014 (14)	988	South Africa	mixed	MLQ	PRWB	prwb	well-being		
Zwingmann et al., 2014 (15)	1881	Spain	mixed	MLQ	PRWB	prwb	well-being		
Zwingmann et al., 2014 (16)	327	Switzerland	mixed	MLQ	PRWB	prwb	well-being		
Zwingmann et al., 2014 (2)	514	Brazil	mixed	MLQ	PRWB	prwb	well-being		
Zwingmann et al., 2014 (3)	2032	Croatia	mixed	MLQ	PRWB	prwb	well-being		
Zwingmann et al., 2014 (4)	290	Czech Republic	mixed	MLQ	PRWB	prwb	well-being		
Zwingmann et al., 2014 (5)	249	France	mixed	MLQ	PRWB	prwb	well-being		
Zwingmann et al., 2014 (6)	78339	Germany	mixed	MLQ	PRWB	prwb	well-being		
Zwingmann et al., 2014 (7)	620	Great Britain	mixed	MLQ	PRWB	prwb	well-being		
Zwingmann et al., 2014 (8)	3093	Hungary	mixed	MLQ	PRWB	prwb	well-being		
Zwingmann et al., 2014 (9)	200	Italy	mixed	MLQ	PRWB	prwb	well-being		

Note: MLQ = TFL measurement by Avolio & Bass, 1994, GTL = TFL measurement by Carless et al., 2000, TLI = TFL measurement by Podsakoff et al., 1996, TLS = TFL measurement by Rafferty & Griffin, 2004; AMWB = affective-motivational well-being, PRWB = pleased-relaxed well-being, DEWB = depressed-exhausted well-being, IDWB = irritated-distressed well-being, we = work engagement, ee = emotional exhaustion, dep = depersonalization, rpa = reduced personal accomplishment, js = job stress, irri = irritation, na = negative affect, posaff = positive affect; PERS = personal resource, TASK = task-related resource, REL = relational resource, ORG = organizational resource, CD = challenge demand, HD = hindrance demand
