# Supplementary material for: The Mediating Effects of Work Characteristics on the Relationship between Transformational Leadership and Employee Well-Being: A Meta-Analytic Investigation
Source: Int J Environ Res Public Health. 2022 Mar 7;19(5):3133. doi: 10.3390/ijerph19053133 (PMC8910295; doi:10.3390/ijerph19053133)
Supplement: Supplementary file 1 [file ijerph-19-03133-s001.zip › Table S5.pdf]

Table S3. Full Correlation Matrices for the SEM's.

Full Correlation Matrices for the SEM's.

1. The Job Resources Model

	TFL	AMWB	PRWB	DEWB	IDWB	PERS	TASK	ORG	REL
1. TFL	1								
2. AMWB	**.38**<br>(106, 121)<br>*(45,629)*	1							
3. PRWB	**.35**<br>(50, 51)<br>*(114,430)*	**.47**<br>(7, 11)<br>*(4,932)*	1						
4. DEWB	**-.27**<br>(87, 140)<br>*(39,005)*	**-.42**<br>(17, 44)<br>*(7,432)*	**-.44**<br>(9, 15)<br>*(8,136)*	1					
5. IDWB	**-.20**<br>(64, 75)<br>*(31,015)*	**-.30**<br>(15, 24)<br>*(7,426)*	**-.47**<br>(7, 8)<br>*(4,879)*	**.52**<br>(19, 33)<br>*(10,142)*	1				
6. PERS	**.27**<br>(61, 80)<br>*(28,434)*	**.41**<br>(33, 67)<br>*(12,329)*	**.31**<br>(7, 17)<br>*(4,588)*	**-.24**<br>(20, 54)<br>*(13,215)*	**-.22**<br>(20, 36)<br>*(10,862)*	1			
7. TASK	**.38**<br>(40, 71)<br>*(16,577)*	**.41**<br>(21, 41)<br>*(9,273)*	**.32**<br>(9, 17)<br>*(4,660)*	**-.28**<br>(17, 72)<br>*(7,834)*	**-.20**<br>(11, 38)<br>*(8,031)*	**.33**<br>(12, 48)<br>*(8,117)*	1		
8. ORG	**.51**<br>(37, 46)<br>*(22,263)*	**.41**<br>(24, 36)<br>*(10,516)*	**.33**<br>(5, 6)<br>*(9,121)*	**-.31**<br>(15, 42)<br>*(11,550)*	**-.20**<br>(6, 13)<br>*(6,338)*	**.33**<br>(11, 19)<br>*(6,565)*	**.43**<br>(14, 47)<br>*(6,700)*	1	
9. REL	**.42**<br>(28, 32)<br>*(17,126)*	**.36**<br>(15, 21)<br>*(8,082)*	**.27**<br>(6, 8)<br>*(4,394)*	**-.28**<br>(16, 36)<br>*(12,528)*	**-.21**<br>(8, 14)<br>*(5,422)*	**.27**<br>(7, 19)<br>*(10,041)*	**.31**<br>(17, 46)<br>*(8,301)*	**.39**<br>(10, 19)<br>*(5,786)*	1

2. The Job Demands Model

	TFL	AMWB	PRWB	DEWB	IDWB	CD	HD
1. TFL	1						
2. AMWB	**.38**<br>(106, 121)<br>*(45,629)*	1					
3. PRWB	**.35**<br>(50, 51)<br>*(114,430)*	**.47**<br>(7, 11)<br>*(4,932)*	1				
4. DEWB	**-.27**<br>(87, 140)<br>*(39,005)*	**-.42**<br>(17, 44)<br>*(7,432)*	**-.44**<br>(9, 15)<br>*(8,136)*	1			
5. IDWB	**-.20**<br>(64, 75)<br>*(31,015)*	**-.30**<br>(15, 24)<br>*(7,426)*	**-.47**<br>(7, 8)<br>*(4,879)*	**.52**<br>(19, 33)<br>*(10,142)*	1		
6. CD	**-.20**<br>(20, 23)<br>*(12,538)*	**-.15**<br>(7, 18)<br>*(5,128)*	**-.21**<br>(6, 9)<br>*(4,382)*	**.38**<br>(13, 25)<br>*(7,475)*	**.30**<br>(10, 20)<br>*(7,878)*	1	
7. HD	**-.28**<br>(24, 48)<br>*(17,789)*	**-.27**<br>(9, 32)<br>*(8,723)*	**-.31**<br>(7, 12)<br>*(4,565)*	**.36**<br>(14, 62)<br>*(7,393)*	**.37**<br>(13, 51)<br>*(10,718)*	**.30**<br>(12, 41)<br>*(9,395)*	1

3. The Comparison Model

	TFL	AMWB	PRWB	DEWB	IDWB	JD	JR	PERS
1. TFL	1							
2. AMWB	**.38**<br>(106, 121)<br>*(45,629)*	1						
3. PRWB	**.35**<br>(50, 51)<br>*(114,430)*	**.47**<br>(7, 11)<br>*(4,932)*	1					
4. DEWB	**-.27**<br>(87, 140)<br>*(39,005)*	**-.42**<br>(17, 44)<br>*(7,432)*	**-.44**<br>(9, 15)<br>*(8,136)*	1				
5. IDWB	**-.20**<br>(64, 75)<br>*(31,015)*	**-.30**<br>(15, 24)<br>*(7,426)*	**-.47**<br>(7, 8)<br>*(4,879)*	**.52**<br>(19, 33)<br>*(10,142)*	1			
6. JD	**-.25**<br>(33, 71)<br>*(21,247)*	**-.22**<br>(11, 50)<br>*(9,491)*	**-.28**<br>(10, 21)<br>*(5,648)*	**.38**<br>(20, 87)<br>*(9,649)*	**.36**<br>(16, 71)<br>*(11,628)*	1		
7. JR	**.45**<br>(78, 170)<br>*(43,093)*	**.39**<br>(44, 107)<br>*(18,298)*	**.30**<br>(14, 34)<br>*(12,207)*	**-.29**<br>(29, 167)<br>*(21,127)*	**-.20**<br>(17, 81)<br>*(11,468)*	**-.23**<br>(19, 221)<br>*(11,411)*	1	
8. PERS	**.27**<br>(61, 80)<br>*(28,434)*	**.41**<br>(33, 67)<br>*(12,329)*	**.31**<br>(7, 17)<br>*(4,588)*	**-.24**<br>(20, 54)<br>*(13,215)*	**-.22**<br>(20, 36)<br>*(10,862)*	**-.10**<br>(12, 72)<br>*(8,419)*	**.30**<br>(22, 115)<br>*(16,587)*	1

4. SEM with all work characteristics and one well-being indicator - AMWB

	TFL	AMWB	CD	HD	PERS	TASK	ORG	REL
1. TFL	1							
2. AMWB	**.38**<br>(106, 121)<br>*(45,629)*	1						
3. CD	**-.19**<br>(20, 23)<br>*(12,538)*	**-.13**<br>(7, 18)<br>*(5,128)*	1					
4. HD	**-.27**<br>(24, 48)<br>*(17,789)*	**-.24**<br>(9, 32)<br>*(8,723)*	**.32**<br>(12, 41)<br>*(9,395)*	1				
5. PERS	**.28**<br>(61, 80)<br>*(28,434)*	**.43**<br>(33, 67)<br>*(12,329)*	**-.01**<br>(8, 31)<br>*(7,425)*	**-.14**<br>(10, 41)<br>*(7,572)*	1			
6. TASK	**.39**<br>(40, 71)<br>*(16,577)*	**.41**<br>(21, 41)<br>*(9,273)*	**-.16**<br>(11, 35)<br>*(8,204)*	**-.23**<br>(12, 81)<br>*(8,468)*	**.35**<br>(12, 48)<br>*(8,117)*	1		
7. ORG	**.51**<br>(37, 46)<br>*(22,263)*	**.41**<br>(24, 36)<br>*(10,516)*	**-.24**<br>(6, 9)<br>*(4,585)*	**-.23**<br>(7, 21)<br>*(4,870)*	**.34**<br>(11, 19)<br>*(6,565)*	**.42**<br>(14, 47)<br>*(6,700)*	1	
8. REL	**.43**<br>(28, 32)<br>*(17,126)*	**.37**<br>(15, 21)<br>*(8,082)*	**-.15**<br>(6, 10)<br>*(4,933)*	**-.20**<br>(12, 39)<br>*(7,311)*	**.27**<br>(7, 19)<br>*(10,041)*	**.32**<br>(17, 46)<br>*(8,301)*	**.40**<br>(10, 19)<br>*(5,786)*	1

5. SEM with all work characteristics and one well-being indicator - PRWB

	TFL	PRWB	CD	HD	PERS	TASK	ORG	REL
1. TFL	1							
2. PRWB	**.35**							
	(50, 51)	1						
	(114,430)							
3. CD	**-.20**	**-.22**						
	(20, 23)	(6, 9)	1					
	(12,538)	*(4,382)*						
4. HD	**-.28**	**-.31**	**.30**					
	(24, 48)	(7, 12)	(12, 41)	1				
	(17,789)	*(4,565)*	*(9,395)*					
5. PERS	**.27**	**.31**	**-.03**	**-.15**				
	(61, 80)	(7, 17)	(8, 31)	(10, 41)	1			
	(28,434)	*(4,588)*	*(7,425)*	*(7,572)*				
6. TASK	**.38**	**.32**	**-.18**	**-.24**	**.33**			
	(40, 71)	(9, 17)	(11, 35)	(12, 81)	(12, 48)	1		
	(16,577)	*(4,660)*	*(8,204)*	*(8,468)*	*(8,117)*			
7. ORG	**.51**	**.33**	**-.26**	**-.24**	**.33**	**.40**		
	(37, 46)	(5, 6)	(6, 9)	(7, 21)	(11, 19)	(14, 47)	1	
	(22,263)	*(9,121)*	*(4,585)*	*(4,870)*	*(6,565)*	*(6,700)*		
8. REL	**.42**	**.28**	**-.16**	**-.22**	**.27**	**.30**	**.38**	
	(28, 32)	(6, 8)	(6, 10)	(12, 39)	(7, 19)	(17, 46)	(10, 19)	1
	(17,126)	*(4,394)*	*(4,933)*	*(7,311)*	*(10,041)*	*(8,301)*	*(5,786)*	

6. SEM with all work characteristics and one well-being indicator - DEWB

	TFL	DEWB	CD	HD	PERS	TASK	ORG	REL
1. TFL	1							
2. DEWB	**-.28**<br>(87, 140)<br>*(39,005)*	1						
3. CD	**-.21**<br>(20, 23)<br>*(12,538)*	**.37**<br>(13, 25)<br>*(7,475)*	1					
4. HD	**-.29**<br>(24, 48)<br>*(17,789)*	**.35**<br>(14, 62)<br>*(7,393)*	**.29**<br>(12, 41)<br>*(9,395)*	1				
5. PERS	**.27**<br>(61, 80)<br>*(28,434)*	**-.25**<br>(20, 54)<br>*(13,215)*	**-.04**<br>(8, 31)<br>*(7,425)*	**-.17**<br>(10, 41)<br>*(7,572)*	1			
6. TASK	**.37**<br>(40, 71)<br>*(16,577)*	**-.31**<br>(17, 72)<br>*(7,834)*	**-.20**<br>(11, 35)<br>*(8,204)*	**-.26**<br>(12, 81)<br>*(8,468)*	**.32**<br>(12, 48)<br>*(8,117)*	1		
7. ORG	**.50**<br>(37, 46)<br>*(22,263)*	**-.32**<br>(15, 42)<br>*(11,550)*	**-.28**<br>(6, 9)<br>*(4,585)*	**-.26**<br>(7, 21)<br>*(4,870)*	**.32**<br>(11, 19)<br>*(6,565)*	**.40**<br>(14, 47)<br>*(6,700)*	1	
8. REL	**.41**<br>(28, 32)<br>*(17,126)*	**-.29**<br>(16, 36)<br>*(12,528)*	**-.18**<br>(6, 10)<br>*(4,933)*	**-.24**<br>(12, 39)<br>*(7,311)*	**.26**<br>(7, 19)<br>*(10,041)*	**.29**<br>(17, 46)<br>*(8,301)*	**.37**<br>(10, 19)<br>*(5,786)*	1

7. SEM with all work characteristics and one well-being indicator - IDWB

	TFL	IDWB	CD	HD	PERS	TASK	ORG	REL
1. TFL	1							
2. IDWB	**-.20**<br>(64, 75)<br>*(31,015)*	1						
3. CD	**-.20**<br>(20, 23)<br>*(12,538)*	**.30**<br>(10, 20)<br>*(7,878)*	1					
4. HD	**-.28**<br>(24, 48)<br>*(17,789)*	**.38**<br>(13, 51)<br>*(10,718)*	**.30**<br>(12, 41)<br>*(9,395)*	1				
5. PERS	**.27**<br>(61, 80)<br>*(28,434)*	**-.20**<br>(20, 36)<br>*(10,862)*	**-.02**<br>(8, 31)<br>*(7,425)*	**-.15**<br>(10, 41)<br>*(7,572)*	1			
6. TASK	**.38**<br>(40, 71)<br>*(16,577)*	**-.19**<br>(11, 38)<br>*(8,031)*	**-.18**<br>(11, 35)<br>*(8,204)*	**-.24**<br>(12, 81)<br>*(8,468)*	**.33**<br>(12, 48)<br>*(8,117)*	1		
7. ORG	**.51**<br>(37, 46)<br>*(22,263)*	**-.20**<br>(6, 13)<br>*(6,338)*	**-.26**<br>(6, 9)<br>*(4,585)*	**-.24**<br>(7, 21)<br>*(4,870)*	**.33**<br>(11, 19)<br>*(6,565)*	**.40**<br>(14, 47)<br>*(6,700)*	1	
8. REL	**.42**<br>(28, 32)<br>*(17,126)*	**-.21**<br>(8, 14)<br>*(5,422)*	**-.16**<br>(6, 10)<br>*(4,933)*	**-.22**<br>(12, 39)<br>*(7,311)*	**.27**<br>(7, 19)<br>*(10,041)*	**.30**<br>(17, 46)<br>*(8,301)*	**.38**<br>(10, 19)<br>*(5,786)*	1

Note: first number in parentheses = number of studies, second number in parentheses = number of correlations, number in parentheses and italics = the n for this correlation, TFL = transformational leadership, AMWB = affective-motivational well-being, PRWB = pleased-relaxed well-being, DEWB = depressed-exhausted well-being, IDWB = irritated-distressed well-being, CD = challenge demands, HD = hindrance demands, PERS = personal resources, TASK = task-related resources, ORG = organizational resources, REL = relational resources, JD = job demands, JR = job resources.
